# Estimating the health effects of COVID-19-related immunisation disruptions in 112 countries during 2020–30: a modelling study

**DOI:** 10.1016/S2214-109X(23)00603-4

**Published:** 2024-03-12

**Authors:** Anna-Maria Hartner, Xiang Li, Susy Echeverria-Londono, Jeremy Roth, Kaja Abbas, Megan Auzenbergs, Margaret J de Villiers, Matthew J Ferrari, Keith Fraser, Han Fu, Timothy Hallett, Wes Hinsley, Mark Jit, Andromachi Karachaliou, Sean M Moore, Shevanthi Nayagam, Timos Papadopoulos, T Alex Perkins, Allison Portnoy, Quan Tran Minh, Emilia Vynnycky, Amy K Winter, Holly Burrows, Cynthia Chen, Hannah E Clapham, Aniruddha Deshpande, Sarah Hauryski, John Huber, Kevin Jean, Chaelin Kim, Jong-Hoon Kim, Jemima Koh, Benjamin A Lopman, Virginia E Pitzer, Yvonne Tam, Philipp Lambach, So Yoon Sim, Kim Woodruff, Neil M Ferguson, Caroline L Trotter, Katy A M Gaythorpe

**Affiliations:** a Medical Research Council Centre for Global Infectious Disease Analysis, Jameel Institute School of Public Health, Imperial College London, London, UK; b Section of Hepatology and Gastroenterology, Department of Metabolism, Digestion, and Reproduction, Imperial College London, London, UK; c London School of Hygiene & Tropical Medicine, London, UK; d Bloomberg School of Public Health, Johns Hopkins University, Baltimore, MD, USA; e Saw Swee Hock School of Public Health, National University of Singapore, Singapore; f Department of Epidemiology and Biostatistics and Center for the Ecology of Infectious Diseases, University of Georgia, Athens, GA, USA; g Center for Infectious Disease Dynamics, Pennsylvania State University, Pennsylvania, PA, USA; h School of Public Health, Yale University, New Haven, CT, USA; i Department of Biological Sciences, University of Notre Dame, Notre Dame, IN, USA; j Laboratoire Modélisation, épidémiologie, et surveillance des risques sanitaires and Unit Cnam risques infectieux et émergents, Institut Pasteur, Conservatoire National des Arts et Metiers, Paris, France; k Veterinary Medicine, University of Cambridge, Cambridge, UK; l Rollins School of Public Health, Emory University, Atlanta, GA, USA; m International Vaccine Institute, Seoul, South Korea; n UK Health Security Agency, London, UK; o Center for Health Decision Science, T H Chan School of Public Health, Harvard University, Boston, MA, USA; p School of Tropical Medicine and Global Health, Nagasaki University, Nagasaki, Japan; q School of Medicine, Washington University, St Louis, MO, USA; r Oxford University Clinical Research Unit, Ho Chi Minh City, Viet Nam; s Nuffield Department of Medicine, Oxford University, Oxford, UK; t School of Public Health, University of Hong Kong, Hong Kong Special Administrative Region, China; u Department of Immunization, Vaccines, and Biologicals, WHO, Geneva, Switzerland; v Centre for Artificial Intelligence in Public Health Research, Robert Koch Institute, Wildau, Germany

## Abstract

**Background:**

There have been declines in global immunisation coverage due to the COVID-19 pandemic. Recovery has begun but is geographically variable. This disruption has led to under-immunised cohorts and interrupted progress in reducing vaccine-preventable disease burden. There have, so far, been few studies of the effects of coverage disruption on vaccine effects. We aimed to quantify the effects of vaccine-coverage disruption on routine and campaign immunisation services, identify cohorts and regions that could particularly benefit from catch-up activities, and establish if losses in effect could be recovered.

**Methods:**

For this modelling study, we used modelling groups from the Vaccine Impact Modelling Consortium from 112 low-income and middle-income countries to estimate vaccine effect for 14 pathogens. One set of modelling estimates used vaccine-coverage data from 1937 to 2021 for a subset of vaccine-preventable, outbreak-prone or priority diseases (ie, measles, rubella, hepatitis B, human papillomavirus [HPV], meningitis A, and yellow fever) to examine mitigation measures, hereafter referred to as recovery runs. The second set of estimates were conducted with vaccine-coverage data from 1937 to 2020, used to calculate effect ratios (ie, the burden averted per dose) for all 14 included vaccines and diseases, hereafter referred to as full runs. Both runs were modelled from Jan 1, 2000, to Dec 31, 2100. Countries were included if they were in the Gavi, the Vaccine Alliance portfolio; had notable burden; or had notable strategic vaccination activities. These countries represented the majority of global vaccine-preventable disease burden. Vaccine coverage was informed by historical estimates from WHO–UNICEF Estimates of National Immunization Coverage and the immunisation repository of WHO for data up to and including 2021. From 2022 onwards, we estimated coverage on the basis of guidance about campaign frequency, non-linear assumptions about the recovery of routine immunisation to pre-disruption magnitude, and 2030 endpoints informed by the WHO Immunization Agenda 2030 aims and expert consultation. We examined three main scenarios: no disruption, baseline recovery, and baseline recovery and catch-up.

**Findings:**

We estimated that disruption to measles, rubella, HPV, hepatitis B, meningitis A, and yellow fever vaccination could lead to 49 119 additional deaths (95% credible interval [CrI] 17 248–134 941) during calendar years 2020–30, largely due to measles. For years of vaccination 2020–30 for all 14 pathogens, disruption could lead to a 2·66% (95% CrI 2·52–2·81) reduction in long-term effect from 37 378 194 deaths averted (34 450 249–40 241 202) to 36 410 559 deaths averted (33 515 397–39 241 799). We estimated that catch-up activities could avert 78·9% (40·4–151·4) of excess deaths between calendar years 2023 and 2030 (ie, 18 900 [7037–60 223] of 25 356 [9859–75 073]).

**Interpretation:**

Our results highlight the importance of the timing of catch-up activities, considering estimated burden to improve vaccine coverage in affected cohorts. We estimated that mitigation measures for measles and yellow fever were particularly effective at reducing excess burden in the short term. Additionally, the high long-term effect of HPV vaccine as an important cervical-cancer prevention tool warrants continued immunisation efforts after disruption.

**Funding:**

The Vaccine Impact Modelling Consortium, funded by Gavi, the Vaccine Alliance and the Bill & Melinda Gates Foundation.

**Translations:**

For the Arabic, Chinese, French, Portguese and Spanish translations of the abstract see Supplementary Materials section.

## Introduction

There have been notable declines in immunisation coverage due to the COVID-19 pandemic worldwide, with the disruption varying geographically, by vaccine, and by delivery method. Low-income and middle-income countries had substantial disruption in routine and supplementary immunisation activities (SIAs) in 2020; in routine immunisation, the median relative percentage change was –10·8% across all vaccines in 45 countries.^1^ An additional 3·5 million zero-dose children missed out on diphtheria, tetanus, and pertussis dose 1 (DTP) compared with 2019.^2^ These disruptions led to increasing disease burden and outbreak risk and anticipated future public health disadvantages.^3^

**Research in context**

**Evidence before this study**

We searched PubMed between Dec 1, 2019, and Oct 6, 2023, for studies published in English using the search terms (“COVID-19” OR “SARS-CoV-2”) AND (“immunisation” OR “vaccination”) AND (“disruption” OR “delay*” OR “postpon*”). Original research studies were included if they focused on disruption to vaccination activities due to the COVID-19 pandemic in low-income and middle-income countries. We found 4125 studies, of which 82 met the inclusion criteria. These studies showed evidence of notable declines in immunisation activities across the globe related to the COVID-19 pandemic. These declines included reductions in achieved routine coverage, cancellation or postponement of campaigns, and identification of under-immunised cohorts. Immunisation was most disrupted in the early months of the pandemic, particularly March to May, 2020; however, recovery after disruptions varied by country, age group, and vaccine. Many countries observed substantial subnational variation. Although many countries observed partial recovery once lockdown policies ended in 2020, disruption in many countries continued into 2021. Furthermore, clinician staff shortages and vaccine stock-outs, caused by supply-chain disruptions, contributed to immunisation delays. Despite the far-reaching ramifications of these immunisation disruptions, including potential increases in disease burden and excess deaths, there are still uncertainties in the way to recovery and catch-up. Few studies examine disruption post-2020 or how disruptions in vaccination have translated into estimates of increased burden, changes in vaccine effects, or vaccine prioritisation.

**Added value of this study**

Our study is the first large-scale evaluation of vaccine effects, in terms of morbidity and mortality averted, since the first COVID-19 disruption data for 2021 became available through projected estimates and the WHO–UNICEF Estimates of National Immunization Coverage were published in July, 2022. We showed the magnitude of the change in effect, tracked through under-immunised cohorts and immunisation-coverage disruptions, and estimated the duration of disruption given best estimates of future vaccine coverage for 14 pathogens. We also identified regions and cohorts that could especially benefit from catch-up vaccination, either because their projected burden was higher than expected compared with a no-disruption scenario or because catch-up activities averted a majority of the additional burden.

**Implications of all the available evidence**

The effects of the COVID-19 pandemic on immunisation activities varied by geographical region, vaccine, and schedule, contributing to complex prioritisation of vaccine efforts. Our study emphasises the importance of timely vaccination given disease-specific differences in the timeline of burden and identifies cohorts and regions that could particularly benefit from catch-up vaccination. Our findings also reiterate the enormous benefits of sustained effort in vaccination.

The effects of COVID-19 on routine-immunisation delivery lingered in 2021; globally, routine-immunisation coverage declined in every region, with 25 million infants missing out on one or more doses of DTP vaccine, an additional 6 million compared with 2019.^4^ During the fourth quarter of 2021, disruptions to immunisation services were reported by 53% of countries subsequently included in this study, representing an 11% increase from the third quarter. Furthermore, many SIAs, which are crucial in achieving vaccine-coverage goals and reaching people missed by routine immunisation, were postponed. For example, 18 measles campaigns that had been postponed since 2020 still had not been conducted by 2021.^5^ The COVID-19 pandemic led to the largest sustained drop in immunisation in the past three decades, exacerbated by resource diversion and supply chain disruptions.^4^, ^6^ Additionally, social, political, and economic disruptions after the pandemic have the potential to result in continued low vaccine coverage and under-immunised cohorts in the future if there is no concerted global effort to reverse this decline.^7^ Although partial recovery was observed in some countries beginning in 2021, it was variable by geography, demographic, and vaccine.^8^

Before 2020, immunisation efforts, including progress on the Global Vaccine Action Plan 2011–20,^9^ plateaued in terms of coverage of the third dose of DTP and the first dose of measles-containing vaccine.^9^ More than 19 million children in 2019 still did not have a full course of DTP vaccine; these children often lived in low-income households in which families had little access to formal education, highlighting the persistent inequities in immunisation access.^2^, ^9^, ^10^ Furthermore, increasing vaccine hesitancy, especially after the start of the COVID-19 pandemic, has contributed to declines in childhood-vaccine demand. There was a measurable drop in confidence in 46 of 55 countries.^11^, ^12^ Addressing these existing immunity and coverage gaps will require a change in vaccination strategies, an increase in further immunisation catch-ups through campaigns or intensified routine-immunisation activities, and strong political commitment to the WHO Immunization Agenda 2030. Future efforts by global partners to restore coverage losses were announced in April, 2023.^13^

This study is the first large-scale evaluation of the effect of disruptions in vaccine coverage, in terms of morbidity and mortality averted, since the WHO–UNICEF Estimates of National Immunization Coverage (WUENIC), capturing the effects of coverage disruption, were published in July, 2022.^6^ We included projections for 14 antigens: typhoid, measles, meningitis A, hepatitis B, human papillomavirus (HPV), *Streptococcus pneumoniae* (prevented by the pneumococcal conjugate vaccine), *Haemophilus influenzae* type b (HIB), rotavirus, Japanese encephalitis, yellow fever, rubella, diphtheria, tetanus, and pertussis, with further analysis on outbreak-prone or priority diseases. We aimed to quantify the effects of vaccine-coverage disruption on routine and campaign immunisation services, identify cohorts and regions that could particularly benefit from catch-up activities,^14^ and establish the extent to which losses in effect could be recovered.

## Methods

### Study design

For this modelling study, we used modelling groups from the Vaccine Impact Modelling Consortium (VIMC). The VIMC has been producing estimates of the health effects of multiple vaccines since September, 2017, with an emphasis on robust and rigorous modelling. This Article provides two sets of modelling estimates, one with vaccine-coverage data from 1937 to 2021 for a subset of vaccine-preventable, outbreak-prone or priority diseases (ie, measles, rubella, hepatitis B, HPV, meningitis A, and yellow fever) to examine mitigation measures, hereafter referred to as recovery runs. These diseases and vaccines were chosen as they have high epidemic potential (ie, high relative transmission or population susceptibility, are priorities for vaccine introduction, or have high vaccine effects). The second set of estimates were conducted with vaccine-coverage data from 1937 to 2020, used to calculate effect ratios (ie, the burden averted per dose) for all 14 included vaccines and diseases, hereafter referred to as full runs. Both runs were modelled from Jan 1, 2000, to Dec 31, 2100. Countries were included if they were in the Gavi, the Vaccine Alliance portfolio; had notable burden; or had notable strategic vaccination activities (appendix 6 p 17).

All data were from secondary sources and did not require ethical approval.

### Vaccination scenarios

**Figure 1:**
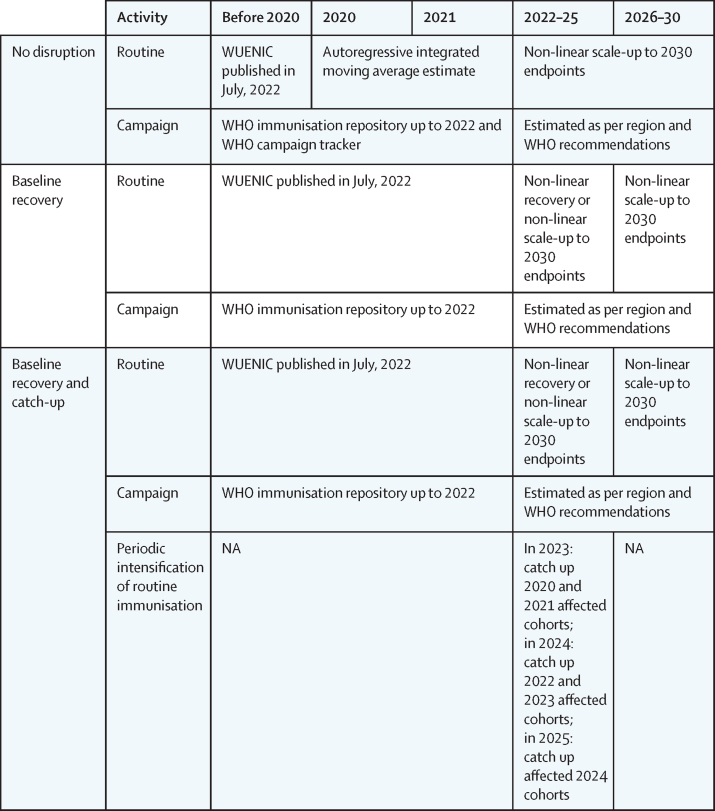
Summary of recovery vaccine-coverage scenarios NA=not available. WUENIC=WHO–UNICEF Estimates of National Immunization Coverage.

We examined three main scenarios: no disruption, baseline recovery, and baseline recovery and catch-up (figure 1). The baseline recovery and catch-up scenario included the catch-up activity of intensified routine vaccination (also known as periodic intensification of routine immunisation), informed by WHO guidance.^14^

Vaccine coverage was informed by historical estimates from WUENIC (as of 2022), the WHO immunisation repository (as of July 15, 2022) for data up to and including 2021, and the Gavi data repository.^6^, ^15^, ^16^ From 2022 onwards, we estimated coverage on the basis of guidance about campaign frequency, non-linear assumptions about the recovery of routine immunisation to pre-disruption magnitude, and 2030 endpoints informed by the WHO Immunization Agenda 2030 aims and expert consultation. The non-linear recovery to pre-pandemic immunisation coverage followed a logistic functional form that was parameterised such that the greatest recovery rate was seen in 2023 and the endpoints were within 1% of the actual, or target, coverage (appendix 6 pp 2–8). In the no-disruption scenario, to estimate coverage in the hypothetical absence of COVID-19-related disruption, routine immunisation was projected for 2020 and 2021 with an autoregressive integrated moving average with logit transform for each country–vaccine pair; a similar approach has been validated for DTP and measles-containing vaccine.^17^ We also included vaccination campaigns that were planned but postponed or cancelled due to the COVID-19 pandemic, according to the WHO campaign tracker.^15^ The introduction of birth-dose hepatitis B in the WHO African region was planned in 2019–21 but did not occur. In that scenario, we assumed introduction occurred in 2022; future introductions were estimated to align with decadal endpoints for DTP (appendix 6 p 1).

For all scenarios, WHO regions were used (ie, African region, Eastern Mediterranean region, European region, region of the Americas, South-East Asia region, and Western Pacific region; appendix 6 p 9).

### Input data

All modelling groups in the VIMC use standardised, national, age-stratified demographic data from the UN World Population Prospects (UNWPP), either released on July 15, 2022 (for the recovery runs), or July 15, 2019 (for the full runs). Our final results were scaled to UNWPP 2022 data, for consistency.

Each VIMC group generates estimates of burden under various vaccination scenarios, in which burden is defined as deaths, cases, or disability-adjusted life-years. The VIMC secretariat then uses these estimates of burden to calculate vaccine effect. There were 21 modelling groups included in VIMC at the time of our analysis, with two per disease (excluding HIB and meningitis A, which had one modelling group each). The aim of the use of two models per disease was to include structural uncertainty in resulting estimates. Model characteristics varied by group and vaccine and included static and dynamic transmission models (appendix 6 pp 45–69).

Each group was asked to provide 200 estimates of burden for each year, vaccination scenario, and country. Each run used a different sample of input parameters taken from associated distributions, defined by each modelling group, to reflect uncertainty. All vaccination scenarios were compared for the same set of model parameters. The mean, median, and credible intervals (CrIs) were calculated by combining the full probabilistic distributions of effect for all models for a pathogen. For estimates by calendar year, including catch-up activities, only dynamic transmission models or models of outbreak-prone or priority pathogens were included (ie, measles, rubella, hepatitis B, HPV, meningitis A, and yellow fever), but the same approach was taken regarding uncertainty quantification. Estimates for meningitis A by calendar year were calculated for a subset of five countries (ie, Burkina Faso, Chad, Mali, Niger, and Ethiopia), which were included as they have high burden and potential disruption.

### Analysis and effect calculations

We calculated two main outputs: excess burden due to vaccine-coverage disruption and mitigated burden due to catch-up activities. Excess burden due to coverage disruption was calculated by examining the difference between the baseline recovery and no-disruption scenarios; the mitigated burden due to catch-up activities was calculated by examining the difference between the baseline recovery and baseline recovery and catch-up scenarios. We calculated the burden averted due to vaccination by calendar year and by year of vaccination.^18^

Effect by year of vaccination measured the lifetime benefit for individuals who were immunised in a particular year.^18^ To calculate the effect by year of vaccination, we estimated effect ratios and stratified them by activity type, country, modelling group, and vaccine. Effect ratios were calculated with the full model runs for all diseases in the VIMC plus those calculated for diphtheria, tetanus, and pertussis by the Immunization Agenda 2030 project team^19^ by comparing a no-vaccination and with-vaccination scenario. These effect ratios were applied to new coverage projections to extrapolate effect. This approach produces a good approximation of the effect given the new coverage assumptions when projecting estimates of vaccine effect for static models of endemic disease with relatively small variations in coverage. However, it provides less accurate estimates for outbreak-prone diseases with dynamic models; the performance of this approach was examined separately by comparing between projected and modelled scenarios, for example vaccines and countries.^18^ As such, we also estimated calendar-year estimates for a subset of outbreak-prone or priority diseases. Effect by calendar year was calculated directly from model runs by comparing two scenarios (ie, baseline recovery and no-disruption or baseline recovery and baseline recovery and catch-up) and captured the short-term immediate effects of vaccination. However, it did not capture the effects for diseases in which the burden occurs later in life during this time (ie, 2020–30). Through both effects, calendar year and year of vaccination, we could compare the relative benefits of vaccination during the short and long term, as well as the wider effects of disruption.

Effect was estimated and manuscript analyses were conducted with R version 4.1.0.

### Role of the funding source

The funders of the study provided feedback on the vaccination-coverage scenario assumptions and reviewed this Article before publication. The funders of the study had no role in data collection, data analysis, data interpretation, or writing of the report.

## Results

**Figure 2:**
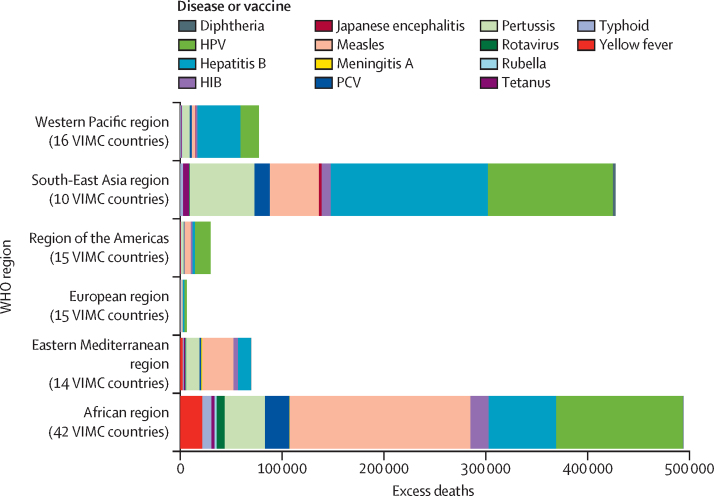
Mean estimated additional deaths due to vaccine-coverage disruption by region (years of vaccination 2020–30) Calculated by comparing the baseline recovery and no-disruption scenarios (appendix 6 pp 2–8). HIB=*Haemophilus influenzae* type b. HPV=human papillomavirus. PCV=pneumococcal conjugate vaccine. VIMC=Vaccine Impact Modelling Consortium.

We estimated that 49 119 additional deaths (95% CrI 17 248–134 941) might occur between the calendar years of 2020 and 2030 due to vaccine-coverage disruption to measles, HPV, yellow fever, hepatitis B, rubella, and meningitis A. 90·68% of excess deaths were due to measles and 7·53% were due to yellow fever (table 1; figure 2). Although meningitis A was modelled for a subset of countries, these results suggested little to no excess burden. For HPV and hepatitis B, the burden of disease occurred later in life and was therefore unlikely to be captured in this time. Some estimated negative excess deaths could have occurred due to cumulative rounding differences between scenarios and stochastic differences in simulations (appendix 6 p 32).

**Table 1:** Additional deaths due to vaccine-coverage disruption (calendar years 2020–30)

	**African region**	**Eastern Mediterranean region**	**European region**	**Region of the Americas**	**South-East Asia region**	**Western Pacific region**	**Total**
HPV	96 (86 to 106)	0 (0 to 0)	1 (1 to 1)	13 (12 to 15)	−86 (−123 to −53)	3 (2 to 4)	26 (−12 to 61)
Hepatitis B	315 (279 to 354)	15 (9 to 22)	0 (0 to 0)	0 (0 to 0)	122 (95 to 143)	67 (48 to 82)	518 (456 to 572)
Measles	26 498 (4884 to 79 034)	2918 (927 to 13 999)	642 (283 to 1360)	1389 (74 to 3849)	12 899 (5529 to 35 276)	199 (−221 to 1565)	44 544 (13 794 to 130 657)
Meningitis A	2 (0 to 11)	..	..	..	..	..	2 (0 to 11)
Rubella	2 (−70 to 47)	14 (−265 to 287)	264 (−1 to 946)	30 (−2 to 160)	19 (−5 to 75)	0 (−15 to 13)	329 (−107 to 1148)
Yellow fever	3499 (1247 to 6817)	217 (50 to 701)	..	−15 (−58 to 18)	..	..	3701 (1455 to 7003)
Total	30 410 (9033 to 83 115)	3163 (973 to 14 568)	906 (318 to 1794)	1417 (77 to 3907)	12 954 (5551 to 35 321)	268 (−162 to 1629)	49 119 (17 248 to 134 941)

Data are n (95% CrI). Calculated by comparing the baseline recovery and no-disruption scenarios. Meningitis A was run for five countries only. There are 42 VIMC countries in the WHO African region, 14 VIMC countries in the Eastern Mediterranean region, 15 VIMC countries in the European region, 15 VIMC countries in the region of the Americas, 10 VIMC countries in the South-East Asia region, and 16 VIMC countries in the Western Pacific region. CrI=credible interval. HPV=human papillomavirus. VIMC=Vaccine Impact Modelling Consortium.

Excess burden for years of vaccination 2020–30 for all vaccines tracked individuals through their lifetime after vaccinations (figure 2). Therefore, the excess burden of hepatitis B and HPV is emphasised. This finding highlights that although the immediate excess burden of measles and yellow fever was more apparent in the calendar-year view, the long-term implications for diseases with morbidity occurring later in life are still substantial. Overall we estimated 967 635 additional deaths (95% CrI 896 596–1 049 981) due to disruption in years of vaccination 2020–30, assuming recovery takes until 2025.

Excess burden due to disruption was mostly driven by variation in routine-immunisation activities. For vaccines for which delivery was through both routine and campaign activities (ie, HPV, Japanese encephalitis, measles, meningitis A, rubella, typhoid, and yellow fever), routine-immunisation disruption generally accounted for the majority of additional burden (appendix 6 p 19). The exception was rubella, for which the majority of the additional burden was attributable to disrupted campaign activities.

We estimated that, in the hypothetical absence of a pandemic, immunisation activities taking place between 2020 and 2030 could avert 37 378 194 deaths (95% CrI 34 450 249–40 241 202). By contrast, with disruptions, we estimated that 36 410 559 deaths (33 515 397–39 241 799) could be averted during the same time. This reduction is 2·66% (2·52–2·81) of vaccine effects during the time due to disruptions in coverage.

We estimated that 18 321 deaths (95% CrI 6246–58 522) could be averted between calendar years 2020 and 2030 as a result of catch-up activities for measles, HPV, yellow fever, hepatitis B, rubella, and meningitis A, the majority in the WHO African and South-East Asia regions for measles (table 2).

**Table 2:** Mean mitigated deaths per WHO region due to catch-up activities (calendar years 2020–30)

	**African region**	**Eastern Mediterranean region**	**European region**	**Region of the Americas**	**South-East Asia region**	**Western Pacific region**	**Total**
HPV	143 (116 to 176)	0 (0 to 0)	1 (0 to 1)	23 (20 to 26)	95 (53 to 137)	4 (2 to 6)	266 (206 to 330)
Hepatitis B	216 (181 to 256)	6 (2 to 11)	0 (0 to 0)	0 (0 to 0)	53 (36 to 70)	36 (27 to 50)	310 (260 to 368)
Measles	7148 (536 to 38 127)	1505 (441 to 4766)	86 (−8 to 265)	329 (−33 to 1346)	7013 (1997 to 16 990)	158 (−5 to 951)	16 239 (4187 to 57 331)
Meningitis A	0 (0 to 0)	..	..	..	..	..	0 (0 to 0)
Rubella	2 (−52 to 45)	−4 (−322 to 237)	21 (−131 to 215)	−18 (−168 to 18)	9 (−19 to 44)	0 (−15 to 16)	10 (−343 to 274)
Yellow fever	1513 (369 to 3244)	0 (0 to 0)	..	−17 (−79 to 28)	..	..	1496 (325 to 3231)
Total	9022 (1706 to 39 882)	1508 (332 to 4723)	108 (−60 to 371)	317 (−105 to 1360)	7170 (2182 to 17 178)	197 (35 to 998)	18 321 (6246 to 58 522)

Data are n (95% CrI). Meningitis A was run for five countries only. There are 42 VIMC countries in the WHO African region, 14 VIMC countries in the Eastern Mediterranean region, 15 VIMC countries in the European region, 15 VIMC countries in the region of the Americas, 10 VIMC countries in the South-East Asia region, and 16 VIMC countries in the Western Pacific region. CrI=credible interval. HPV=human papillomavirus. VIMC=Vaccine Impact Modelling Consortium.

**Figure 3:**
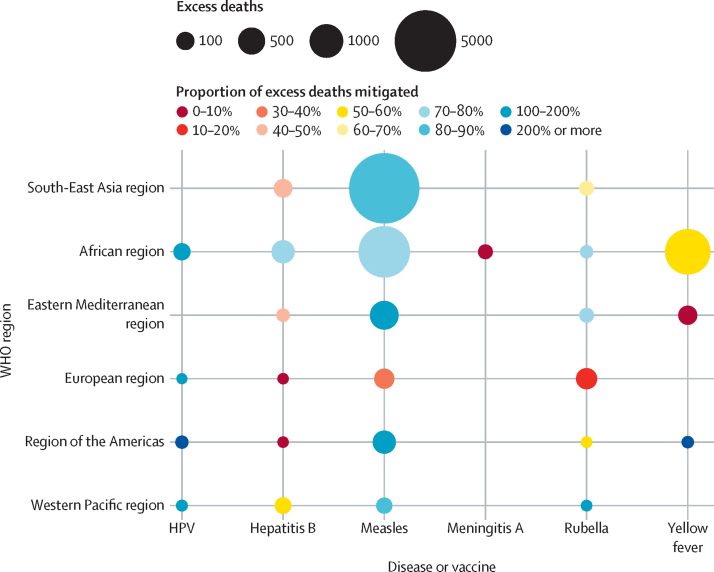
Excess deaths due to vaccine-coverage disruption and proportion of those deaths mitigated by catch-up activities (calendar years 2020–30) HPV=human papillomavirus.

These deaths averted represent 78·9% of excess deaths (95% CrI 40·4–151·4) between calendar years 2023 and 2030, after the first catch-up activity had taken place (ie, 18 900 [7037–60 223] of 25 356 [9859–75 073]). Therefore, the majority of excess deaths due to disruption from calendar year 2023 until 2030 could be mitigated by catch-up vaccination activities (figure 3). The largest excess deaths for calendar years 2023–30 were for measles; however, 70–100% of these excess deaths could be mitigated in the South-East Asia, Eastern Mediterranean, and African regions. By contrast, for yellow fever, only 50–60% of deaths could be averted in the African region for these years. In some cases, particularly for HPV, the number of mitigated deaths exceeded the number of excess deaths; this can occur when the catch-up activities have longer and more positive effect than the corresponding disruption.

**Figure 4:**
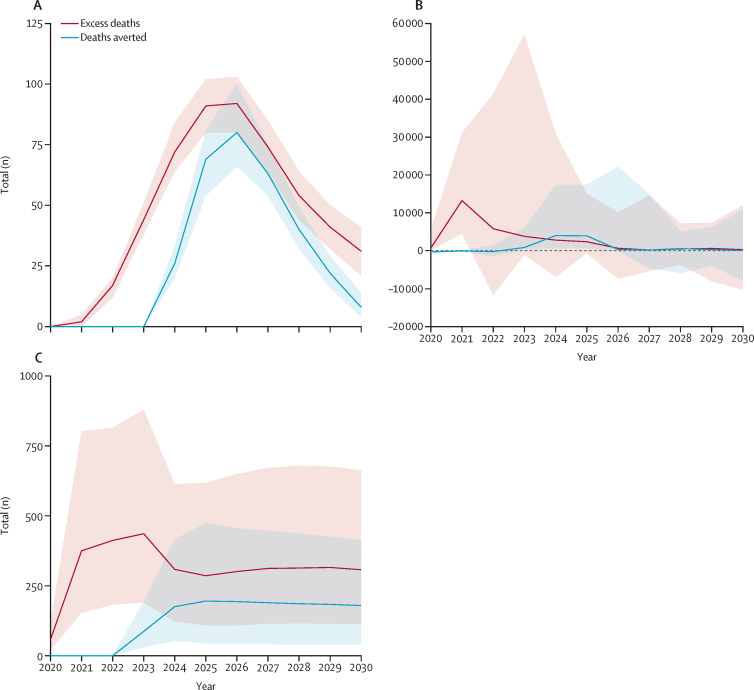
Additional deaths due to vaccine-coverage disruption and deaths averted by catch-up activities (calendar years 2020–30) (A) Hepatitis B. (B) Measles. (C) Yellow fever. 50%, 85%, and 95% CIs are shown by ribbons; median estimates are shown by solid lines. Diseases for which the median excess burden did not exceed 50 in any one year are not shown. Scales are variable between diseases.

The timing of the deaths averted by catch-up activities is important in comparison with excess burden due to disruption (figure 4). For example, additional burden for yellow fever and measles occurred shortly after disruption.

## Discussion

We estimated that disruption to measles, rubella, HPV, hepatitis B, and yellow fever vaccination could lead to approximately 49 000 additional deaths during calendar years 2020–30. This finding was mainly driven by excess measles burden, which accounted for approximately 44 500 deaths. These estimates are the first of the effects of COVID-19-pandemic-related coverage disruption on vaccine effects and implications for mitigation of excess burden since estimates of coverage declines were published.^4^ When we considered the long-term effects by year of vaccination for 14 vaccines and 112 countries, we estimated that disruption would lead to a 2·7% reduction in overall effect for years of vaccination 2020–30 or an increase of approximately 967 000 deaths. Mitigation through catch-up activities in the form of intensified routine immunisation could be effective, although the timing of when burden was averted varied. We estimated that catch-up activities could avert approximately 79% of excess deaths between calendar years 2023 and 2030 for measles, rubella, HPV, hepatitis B, and yellow fever. Although meningitis A was modelled for a subset of countries, these results were likely to be representative of all endemic countries as the subset were chosen due to high burden or disruption of immunisation campaigns. Although meningitis A was modelled for a subset of endemic countries, these countries were all high-burden countries before vaccine introduction and likely to show the worst effects of disruption.

Our results emphasise the importance of timely catch-up activities and interventions to address affected vaccine cohorts. We estimated that excess burden for measles and yellow fever occurred swiftly after disruption, but immunisation activities for mitigation were effective for both. Therefore, they are good candidates for catch-up activities. Continued global, concerted efforts and strong political commitment will continue to be essential to overcoming existing challenges and increasing health-care resilience. In the future, the large effect ratios for HPV vaccine highlight its importance for reducing mortality during an extended period of time; efforts to improve coverage among people who can be affected by HPV are warranted.^16^ Geographically, our results highlight the potential excess burden and opportunity for mitigation in the WHO African and South-East Asia regions, especially for measles.

Our estimate of 3·7 million deaths averted per year differs from that of Carter and colleagues^20^ as we do not include BCG vaccination, examine 112 countries rather than 194, and have more conservative targets by 2030. However, the uncertainty bounds overlap. We also do not include increased mortality due to disruption to polio vaccination activities. Similar work examining poliovirus found that short-term disruption would not necessarily affect long-term goals of eradication but might affect supply chains.^21^ However, this study included more optimistic assumptions around recovery of vaccination efforts than our Article.

Our vaccine-coverage estimates were developed with the latest available data and expert consultation for routine immunisation and SIAs. We estimated that recovery to 2019 magnitude of immunisation coverage would be achieved by 2025; we also estimated campaign frequency and catch-up activities through to 2030. We assumed recovery is non-linear, with the fastest improvement projected for 2023.^13^ However, as we projected coverage using the same algorithm for all 112 countries, there will be instances for which this assumption does not hold. Similarly, we estimated campaign frequency given expert guidance, but we accept that actual campaign frequency might be different; we did not include increased effort or prioritisation after the coverage disruption in the baseline recovery scenario. Finally, we estimated staged catch-up activities that targeted affected vaccine cohorts, as defined by people missed due to coverage disruption, from 2023 to 2025. Again, this timing is unlikely to be accurate for all vaccines or country settings but could be used as an example. Finally, these estimates do not consider the issue of double counting, as previous studies have found this is relatively low.^16^

This study had some limitations. We did not include disruption in transmission due to non-pharmaceutical interventions in the models used in this analysis. The effects of non-pharmaceutical interventions on current and future transmission is still unclear; studies on infectious diseases, including respiratory syncytial virus, have suggested short-term reductions in transmission could be followed by larger outbreaks in future.^22^, ^23^ Additionally, as there has been variation in the implementation of non-pharmaceutical interventions, the effects on our estimates will differ by country.^24^ Notably, China did not see a decrease in measles incidence related to non-pharmaceutical interventions, probably due to residually high vaccination coverage, a result that could contrast with other countries considered.^23^ Some modelling studies have examined the implications of reductions in transmission and found that they could reduce vaccine effects as non-pharmaceutical interventions avert some burden, but that service disruption outweighed any positive effects of reduced transmission.^3^ Furthermore, there are ongoing implications for existing and future social, political, and economic disruptions that could further disrupt immunisation or health systems.

Throughout the COVID-19 pandemic, there have been disruptions to essential health services, including immunisation. There have been efforts to minimise disruption, especially given early WHO guidance in 2020.^25^ Yet, disruption and interruption of services will have led to missed or affected vaccine cohorts and contributed to areas of low immunisation coverage. These areas, as well as having existing vaccine inequality, contribute to increased vaccine-preventable disease burden.^26^, ^27^ We quantified the effects of vaccine-coverage disruption and the effects of some potential mitigation measures on burden during 2020–30. The timing of catch-up activities is crucial; however, increased immunisation efforts have the potential to mitigate losses.

## Data sharing

All inputs and outputs of figures and tables are currently available at https://github.com/vimc/paper-covid-immunisation-disruptions.

## Declaration of interests

A-MH, XL, SE-L, JR, KA, MA, MJdV, MJF, KF, HF, TH, MJ, AK, SMM, SN, TP, TAP, AP, QTM, EV, AKW, HB, CC, HEC, AD, SH, JH, KJ, CK, J-HK, JK, BAL, VEP, YT, KW, NMF, CLT, and KAMG received funding from Gavi, the Vaccine Alliance and the Bill & Melinda Gates Foundation via the Vaccine Impact Modelling Consortium (VIMC) during the study. A-MH, JR, SE-L, XL, SN, MJdV, TH, WH, KW, NMF, CLT, and KAMG receive funding from the Medical Research Council Centre for Global Infectious Disease Analysis (reference MR/R015600/1), which is jointly funded by the UK Medical Research Council and the UK Foreign, Commonwealth, and Development Office under a concordant agreement, and is also part of the European and Developing Countries Clinical Trials Partnership programme supported by the EU. A-MH, JR, SE-L, XL, SN, MJdV, TH, WH, KW, NMF, CLT, and KAMG receive funding from Community Jameel. A-MH is supported by the German Federal Ministry of Education and Research (grant 01LN2210A) and declares stock options in BIONTECH. CLT received payment for advice from GlaxoSmithKline. KA is supported by the Japan Agency for Medical Research and Development (JP223fa627004). KAMG received a speaker fee from Sanofi Pasteur. SN receives consulting fees from WHO. VEP is a member of the WHO Immunization and Implementation Research Advisory Committee. BAL receives personal fees from Epidemiologic Research and Methods and Hillevax. SMM receives consultant fees from Emergent Biosolutions. NMF receives grant funding from Janssen Pharmaceuticals, UK Research and Innovation, and the UK National Institute for Health and Care Research; declares consulting fees from the World Bank, WHO, and Gavi; receives travel expenses for WHO meetings; was on an advisory board for Takeda; and is a senior editor for *eLife*. All other authors declare no competing interests.

## Supplementary Materials

Arabic translation of the abstract

Chinese translation of the abstract

French translation of the abstract

Portguese translation of the abstract

Spanish translation of the abstract

## References

[1] Cardoso Pinto AM, Ranasinghe L, Dodd PJ, Budhathoki SS, Seddon JA, Whittaker E (2022). Disruptions to routine childhood vaccinations in low- and middle-income countries during the COVID-19 pandemic: a systematic review. Front Pediatr.

[2] Muhoza P, Danovaro-Holliday MC, Diallo MS (2021). Routine vaccination coverage—worldwide, 2020. MMWR Morb Mortal Wkly Rep.

[3] Mburu CN, Ojal J, Chebet R (2021). The importance of supplementary immunisation activities to prevent measles outbreaks during the COVID-19 pandemic in Kenya. BMC Med.

[4] WHO (2022). COVID-19 pandemic fuels largest continued backslide in vaccinations in three decades. https://www.who.int/news/item/15-07-2022-covid-19-pandemic-fuels-largest-continued-backslide-in-vaccinations-in-three-decades.

[5] Minta AA, Ferrari M, Antoni S (2023). Progress toward regional measles elimination—worldwide, 2000–2021. https://www.scienceopen.com/document?vid=04d5519e-d291-4871-be08-5ec2241bafa0.

[6] WHO (2023). WHO/UNICEF coverage estimates of national immunization coverage. https://www.who.int/teams/immunization-vaccines-and-biologicals/immunization-analysis-and-insights/global-monitoring/immunization-coverage/who-unicef-estimates-of-national-immunization-coverage.

[7] Shet A, Carr K, Danovaro-Holliday MC (2022). Impact of the SARS-CoV-2 pandemic on routine immunisation services: evidence of disruption and recovery from 170 countries and territories. Lancet Glob Health.

[8] Hartner A-M, Li X, Gaythorpe KAM (2023). COVID-19-related disruption and resiliency in immunisation activities in LMICs: a rapid review. medRxiv.

[9] Strategic Advisory Group of Experts on Immunization (2018). 2018 Assessment report of the Global Vaccine Action Plan. https://www.who.int/publications/i/item/2018-assessment-report-of-the-global-vaccine-action-plan.

[10] Ali HA, Hartner A-M, Echeverria-Londono S (2022). Vaccine equity in low and middle income countries: a systematic review and meta-analysis. Int J Equity Health.

[11] UNICEF (2023). The state of the world's children 2023. https://www.unicef.org/reports/state-worlds-children-2023.

[12] Wiegand M, Eagan RL, Karimov R, Lin L, Larson HJ, de Figueiredo A (2023). Global declines in vaccine confidence from 2015 to 2022: a large-scale retrospective analysis. SSRN.

[13] O'Brien K (2023). Member states information session—the global immunization “Big Catch-Up” effort. https://apps.who.int/gb/MSPI/pdf_files/2023/03/Item1_24-03.pdf.

[14] WHO (2021). Leave no one behind: guidance for planning and implementing catch-up vaccination. https://iris.who.int/bitstream/handle/10665/340749/9789240016514-eng.pdf?sequence=1.

[15] WHO (2021). Immunization repository. https://www.who-immunization-repository.org/dhisweb-commons/security/login.action.

[16] Toor J, Echeverria-Londono S, Li X (2021). Lives saved with vaccination for 10 pathogens across 112 countries in a pre-COVID-19 world. Elife.

[17] Evans B, Jombart T (2022). Worldwide routine immunisation coverage regressed during the first year of the COVID-19 pandemic. Vaccine.

[18] Echeverria-Londono S, Li X, Toor J (2021). How can the public health impact of vaccination be estimated?. BMC Public Health.

[19] Carter ED, Zimmerman L, Qian J, Roberton T, Seme A, Shiferaw S (2022). Impact of the early stages of the COVID-19 pandemic on coverage of reproductive, maternal, and newborn health interventions in Ethiopia: a natural experiment. Front Public Health.

[20] Carter A, Msemburi W, Sim SY (2023). Modeling the impact of vaccination for the immunization agenda 2030: deaths averted due to vaccination against 14 pathogens in 194 countries from 2021–2030. Vaccine.

[21] Kalkowska DA, Voorman A, Pallansch MA (2023). The impact of disruptions caused by the COVID-19 pandemic on global polio eradication. Vaccine.

[22] Baker RE, Park SW, Yang W, Vecchi GA, Metcalf CJE, Grenfell BT (2020). The impact of COVID-19 nonpharmaceutical interventions on the future dynamics of endemic infections. Proc Natl Acad Sci USA.

[23] Geng MJ, Zhang HY, Yu LJ (2021). Changes in notifiable infectious disease incidence in China during the COVID-19 pandemic. Nat Commun.

[24] Hale T, Angrist N, Kira B, Petherick A, Phillips T, Webster S (2020). Variation in government responses to COVID-19. https://www.bsg.ox.ac.uk/sites/default/files/2020-05/BSG-WP-2020-032-v6.0.pdf.

[25] Shet A, Dhaliwal B, Banerjee P (2021). COVID-19-related disruptions to routine vaccination services in India: a survey of paediatric providers. BMJ Paediatr Open.

[26] Hogan D, Gupta A (2023). Why reaching zero-dose children holds the key to achieving the sustainable development goals. Vaccines (Basel).

[27] Wigley A, Lorin J, Hogan D (2022). Estimates of the number and distribution of zero-dose and under-immunised children across remote-rural, urban, and conflict-affected settings in low and middle-income countries. PLoS Glob Public Health.

