# Supplementary material for: Estimating the health effects of COVID-19-related immunisation disruptions in 112 countries during 2020–30: a modelling study
Source: Lancet Glob Health. 2024 Mar 12;12(4):e563–71. doi: 10.1016/S2214-109X(23)00603-4 (PMC10951961; doi:10.1016/S2214-109X(23)00603-4)
Supplement: Arabic translation of the abstract [file mmc1.pdf]

THE LANCET
Global Health

Supplementary appendix 1

This translation in Arabic was submitted by the authors and we reproduce it as supplied. It has not been peer reviewed. *The Lancet*'s editorial processes have only been applied to the original in English, which should serve as reference for this manuscript.

تم تقديم هذه الترجمة باللغة العربية من قبل المؤلفين ونعيد إنتاجها كما هو مُقدم. إنها لم تخضع لاستعراض الأقران. تم تطبيق عمليات تحرير لانسيت فقط على النص الأصلي باللغة الإنجليزية، والذي يجب أن يكون بمثابة مرجع لهذه المخطوطة.

Supplement to: Hartner A-M, Li X, Echeverria-Londono S, et al. Estimating the health effects of COVID-19-related immunisation disruptions in 112 countries during 2020–30: a modelling study. *Lancet Glob Health* 2024; **12:** e563–71.

تقدير الآثار الصحية لتعطل التحصين ضد كوفيد-19 في 112 بلدًا خلال الفترة من 2020 إلى 2030: دراسة نمذجة

ملخص

معلومات أساسية: حدثت انخفاضات في تغطية التحصين على مستوى العالم بسبب جائحة كوفيد-19. وقد بدأت عملية التعافي من آثارها ولكن بدرجة تختلف حسب الموقع الجغرافي. أدى هذا التعطل إلى انخفاض معدل تحصين المجموعات وتوقف التقدم الذي تم إحرازه على صعيد الحد من عبء المرض الذي يمكن الوقاية منه عن طريق اللقاحات. ظهرت، حتى الآن، دراسات قليلة تتناول آثار تعطل التغطية على قدرة اللقاح على الوقاية من المرض. كان الهدف من الدراسة هو تحديد آثار تعطل تغطية اللقاحات على خدمات وحملات التحصين الدورية، وتحديد المجموعات والمناطق التي يمكن أن تستفيد بشكل خاص من الأنشطة الرامية لتدارك الأمر، وتحديد ما إذا كان يمكن التعافي من آثار الخسائر التي تحققت أم لا.

الأساليب المتبعة: لأغراض دراسة النمذجة الموجودة بين أيدينا، استخدمنا مجموعات نمذجة من "اتحاد نمذجة تأثير اللقاحات" من 112 بلدًا منخفضة ومتوسطة الدخل لتقدير تأثير اللقاح على 14 مسببًا من مسببات الأمراض. استخدمت مجموعة واحدة من تقديرات النمذجة بيانات تغطية اللقاحات خلال الفترة من عام 1937 إلى عام 2021 لمجموعة فرعية من الأمراض التي يمكن الوقاية منها باللقاحات أو المعرضة للتفشي أو ذات الأولوية (مثل الحصبة والحصبة الألمانية والتهاب الكبد B وفيروس الورم الحليمي البشري [HPV] والتهاب السحايا A والحمى الصفراء) للتعرف على تدابير التخفيف من الآثار، والتي يُشار إليها فيما يلي باسم "أشواط التعافي". تم إجراء المجموعة الثانية من التقديرات باستخدام بيانات تغطية اللقاحات خلال الفترة من عام 1937 إلى عام 2020 وذلك لحساب نسب التأثير (أي العبء الذي تم تجنبه لكل جرعة) لجميع اللقاحات والأمراض الأربعة عشر المدرجة، والتي يُشار إليها فيما يلي باسم "الأشواط الكاملة". تمت نمذجة كلا النوعين من الأشواط من 1 يناير 2000 وحتى 31 ديسمبر 2100. تم إدراج البلدان في الدراسة إذا كانت عضوًا في "التحالف العالمي لتوفير اللقاحات والتحصين"، أو مجموعة مشروعات تحالف اللقاحات، أو كانت تعاني عبئًا ملحوظًا للمرض، أو قامت بأنشطة تطعيم استراتيجية ملحوظة. وتمثل هذه البلدان غالبية العبء العالمي للأمراض التي يمكن الوقاية منها باللقاحات. استندت تغطية اللقاحات إلى التقديرات التاريخية المستمدة من تقديرات منظمة الصحة العالمية واليونيسف للتغطية الوطنية بالتحصين ومستودع التحصين التابع لمنظمة الصحة العالمية للحصول على البيانات حتى عام 2021. واعتبارًا من عام 2022 فصاعدًا، قدّرنا التغطية بناءً على الإرشادات المتعلقة بتواتر الحملة، والافتراضات غير الخطية بشأن التعافي الناجم عن التحصين الروتيني إلى مستوى ما قبل التعطل، ونقاط نهاية عام 2030 المستندة إلى أهداف خطة التحصين لعام 2030 لمنظمة الصحة العالمية ومشورة الخبراء. درسنا ثلاثة سيناريوهات رئيسية: عدم حدوث تعطل، والتعافي الأساسي، والتعافي الأساسي والتدارك.

النتائج: قدّرنا أن تعطل لقاحات الحصبة والحصبة الألمانية وفيروس الورم الحليمي البشري والتهاب الكبد B والتهاب السحايا A والحمى الصفراء يمكن أن يؤدي إلى 49,119 حالة وفاة إضافية (95٪ منها خلال فترة زمنية موثوق بها من 17,248 إلى 134,941) خلال السنوات من 2020 إلى 2030، ويرجع ذلك إلى حد كبير إلى الحصبة. بالنسبة إلى سنوات التطعيم من 2020 إلى 2030 لجميع مسببات الأمراض الأربعة عشر، يمكن أن يؤدي التعطل إلى انخفاض بنسبة 2.66٪ (95٪ منها خلال فترة زمنية موثوق بها من 2.52 إلى 2.81) في التأثير طويل المدى من 37,378,194 حالة وفاة تم تجنبها (من 34,450,249 إلى 40,241,202) إلى 36,410,559 حالة وفاة تم تجنبها (من 33,515,397 - 39,241,799). قدّرنا أن أنشطة التدارك يمكن أن تتجنب 78.9٪ (من 40.4 إلى 151.4) من حالات الوفاة الزائدة في السنوات من 2023 إلى 2030 (أي 18,900 [7,037 إلى 60,223] من 25,356 [9,859 إلى 75,073]).

التفسير: تسلط نتائجنا الضوء على مدى أهمية توقيت الأنشطة التداركية، مع الوضع في الاعتبار العبء المقدر لتحسين تغطية اللقاح في المجموعات المتضررة. وتشير تقديراتنا إلى أن تدابير التخفيف من الحصبة والحمى الصفراء التي تم تطبيقها كانت فعالة بشكل خاص في الحد من العبء الزائد على المدى القصير. وعلاوة على ذلك، يستدعي التأثير الكبير طويل المدى للقاح فيروس الورم الحليمي البشري كأداة مهمة للوقاية من سرطان عنق الرحم استمرار جهود التحصين بعد التعطل.

التمويل: اتحاد نمذجة تأثير اللقاحات بتمويل من التحالف العالمي لتوفير اللقاحات والتحصين وتحالف اللقاحات ومؤسسة بيل وميليندا جيتس.

حقوق النشر © لعام 2024 مملوكة للمؤلف (للمؤلفين). تم نشر هذه الدراسة بواسطة Elsevier Ltd. تخضع هذه المقالة لشروط "التداول الحر" بموجب ترخيص CC BY 4.0.
