# Supplementary material for: Estimating the health effects of COVID-19-related immunisation disruptions in 112 countries during 2020–30: a modelling study
Source: Lancet Glob Health. 2024 Mar 12;12(4):e563–71. doi: 10.1016/S2214-109X(23)00603-4 (PMC10951961; doi:10.1016/S2214-109X(23)00603-4)
Supplement: Chinese translation of the abstract [file mmc2.pdf]

THE LANCET
Global Health

Supplementary appendix 2

This translation in Chinese was submitted by the authors and we reproduce it as supplied. It has not been peer reviewed. *The Lancet*'s editorial processes have only been applied to the original in English, which should serve as reference for this manuscript.

此简体中文译文由作者提交，我方按照提供的版本刊登。此译文并未经过同行审阅。医学期刊《柳叶刀》的编辑流程仅适用于英文原稿，英文原稿应作为此手稿的参考。

Supplement to: Hartner A-M, Li X, Echeverria-Londono S, et al. Estimating the health effects of COVID-19-related immunisation disruptions in 112 countries during 2020–30: a modelling study. *Lancet Glob Health* 2024; **12:** e563–71.

估计 2020-2030 年期间 112 个国家/地区因新冠疫情致免疫接种中断对健康的影响：一项建模研究

概要

背景信息：由于新冠疫情，全球免疫接种覆盖率有所下降。现免疫接种已逐步恢复，但恢复程度因各国家／地区而异。中断导致群体免疫接种不足，并妨碍了减少疫苗可预防疾病负担的进展。迄今为止，关于覆盖中断对疫苗有效性影响的研究不多。我们旨在量化疫苗覆盖中断对常规和强化免疫服务的影响，确定特别可能从补种活动中受益的群体和地区，并确定是否可以弥补实际损失。

方法：在这项建模研究中，疫苗影响建模联盟（VIMC）的建模团队估计了疫苗在112个低收入和中等收入国家对14种病原体的影响。第一组建模估计使用 1937 年至 2021 年的疫苗覆盖率数据，针对的是疫苗可预防、易暴发或重点疾病的子集（如麻疹、风疹、乙型肝炎、人瘤病毒 [HPV]、甲型脑膜炎和黄热病），以研究缓解措施，以下简称"恢复型运行"。第二组估计是使用 1937 年至 2020 年的疫苗覆盖率数据进行的，用于计算所有 14 种纳入的疫苗和疾病（以下简称"全面型运行"）的效应比（即每剂疫苗避免的负担）。两次模型运行皆从 2000 年 1 月 1 日到 2100 年 12 月 31 日建模。受全球疫苗免疫联盟（Gavi）支持、有显着的相关疾病负担、或有显着的疫苗接种活动策略的国家和地区被纳入建模研究。这些国家在全球疫苗可预防疾病负担中占大多数比例。疫苗覆盖率是根据世卫组织-联合国儿童基金会（WHO-UNICEF）国家免疫覆盖率估计和世卫组织（WHO）免疫资料库对截至 2021 年（含）的数据进行的历史估计得出的。从 2022 年起，我们根据关于强化免疫活动频率的指导、关于常规免疫恢复到中断前规模的非线性假设来估计疫苗覆盖率，另外对于2030年的疫苗覆盖率目标的估计来源于世卫组织《2030 年免疫议程》（WHO Immunization Agenda 2030）和专家咨询。我们通过建模研究了三种主要情况：无中断、基线恢复以及基线恢复和补种。

调查结果：我们估计，在 2020-2030 日历年期间，麻疹、风疹、HPV、乙型肝炎、甲型脑膜炎和黄热病疫苗接种的中断可能导致 49,119 例超额死亡（95% 可信区间[CrI] 17,248-134,941例），主要是由于麻疹。在 2020-2030 年对所有 14 种病原体进行疫苗接种的年份中，疫苗接种中断可能导致疫苗长期影响减少 2.66%（95% CrI 2.52-2.81%），从避免的 37,378,194 例死亡（34,450,249 – 40,241,202 例）减至 36,410,559 例避免的死亡（33,515,397 – 39,241,799例）。我们估计，在 2023 年至 2030 年期间，疫苗补种活动可以避免 78.9%（40.4-151.4%）的超额死亡（即 25,356 例 [9,859-75,073] 中的 18,900 例 [7,037-60,223]）。

解释：考虑到提高受影响群体疫苗覆盖率相关的疾病负担的估计，我们的研究结果强调了补种活动时机的重要性。我们估计，麻疹和黄热病的缓解措施在短期内对减轻超额疾病负担尤为有效。此外，HPV 疫苗作为一种重要的宫颈癌预防工具，具有出色的长期效果，因此免疫接种工作有必要在中断后继续进行。

资金：疫苗影响建模联盟由全球疫苗免疫联盟和比尔及梅琳达·盖茨基金会资助。

版权 © 2024 作者。由 Elsevier Ltd. 出版。这是一篇根据 CC BY 4.0 许可条款发布的一篇开放获取文章。
