# Supplementary material for: Estimating the health effects of COVID-19-related immunisation disruptions in 112 countries during 2020–30: a modelling study
Source: Lancet Glob Health. 2024 Mar 12;12(4):e563–71. doi: 10.1016/S2214-109X(23)00603-4 (PMC10951961; doi:10.1016/S2214-109X(23)00603-4)
Supplement: French translation of the abstract [file mmc3.pdf]

THE LANCET
Global Health

Supplementary appendix 3

This translation in French was submitted by the authors and we reproduce it as supplied. It has not been peer reviewed. *The Lancet*'s editorial processes have only been applied to the original in English, which should serve as reference for this manuscript.

Cette traduction en français a été proposée par les auteurs et nous l'avons reproduite telle quelle. Elle n'a pas été examinée par des pairs. Les processus éditoriaux du *Lancet* n'ont été appliqués qu'à l'original en anglais et c'est cette version qui doit servir de référence pour ce manuscrit.

Supplement to: Hartner A-M, Li X, Echeverria-Londono S, et al. Estimating the health effects of COVID-19-related immunisation disruptions in 112 countries during 2020–30: a modelling study. *Lancet Glob Health* 2024; **12:** e563–71.

Estimation des effets des perturbations de la vaccination liées à la COVID-19 sur la santé dans 112 pays au cours de la période 2020–30 : une étude de modélisation

Résumé

Contexte : La pandémie de COVID-19 a entraîné une baisse de la couverture vaccinale dans le monde. La reprise a commencé, maisest géographiquement variable.. Cette perturbation a conduit à des cohortes sous-vaccinées et a interrompu les progrès dans la réduction du fardeau des maladies évitables par la vaccination. Jusqu'à présent, peu d'études ont été menées sur les incidences de la perturbation de la couverture vaccinale sur les effets des vaccins. Notre objectif était de quantifier les effets de la perturbation de la couverture vaccinale sur les services de vaccination systématique et dans le cadre des campagnes de vaccination, d'identifier les cohortes et les régions qui pourraient bénéficier tout particulièrement d'activités de rattrapage et d'établir si les pertes dans effet pouvaient être récupérées.

Méthodes : Pour cette étude de modélisation, nous avons utilisé des groupes de modélisation du Vaccine Impact Modelling Consortium provenant de 112 pays à faible revenu et à revenu intermédiaire pour estimer l'effet des vaccins pour 14 agents pathogènes. Une série d'estimations de modélisation a utilisé les données de couverture vaccinale de 1937 à 2021 pour un sous-ensemble de maladies évitables par la vaccination, sujettes à des épidémies ou prioritaires (rougeole, rubéole, hépatite B, virus du papillome humain [VPH], méningite A et fièvre jaune) afin d'examiner les mesures d'atténuation, ci-après dénommées « séries de récupération ». La deuxième série d'estimations a été réalisée à partir des données de couverture vaccinale de 1937 à 2020, utilisées pour calculer les ratios d'effet (c'est-à-dire la charge évitée par dose) pour l'ensemble des 14 vaccins et maladies inclus, ci-après dénommés « séries complètes ». Les deux séries ont été modélisées du 1er janvier 2000 au 31 décembre 2100. Les pays ont été inclus s'ils faisaient partie du portefeuille de Gavi, l'Alliance du Vaccin, s'ils avaient une charge de morbidité notable ou s'ils avaient des activités stratégiques notables en matière de vaccination. Ces pays représentaient la majorité de la charge de morbidité mondiale due aux maladies évitables par la vaccination. La couverture vaccinale a été déterminée à partir des estimations historiques des estimations de la couverture vaccinale nationale de l'OMS-UNICEF et du répertoire des vaccinations de l'OMS pour les données allant jusqu'à 2021 inclus. À partir de 2022, nous avons estimé la couverture sur la base d'orientations concernant la fréquence des campagnes, d'hypothèses non linéaires concernant le rétablissement de la vaccination systématique à son niveau d'avant la perturbation, et de critères d'évaluation pour 2030 fondés sur les objectifs du Programme pour la vaccination à l'horizon 2030 de l'OMS et sur la consultation d'experts. Nous avons examiné trois scénarios principaux : pas de perturbation, rétablissement de la situation de base, rétablissement de la situation de base et rattrapage.

Conclusions : Nous avons estimé que l'interruption de la vaccination contre la rougeole, la rubéole, le VPH, l'hépatite B, la méningite A et la fièvre jaune pourrait entraîner 49 119 décès supplémentaires (intervalle crédible à 95 % 17 248 – 134 941) au cours des années civiles 2020–30, en grande partie à cause de la rougeole. Pour les années de vaccination 2020–30 pour les 14 agents pathogènes, la perturbation pourrait entraîner une réduction de 2,66 % (intervalle crédible à 95 % : 2,52 – 2,81) de l'effet à long terme, qui passerait de 37 378 194 décès évités (34 450 249 – 40 241 202) à 36 410 559 décès évités (33 515 397 – 39 241 799). Nous avons estimé que les activités de rattrapage pourraient permettre d'éviter 78,9 % (40,4 – 151,4) des décès en excès entre les années civiles 2023 et 2030 (soit 18 900 [7 037 – 60 223] sur 25 356 [9 859 – 75 073]).

Interprétation : Nos résultats soulignent l'importance du calendrier des activités de rattrapage, compte tenu de la charge estimée pour améliorer la couverture vaccinale dans les cohortes touchées. Nous avons estimé que les mesures d'atténuation pour la rougeole et la fièvre jaune étaient particulièrement efficaces pour réduire la charge excessive à court terme. En outre, l'effet à long terme élevé du vaccin contre le VPH en tant qu'outil important de prévention du cancer du col de l'utérus justifie la poursuite des efforts de vaccination après la perturbation.

Financement : le Vaccine Impact Modelling Consortium, financé par Gavi, l'Alliance du Vaccin et la Fondation Bill et Melinda Gates.

Droits d'auteur © 2024 L'auteur/les auteurs. Publié par Elsevier Ltd. Il s'agit d'un article en libre accès sous licence CC BY 4.0.
