# Supplementary material for: Estimating the health effects of COVID-19-related immunisation disruptions in 112 countries during 2020–30: a modelling study
Source: Lancet Glob Health. 2024 Mar 12;12(4):e563–71. doi: 10.1016/S2214-109X(23)00603-4 (PMC10951961; doi:10.1016/S2214-109X(23)00603-4)
Supplement: Portguese translation of the abstract [file mmc4.pdf]

THE LANCET
Global Health

Supplementary appendix 4

This translation in Portuguese was submitted by the authors and we reproduce it as supplied. It has not been peer reviewed. *The Lancet*'s editorial processes have only been applied to the original in English, which should serve as reference for this manuscript.

Esta tradução em português foi submetida pelos autores e nós não fizemos quaisquer alterações. Esta versão não foi revista por pares. O processo editorial do The Lancet só foi aplicado à versão original em inglês, que deve servir como referência para este artigo.

Supplement to: Hartner A-M, Li X, Echeverria-Londono S, et al. Estimating the health effects of COVID-19-related immunisation disruptions in 112 countries during 2020–30: a modelling study. *Lancet Glob Health* 2024; **12:** e563–71.

Estimativa dos efeitos na saúde das interrupções da vacinação relacionadas com a COVID-19 em 112 países durante 2020-2030: um estudo de modelagem

Resumo

Contexto: A cobertura global da vacinação diminuiu devido à pandemia de COVID-19. A recuperação já começou, mas é geograficamente variável. Esta perturbação deu origem a coortes subimunizadas e interrompeu os progressos na redução do peso das doenças evitáveis por vacinação. Até à data, foram efetuados poucos estudos sobre os efeitos da perturbação da cobertura nos efeitos das vacinas. O nosso objetivo era quantificar os efeitos da perturbação da cobertura vacinal nos serviços de imunização de rotina e de campanha, identificar coortes e regiões que pudessem beneficiar particularmente de atividades de recuperação e determinar se as perdas de efeito poderiam ser recuperadas.

Métodos: Para este estudo de modelagem, utilizámos grupos de modelagem do Vaccine Impact Modelling Consortium de 112 países de baixo e médio rendimento para estimar o efeito da vacina para 14 agentes patogénicos. Um conjunto de estimativas de modelagem utilizou dados de cobertura vacinal de 1937 a 2021 para um subconjunto de doenças preveníveis por vacinação, propensas a surtos ou prioritárias (ou seja, sarampo, rubéola, hepatite B, papilomavírus humano [HPV], meningite A e febre-amarela) para examinar as medidas de mitigação, a seguir designadas por ciclos de recuperação. O segundo conjunto de estimativas foi realizado com dados de cobertura vacinal de 1937 a 2020, utilizados para calcular os rácios de efeito (ou seja, o fardo evitado por dose) para todas as 14 vacinas e doenças incluídas, a seguir designadas por séries completas. Ambas as séries foram modeladas de 1 de janeiro de 2000 a 31 de dezembro de 2100. Os países foram incluídos caso: pertencessem ao portefólio da Gavi, a Aliança para as Vacinas; tivessem um fardo considerável; ou tivessem atividades de vacinação estratégica notáveis. Estes países representavam a maioria do peso global das doenças evitáveis por vacinação. A cobertura vacinal foi informada por estimativas históricas das Estimativas de Cobertura Nacional de Vacinação da OMS-UNICEF e do repositório de imunização da OMS para dados até 2021, inclusive. A partir de 2022, estimámos a cobertura com base em orientações sobre a frequência das campanhas, pressupostos não lineares sobre a recuperação da imunização de rotina para a magnitude anterior à perturbação e pontos finais de 2030 informados pelos objetivos da Agenda de Imunização da OMS para 2030 e pela consulta de especialistas. Examinámos três cenários principais: ausência de perturbações, recuperação da base de referência e recuperação.

Conclusões: Estimámos que a interrupção da vacinação contra o sarampo, a rubéola, o HPV, a hepatite B, a meningite A e a febre-amarela poderia levar a 49 119 mortes adicionais (intervalo credível [ICr] de 95% 17 248 - 134 941) durante os anos civis de 2020-2030, em grande parte devido ao sarampo. Para os anos de vacinação 2020-2030 para todos os 14 agentes patogénicos, a perturbação poderia levar a uma redução de 2,66% (95% ICr 2,52-2,81) no efeito a longo prazo de 37 378 194 mortes evitadas (34 450 249 - 40 241 202) para 36 410 559 mortes evitadas (33 515 397 - 39 241 799). Estimamos que as atividades de recuperação poderiam evitar 78,9% (40,4-151,4) do excesso de mortes entre os anos civis de 2023 e 2030 (ou seja, 18 900 [7 037 - 60 223] de 25 356 [9 859 - 75 073]).

Interpretação: Os nossos resultados sublinham a importância da calendarização das atividades de recuperação, tendo em conta os encargos estimados para melhorar a cobertura vacinal nas coortes afetadas. Estimámos que as medidas de mitigação para o sarampo e a febre-amarela foram particularmente eficazes na redução do excesso do fardo a curto prazo. Além disso, o elevado efeito a longo prazo da vacina contra o HPV como importante ferramenta de prevenção do cancro do colo do útero justifica a continuação dos esforços de imunização após a perturbação.

Financiamento: O Vaccine Impact Modelling Consortium é financiado pela Gavi, a Aliança para as Vacinas, e pela Fundação Bill e Melinda Gates.

Direitos de autor © 2024 O(s) autor(es). Publicado por Elsevier Ltd. Este artigo está disponível em acesso livre ao abrigo da licença CC BY 4.0.
