# Supplementary material for: Estimating the health effects of COVID-19-related immunisation disruptions in 112 countries during 2020–30: a modelling study
Source: Lancet Glob Health. 2024 Mar 12;12(4):e563–71. doi: 10.1016/S2214-109X(23)00603-4 (PMC10951961; doi:10.1016/S2214-109X(23)00603-4)
Supplement: Spanish translation of the abstract [file mmc5.pdf]

THE LANCET
Global Health

Supplementary appendix 5

This translation in Spanish was submitted by the authors and we reproduce it as supplied. It has not been peer reviewed. *The Lancet*'s editorial processes have only been applied to the original in English, which should serve as reference for this manuscript.

Los autores nos proporcionaron esta traducción al español y la reproducimos tal como nos fue entregada. No la hemos revisado. Los procesos editoriales de *The Lancet* se han aplicado únicamente al original en inglés, que debe servir de referencia para este manuscrito.

Supplement to: Hartner A-M, Li X, Echeverria-Londono S, et al. Estimating the health effects of COVID-19-related immunisation disruptions in 112 countries during 2020–30: a modelling study. *Lancet Glob Health* 2024; **12:** e563–71.

Estimación de los efectos sobre la salud de las interrupciones de la inmunización relacionadas con la COVID-19 en 112 países entre 2020-2030: un estudio de modelización

Resumen

Antecedentes: Se han producido descensos en la cobertura mundial de inmunización debido a la pandemia de la COVID-19. La recuperación ha comenzado, pero es geográficamente variable. Esta alteración ha dado lugar a cohortes subinmunizadas y ha interrumpido los avances en la reducción de la carga de enfermedades prevenibles mediante vacunación. Hasta ahora se han llevado a cabo pocos estudios sobre los efectos de la interrupción de la cobertura en los efectos de las vacunas. Nuestro objetivo era cuantificar los efectos de la interrupción de la cobertura de vacunación en los servicios de inmunización sistemática y de campaña, identificar las cohortes y regiones que podrían beneficiarse especialmente de las actividades de recuperación, así como establecer si las pérdidas en vigor podrían recuperarse.

Métodos: Para este estudio de modelización, utilizamos grupos de modelización del Vaccine Impact Modelling Consortium, un consorcio de 112 países de ingresos bajos y medios, a fin de estimar el efecto de las vacunas para 14 patógenos. Un conjunto de estimaciones de modelos utilizó datos de cobertura de vacunación de 1937 a 2021 para un subconjunto de enfermedades prevenibles mediante vacuna, prioritarias o propensas a brotes (es decir, sarampión, rubeola, hepatitis B, virus del papiloma humano [VPH], meningitis A y fiebre amarilla) para estudiar las medidas de mitigación, en lo sucesivo denominadas series de recuperación. El segundo conjunto de estimaciones se desarrolló a partir de datos de cobertura de vacunación de 1937 a 2020, utilizados para calcular las relaciones de efecto (es decir, la carga evitada por dosis) para las 14 vacunas y enfermedades incluidas, en lo sucesivo denominadas series completas. Ambas series se modelizaron desde el 1 de enero de 2000 hasta el 31 de diciembre de 2100. Se incluyeron los países que formaban parte de la cartera de Gavi, la Alianza para las Vacunas, los que tenían una carga considerable o los que tenían actividades estratégicas de vacunación considerables. Estos países representaban la mayor parte de la carga mundial de enfermedades prevenibles mediante vacunación. La cobertura de vacunación se basó en estimaciones históricas de Estimates of National Immunization Coverage de la OMS-Unicef y el repositorio de inmunización de la OMS para datos hasta 2021 incluido. A partir de 2022, estimamos la cobertura basándonos en orientaciones sobre la frecuencia de las campañas, hipótesis no lineales sobre la recuperación de la inmunización sistemática hasta la magnitud anterior a la interrupción, y criterios de valoración para 2030 basados en los objetivos de la Agenda de Inmunización 2030 de la OMS y en consultas a expertos. Examinamos tres supuestos principales: sin interrupciones, recuperación de base y recuperación de base y equiparación.

Resultados: Se estimó que la interrupción de la vacunación contra el sarampión, la rubeola, el VPH, la hepatitis B, la meningitis A y la fiebre amarilla podría resultar 49 119 muertes adicionales (intervalo de credibilidad [IC] del 95 %: 17 248 – 134 941) durante los años naturales 2020-2030, en gran parte debido al sarampión. Para los años de vacunación 2020-2030 para los 14 patógenos, la interrupción podría conducir a una reducción del 2,66 % (95 % IC 2,52-2,81) en el efecto a largo plazo de 37 378 194 muertes evitadas (34 450 249-40 241 202) a 36 410 559 muertes evitadas (33 515 397-39 241 799). Se estimó que las actividades de recuperación podrían evitar el 78,9 % (40,4-151,4) del exceso de muertes entre los años naturales 2023 y 2030 (es decir, 18 900 [7037-60 223] de 25 356 [9859-75 073]).

Interpretación: Nuestros resultados destacan la importancia del calendario de las actividades de equiparación, teniendo en cuenta la carga estimada para mejorar la cobertura de vacunación en las cohortes afectadas. Estimamos que las medidas de mitigación del sarampión y la fiebre amarilla fueron especialmente eficaces para reducir el exceso de carga a corto plazo. Además, el elevado efecto a largo plazo de la vacuna contra el VPH como importante herramienta de prevención del cáncer de cuello uterino justifica la continuación de la labor de inmunización después de la interrupción.

Financiación: La financiación del Vaccine Impact Modelling Consortium proviene de Gavi, la Alianza para las Vacunas y la Fundación Bill & Melinda Gates.

Copyright © 2024 Los autores. Publicado por Elsevier Ltd. Este es un artículo de acceso abierto bajo licencia CC BY 4.0.
