# Supplementary material for: Estimating the health effects of COVID-19-related immunisation disruptions in 112 countries during 2020–30: a modelling study
Source: Lancet Glob Health. 2024 Mar 12;12(4):e563–71. doi: 10.1016/S2214-109X(23)00603-4 (PMC10951961; doi:10.1016/S2214-109X(23)00603-4)
Supplement: Supplementary appendix 6 [file mmc6.pdf]

THE LANCET
Global Health

Supplementary appendix 6

This appendix formed part of the original submission and has been peer reviewed. We post it as supplied by the authors.

Supplement to: Hartner A-M, Li X, Echeverria-Londono S, et al. Estimating the health effects of COVID-19-related immunisation disruptions in 112 countries during 2020–30: a modelling study. *Lancet Glob Health* 2024; **12:** e563–71.

Appendix

Contents

Appendix A Vaccination coverage scenarios 1
Appendix A.1 Recovery model runs completed in 2022 1
Appendix A.1.1 'No disruption' (ND) scenario 1
Appendix A.1.2 Catch-up activities 1
Appendix A.2 Full model runs completed in 2021 3
Appendix A.3 Functional forms for vaccine scale-up or recovery 5
Appendix A.3.1 Non-linear recovery to pre-pandemic (2019) levels of routine coverage 5
Appendix A.3.2 Non-linear scale-up of routine vaccination coverage towards 2030 endpoints 5
Appendix A.3.3 Defining IA2030 Endpoints 6

Appendix B Additional results 8
Appendix B.1 List of included countries 8
Appendix B.2 Excess burden due to 2020-2021 coverage disruptions 8
Appendix B.3 Mitigated burden due to catch-up activities 27
Appendix B.4 Further notes on negative estimates 33

Appendix C Vaccination coverage figures 34
Appendix C.1 Examining Coverage by Scenario 34
Appendix C.2 Examining Excess FVPs by Disease and WHO Region 34

Appendix D Model descriptions 45
Appendix D.1 Cholera – Johns Hopkins Bloomberg School of Public Health 45
Appendix D.2 Cholera and typhoid fever - International Vaccine Institute (IVI) 46
Appendix D.2.1 Cholera 47
Appendix D.2.2 Typhoid fever 48
Appendix D.3 Hib and Rotavirus – London School of Hygiene and Tropical Medicine (LSHTM) 49
Appendix D.4 Hib, PCV and Rota – Johns Hopkins University 50

Appendix D.4.1 PCV-specific assumptions 52
Appendix D.4.2 Hib vaccine-specific assumptions 53
Appendix D.4.3 Rotavirus vaccine-specific assumptions 53
Appendix D.5 HepB - Goldstein 53
Appendix D.6 HepB - Imperial 55
Appendix D.7 Human Papilloma Virus (HPV) – London School of Hygiene and Tropical Medicine (LSHTM) 56
Appendix D.8 Human Papilloma Virus (HPV) – Harvard 57
Appendix D.9 Japanese Encephalitis – National University of Singapore . . . 57
Appendix D.10 Japanese Encephalitis – University of Notre Dame 58
Appendix D.11 Measles – London School of Hygiene and Tropical Medicine (LSHTM) 59
Appendix D.12 Measles – Pennsylvania State University (PSU) 60
Appendix D.13 MenA - Cambridge 61
Appendix D.14 MenA - KPW 62
Appendix D.15 PCV – London School of Hygiene and Tropical Medicine (LSHTM) and National University of Singapore (NUS) 62
Appendix D.16 Rotavirus – Emory University 63
Appendix D.17 Rubella – University of Georgia 64
Appendix D.18 Rubella – UKHSA 66
Appendix D.19 Typhoid fever - Yale 67
Appendix D.20 Yellow Fever – Imperial College London 69
Appendix D.21 Yellow Fever – University of Notre Dame 69

Appendix E GATHER guidelines 70

Appendix A. Vaccination coverage scenarios

Appendix A.1. Recovery model runs completed in 2022

We describe the details of the vaccination coverage assumptions used for the recovery runs; utilised by a subset of vaccine models to assess the implications of service disruption and routes to recovery. The scenarios for routine immunisation in the presence, or absence, of disruption are visualised in figure A1.

There are three main scenarios: “baseline recovery”, “baseline recovery + catch-up” and “no disruption”. Here baseline recovery (BR) provides the main comparator scenario and relies on available data up to and including 2021, followed by a recovery projection. Baseline recovery + catch-up (BR+C) is identical to BR except it includes catch-up activities, see section Appendix A.1.2 for details. The no disruption (ND) scenario is identical up to 2020 whereupon we project what coverage may have been in 2020 and 2021, followed by our standard forward projections.

Appendix A.1.1. ‘No disruption‘ (ND) scenario

In the ND scenario we project routine immunisation coverage per country/ vaccine for 2020 and 2021 using AutoRegressive Integrated Moving Average modelling (ARIMAs) with logit transform in a manner similar to [1]. For campaigns or SIAs we refer to the WHO campaign tracker and our own full model runs to assess which campaigns were planned but disrupted beyond 2021 due to COVID; we then include these campaigns in the ND scenario but omit them in the BR scenario unless they have been included in the WHO immunisation repository.

Appendix A.1.2. Catch-up activities

We assume catch-up activities take the form of intensified routine immunisation similar to PIRI (periodic intensification of routine immunization). These activities target missed or affected vaccine cohorts where affected cohorts are defined as those individuals who would have received routine immunisation in the ND scenario but did not in the BR scenario ie. the cohorts we projected would have received immunisation if there had been no disruption to coverage. Vaccine cohorts are targeted in the recovery years in stages, see table A1.

Vaccination year	Affected/missed vaccine cohorts targeted
2023	2020 and 2021
2024	2022 and 2023
2025	2024

Table A1: Vaccine year in baseline recovery + catch-up (BR+C) where affected cohorts are targeted with intensified routine catch-up activities.

Whilst the aim of intensified routine is to have fully negative correlation- ie. Only missed or affected cohorts are targeted, to be consistent with other activities described by

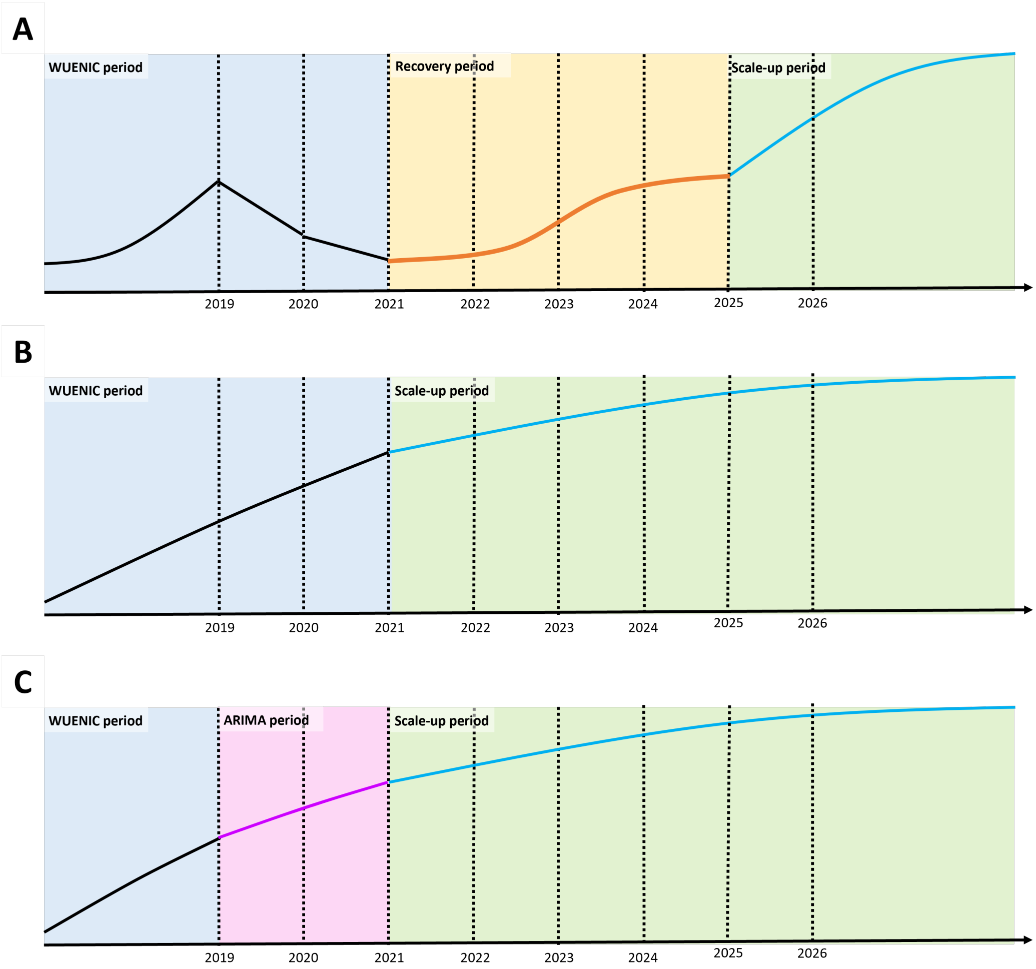

Figure A1: Diagram of projected routine immunisation coverage in the situation where there is disruption in 2020 and 2021 (A), there is not any disruption in 2020 and 2021 (B), and (C) where the no disruption (ND) scenario is projected for 2020 and 2021 using ARIMA. Areas shaded in blue correspond to information provided by WUENIC 2022, areas in yellow highlight the recovery period if there has been disruption, areas in green show the scale-up towards the 2030 endpoints and areas in pink highlight periods projected using ARIMA.

the VIMC, we calculate *necessary coverage* ie. the coverage needed to capture the affected cohorts whilst assuming no correlation. The secretariat will then calculate the dose given under the negative correlation scenario for its impact ratios. An example is given below:

> If ND has achieved coverage of 90% but BR has 80% then 10% of cohorts are affected i.e. 10% of cohorts are missing vaccination due to COVID-related disruption.
>
> If there was perfect negative correlation, the coverage would only need to be the 10% to capture the affected cohort ie. the number of needed to vaccinate if we perfectly targeted the affected cohort is 10% of the total; however, as we assume no correlation, coverage is: 1- (1-cov_{ND})/(1-cov_{BR}). We then account for the overlap in doses to find the estimate of a perfectly targeted activity.

We assume coverage for catch-up activities is 90% of the target population, or 95% for MCV1 or historical highest, whichever is the maximum.
Following the model runs, we calculate the impact ratios for intensified routine activities assuming perfect negative correlation of doses. The issue of dosage correlation is an area of continuing research [2] as the way in which doses are provided given previous vaccination history can affect the estimates of population level immunity and the effectiveness of interventions.

Appendix A.2. Full model runs completed in 2021

We describe the details of the vaccination coverage assumptions used in the full model runs ie. for all vaccines. Figure A2 shows the potential scenarios for routine immunisation coverage. There is only one main scenario, "default".

In figure A2 there are two situations per vaccine-country: one where routine immunisation is disrupted in 2020 and we assume that coverage linearly recovers to 2019 levels by the year 2022 followed by nonlinear scale-up to 2030 endpoints; and one where there is no disruption and scale-up begins in 2021. The non-linear scale-up to 2030 endpoints is described in section Appendix A.3.2.

Campaigns or supplementary immunisation activities (SIAs) are assumed to follow information from the WHO immunisation repository (up to and including 2021) and then are projected given relevant guidance eg. from the EYE strategy for yellow fever, or WHO for measles.

The full model runs were used to estimate impact ratios stratified by country, vaccine and activity type.

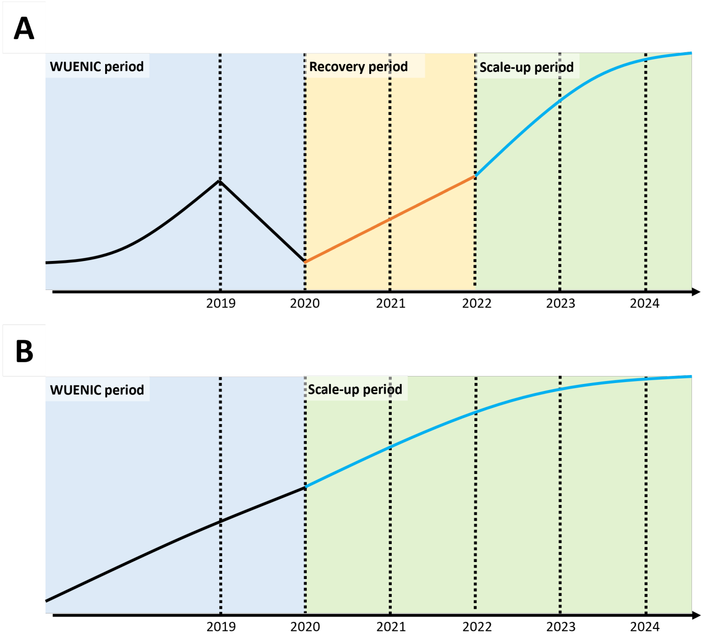

Figure A2: Diagram of projected routine immunisation coverage in the situation where there is disruption in 2020 (A) and there is not any disruption in 2020 (B). Areas shaded in blue correspond to information provided by WUENIC 2021, areas in yellow highlight the recovery period if there has been disruption and areas in green show the scale-up towards the 2030 endpoints.

Appendix A.3. Functional forms for vaccine scale-up or recovery

Appendix A.3.1. Non-linear recovery to pre-pandemic (2019) levels of routine coverage

Many countries included in the 112 analysed here experienced some disruption to health services in 2020 and beyond. We incorporate this into our coverage scenarios through the latest WUENIC, providing information on routine immunisation disruption, and the WHO immunisation repository and campaign tracker, providing information on campaign delivery.

We assume that if there is disruption, ie. 2020 or 2021 levels of coverage are lower than 2019, recovery in routine immunisation coverage achieved will be slow at first then pick up pace, with fastest improvement in 2023 and full recovery (2019 levels) by 2025.

This will take the following, logistic, functional form:

$$y = cov_{21} + \frac{cov_{25} - cov_{21}}{1 + \exp(-k(x-m))}, \tag{A.1}$$

where y is the coverage in year x, cov_{21} is the coverage achieved in 2021, cov_{25} is the coverage aim in 2025, m is the midpoint and k is the rate of coverage increase. In our analysis, $m = 2023$ and $k = 3$ chosen so that the greatest recovery rate is seen in 2023 and the endpoints are within 1% of the actual, or target, coverage.

The functional form of the non-linear recovery is informed by subject area experts on vaccination delivery. The form captures an initial slow start followed by increased effort and thus recovery in alignment with 2023, the year of the 'big catch up'[3]. This recovery relies on the start and end points by country and so will vary on this basis. We did not include a measure of the strength of the health system as this is a nuanced definition by vaccine activity, disease area etc. However, we may expect factors such as healthcare resilience, conflict events or external support to affect recovery times.

For example, if coverage in country 'A' fell from 90% to 80% from 2019 to 2021 we would assume that recovery to pre-pandemic levels (90%) would take until 2025 and take the following values: 2021=80.0%, 2022=80.5%, 2023=85.0%, 2024=89.5% and 2025=90.0%.

Appendix A.3.2. Non-linear scale-up of routine vaccination coverage towards 2030 end-points

For projecting future routine immunisation coverage, we use an adapted form of the function developed for the IA2030 modelling to account for initial timing and coverage [4]. The functional form is as follows:

$$cov_t = 1 - (1 - cov_{t_0}) \exp\left(\log\left(\frac{1 - cov_T}{1 - cov_{t_0}}\right)\frac{t - t_0}{T - t_0}\right), \tag{A.2}$$

where t_0 and T are the baseline and target years respectively; t is the current year and cov is the coverage. This allows us to accommodate rapid scale-up of coverage following

introduction or disruption. The endpoints for the scenarios in 2030 are a conservative down-scaling of the IA2030 targets (see discussion in next section) given expert opinion on the feasibility of reaching those targets. These endpoints directly inform the value of cov_T coverage for routine immunisation in 2030.

For example, if country 'A' was aiming to reach 90% coverage in 2023 and had reached 70% coverage in year $t_0 = 2025$, equation A.2 implies the intermediate years would take the following values: 2025=70.0%, 2026=75.9%, 2027=80.7%, 2028=84.5%, 2029 = 87.5% and 2030=90.0%.

Appendix A.3.3. Defining IA2030 Endpoints

The Immunization Agenda 2030 (IA2030) Framework for Action includes indicators for the monitoring and evaluation (M&E) towards IA2030 goals and strategic priorities. An introduction to the IA2030 goals and subsequent indicators and 2030 targets are included in figure A3[1]. The full breakdown of the M&E framework for the impact goal and strategic priority indicators can be found in IA2030's Annex 1 [5].

[1] i. Vaccine antigens included: HepB, Hib, HPV, JE, measles, MenA, Streptococcus pneumoniae, rotavirus, rubella, yellow fever, diphtheria, tetanus, pertussis, BCG. Measured relative to zero coverage levels (absence of vaccination); target includes deaths averted over the lifetime of the birth cohort by vaccines given during 2021-30. ii. Eradication (polio), elimination of transmission (measles, rubella), elimination as a public health problem (MNT, hepatitis B), control (Japanese encephalitis) iii. Large or disruptive outbreaks of measles, polio, meningococcus, yellow fever, cholera, and Ebola will be defined based on criteria for each disease. iv. Vaccines included: HepB birth dose, Hib, HPV, IPV2, MCV2, PCV, rotavirus, rubella, DTP booster, COVID-19, JE, YF, MenA, multivalent meningitis, typhoid, cholera, dengue, rabies, HepA, influenza, varicella, and mumps. Malaria and other relevant vaccines will potentially be included when recommended. v. COVID-19 vaccination coverage will potentially be included.

Impact Goal		Indicator	2030 Target
**1** Prevent Disease	Save lives	**1.1** Number of future deaths averted through immunization[i]	**50 million future deaths averted globally**
	Control, eliminate & eradicate VPDs	**1.2** Number and % of countries achieving endorsed regional or global VPD control, elimination and eradication targets[ii]	**All countries achieve the endorsed regional or global VPD control, elimination and eradication targets**
	Reduce VPD outbreaks	**1.3** Number of large or disruptive VPD outbreaks[iii]	**All selected VPDs[iii] have a declining trend in the global annual number of large or disruptive outbreaks**
**2** Promote Equity	Leave no one behind	**2.1** Number of zero dose children	**50% reduction in the number of zero dose children at country, regional, and global levels**
	Provide access to all vaccines	**2.2** Introduction of new or under-utilized vaccines[iv] in low and middle income countries	**500 vaccine introductions**
**3** Build strong immunization programmes	Deliver across the life course	**3.1** Vaccination coverage across the life course (DTP3, MCV2, PCV3, HPVc)[v]	**90% global coverage for DTP3, MCV2, PCV3, and HPVc**
	Contribute to PHC/UHC	**3.2** UHC Index of Service Coverage	**Improve UHC Index of Service Coverage at country, regional, and global levels**

Figure A3: IA2030 Impact Goal Indicators and Targets [5].

Appendix B. Additional results

Appendix B.1. List of included countries

In table B1 we list the included low-and-middle-income countries. These were included if they were or are in the Gavi portfolio, or have notable burden, or have notable strategic vaccination activities. These countries represent the majority of global vaccine-preventable disease burden [6].

Appendix B.2. Excess burden due to 2020-2021 coverage disruptions

Table B7: Deaths averted by activities in years of vaccination 2020-2030 by country and scenario for 14 pathogens in 112 countries.

Country	Deaths averted in no-COVID scenario	Deaths averted in baseline recovery scenario	Difference	% Difference
IDN	985,230 (95%[810,954, 1,202,188])	830,923 (95%[687,310, 1,016,793])	154,307 (95%[118,927, 192,128])	19 (95%[15, 22])
PRY	18,296 (95%[16,453, 20,546])	15,549 (95%[13,945, 17,336])	2,747 (95%[2,462, 3,104])	18 (95%[16, 19])
BLZ	921 (95%[732, 1,134])	789 (95%[627, 946])	132 (95%[92, 185])	17 (95%[14, 20])
MMR	285,329 (95%[240,736, 339,196])	245,874 (95%[206,977, 289,785])	39,455 (95%[32,448, 49,436])	16 (95%[14, 18])
ECU	40,535 (95%[35,523, 47,041])	35,165 (95%[30,594, 41,090])	5,370 (95%[4,877, 5,923])	15 (95%[14, 17])
MWI	384,568 (95%[349,363, 418,769])	350,087 (95%[316,885, 382,528])	34,481 (95%[30,241, 38,920])	10 (95%[9, 11])
ZAF	334,843 (95%[298,428, 374,335])	306,371 (95%[271,412, 344,378])	28,471 (95%[25,851, 31,250])	9 (95%[8, 11])
FSM	545 (95%[427, 668])	502 (95%[390, 618])	43 (95%[35, 51])	9 (95%[8, 10])

PHL	503,308 (95%[406,085, 624,674])	461,055 (95%[365,330, 576,904])	42,253 (95%[32,045, 53,112])	9 (95%[7, 11])
BOL	50,514 (95%[42,677, 59,734])	46,783 (95%[39,139, 55,462])	3,731 (95%[3,248, 4,274])	8 (95%[7, 9])
THA	129,430 (95%[110,855, 150,785])	119,582 (95%[100,936, 141,119])	9,849 (95%[9,033, 10,782])	8 (95%[7, 10])
DJI	10,412 (95%[8,816, 11,926])	9,715 (95%[8,237, 11,185])	696 (95%[584, 795])	7 (95%[7, 8])
MDG	347,143 (95%[297,059, 404,511])	324,754 (95%[278,354, 378,327])	22,389 (95%[18,769, 26,184])	7 (95%[6, 8])
PRK	87,303 (95%[71,966, 103,843])	81,803 (95%[67,062, 97,585])	5,500 (95%[4,626, 6,512])	7 (95%[6, 8])
SEN	246,525 (95%[210,525, 288,128])	230,219 (95%[197,814, 271,521])	16,306 (95%[12,059, 23,162])	7 (95%[5, 9])
MKD	2,226 (95%[1,777, 2,632])	2,090 (95%[1,655, 2,493])	137 (95%[118, 156])	7 (95%[5, 8])
VUT	2,477 (95%[1,932, 3,323])	2,323 (95%[1,804, 3,166])	155 (95%[120, 205])	7 (95%[5, 8])
COG	79,743 (95%[62,494, 108,344])	74,685 (95%[59,516, 98,046])	5,058 (95%[1,935, 10,466])	7 (95%[3, 11])
AGO	692,426 (95%[586,463, 792,792])	653,163 (95%[554,058, 747,358])	39,264 (95%[32,727, 45,434])	6 (95%[6, 6])
HND	29,404 (95%[25,483, 33,789])	27,622 (95%[24,182, 31,429])	1,783 (95%[1,293, 2,529])	6 (95%[5, 9])
PNG	71,287 (95%[47,026, 111,024])	67,125 (95%[43,938, 104,856])	4,162 (95%[2,661, 6,959])	6 (95%[5, 9])
JAM	3,640 (95%[3,127, 4,090])	3,444 (95%[2,941, 3,894])	196 (95%[173, 220])	6 (95%[5, 7])

ZMB	436,537 (95%[397,146, 483,761])	411,676 (95%[373,444, 457,214])	24,861 (95%[22,085, 27,784])	6 (95%[5, 7])
PER	106,014 (95%[95,173, 120,195])	100,467 (95%[89,811, 114,727])	5,548 (95%[5,101, 6,039])	6 (95%[5, 6])
JOR	24,272 (95%[20,959, 27,225])	23,108 (95%[19,902, 25,969])	1,164 (95%[1,002, 1,314])	5 (95%[5, 6])
COL	82,206 (95%[69,217, 98,188])	78,560 (95%[65,456, 94,636])	3,646 (95%[3,346, 3,942])	5 (95%[4, 6])
RWA	167,466 (95%[149,948, 191,027])	160,443 (95%[143,664, 182,954])	7,023 (95%[6,179, 8,166])	4 (95%[4, 5])
SYR	57,253 (95%[46,725, 71,149])	54,810 (95%[44,876, 67,983])	2,443 (95%[1,868, 3,244])	4 (95%[4, 5])
UGA	978,117 (95%[849,195, 1,081,488])	938,345 (95%[817,515, 1,040,665])	39,772 (95%[30,917, 48,377])	4 (95%[3, 5])
GNB	40,549 (95%[32,664, 50,291])	39,183 (95%[31,527, 48,958])	1,366 (95%[1,134, 1,640])	4 (95%[3, 4])
LBR	104,411 (95%[88,348, 123,573])	100,874 (95%[85,381, 119,582])	3,537 (95%[2,864, 4,476])	4 (95%[3, 4])
CIV	533,163 (95%[441,179, 629,807])	516,765 (95%[429,027, 610,132])	16,398 (95%[12,339, 21,410])	3 (95%[3, 4])
ETH	2,341,610 (95%[2,146,179, 2,594,484])	2,267,655 (95%[2,077,494, 2,507,250])	73,955 (95%[65,798, 84,072])	3 (95%[3, 4])
IND	4,675,244 (95%[3,881,450, 5,689,617])	4,534,526 (95%[3,779,912, 5,507,836])	140,717 (95%[103,850, 180,657])	3 (95%[3, 4])
MRT	75,936 (95%[67,307, 85,466])	73,430 (95%[64,905, 82,659])	2,506 (95%[2,114, 3,018])	3 (95%[3, 4])

UKR	32,952 (95%[28,579, 38,357])	31,975 (95%[27,883, 37,068])	978 (95%[749, 1,296])	3 (95%[3, 4])
VNM	270,176 (95%[225,772, 319,172])	261,175 (95%[218,129, 308,046])	9,000 (95%[7,113, 10,973])	3 (95%[3, 4])
TZA	1,092,465 (95%[990,229, 1,185,181])	1,060,316 (95%[961,581, 1,150,428])	32,149 (95%[28,061, 36,048])	3 (95%[3, 3])
UZB	122,372 (95%[98,282, 150,601])	118,605 (95%[95,385, 146,695])	3,767 (95%[2,742, 5,784])	3 (95%[2, 5])
AFG	344,932 (95%[293,900, 406,596])	335,542 (95%[285,313, 395,325])	9,390 (95%[7,437, 11,751])	3 (95%[2, 3])
LKA	51,709 (95%[44,498, 60,250])	50,297 (95%[43,159, 58,694])	1,412 (95%[1,281, 1,547])	3 (95%[2, 3])
MDA	7,162 (95%[5,838, 8,768])	6,986 (95%[5,687, 8,552])	176 (95%[142, 215])	3 (95%[2, 3])
MOZ	622,218 (95%[556,884, 676,662])	606,684 (95%[543,487, 660,763])	15,534 (95%[12,581, 18,150])	3 (95%[2, 3])
GMB	52,838 (95%[45,847, 61,233])	51,605 (95%[44,876, 59,882])	1,234 (95%[1,006, 1,510])	2 (95%[2, 3])
GTM	60,674 (95%[54,882, 67,439])	59,333 (95%[53,691, 65,973])	1,341 (95%[1,108, 1,553])	2 (95%[2, 3])
NIC	25,557 (95%[22,945, 27,752])	24,953 (95%[22,405, 27,099])	604 (95%[538, 665])	2 (95%[2, 3])
SDN	497,890 (95%[439,550, 563,529])	488,090 (95%[430,049, 552,137])	9,799 (95%[7,569, 12,620])	2 (95%[2, 3])
SRB	7,608 (95%[6,680, 8,914])	7,431 (95%[6,505, 8,723])	177 (95%[160, 198])	2 (95%[2, 3])

SWZ	16,094 (95%[14,423, 17,617])	15,726 (95%[14,098, 17,215])	368 (95%[310, 416])	2 (95%[2, 3])
TGO	145,683 (95%[127,775, 166,266])	142,147 (95%[124,451, 162,107])	3,536 (95%[2,879, 4,140])	2 (95%[2, 3])
XK	2,821 (95%[2,386, 3,227])	2,759 (95%[2,330, 3,161])	62 (95%[52, 70])	2 (95%[2, 3])
AZE	21,991 (95%[17,618, 26,164])	21,612 (95%[17,298, 25,758])	378 (95%[312, 440])	2 (95%[2, 2])
KIR	1,395 (95%[1,165, 1,719])	1,368 (95%[1,142, 1,689])	27 (95%[23, 31])	2 (95%[2, 2])
SLV	10,666 (95%[9,485, 11,743])	10,442 (95%[9,292, 11,503])	224 (95%[183, 267])	2 (95%[2, 2])
TJK	43,735 (95%[34,772, 53,385])	42,874 (95%[33,599, 52,829])	861 (95%[278, 2,054])	2 (95%[1, 5])
KGZ	31,892 (95%[27,952, 36,600])	31,293 (95%[27,471, 35,942])	599 (95%[439, 794])	2 (95%[1, 3])
STP	5,741 (95%[2,059, 26,781])	5,660 (95%[1,997, 26,610])	81 (95%[57, 171])	2 (95%[1, 3])
BIH	2,863 (95%[2,508, 3,242])	2,819 (95%[2,468, 3,192])	43 (95%[33, 54])	2 (95%[1, 2])
CAF	110,699 (95%[93,307, 129,709])	108,823 (95%[91,763, 127,509])	1,876 (95%[1,267, 2,736])	2 (95%[1, 2])
CMR	553,310 (95%[469,675, 647,992])	543,644 (95%[462,294, 632,356])	9,666 (95%[5,209, 15,085])	2 (95%[1, 2])
GEO	7,004 (95%[5,872, 8,289])	6,892 (95%[5,774, 8,159])	113 (95%[94, 135])	2 (95%[1, 2])
PAK	1,749,777 (95%[1,520,064, 1,982,199])	1,721,591 (95%[1,496,398, 1,953,433])	28,186 (95%[23,815, 34,909])	2 (95%[1, 2])
VEN	61,055 (95%[53,651, 71,265])	60,127 (95%[52,347, 70,498])	927 (95%[-204, 1,946])	2 (95%[0, 3])

SLB	7,664 (95%[6,017, 10,210])	7,562 (95%[5,922, 10,068])	102 (95%[67, 236])	1 (95%[1, 3])
ARM	4,322 (95%[3,662, 5,047])	4,268 (95%[3,599, 4,988])	54 (95%[39, 70])	1 (95%[1, 2])
HTI	84,158 (95%[67,099, 99,955])	82,908 (95%[66,260, 98,423])	1,250 (95%[601, 1,916])	1 (95%[1, 2])
KHM	85,453 (95%[70,604, 104,115])	84,307 (95%[69,701, 102,112])	1,146 (95%[855, 1,554])	1 (95%[1, 2])
LAO	44,988 (95%[37,957, 52,522])	44,393 (95%[37,438, 51,842])	595 (95%[429, 969])	1 (95%[1, 2])
SLE	168,393 (95%[141,766, 202,295])	166,571 (95%[140,206, 199,873])	1,822 (95%[1,349, 2,762])	1 (95%[1, 2])
ZWE	267,874 (95%[244,278, 293,411])	264,186 (95%[241,050, 289,609])	3,688 (95%[3,197, 4,222])	1 (95%[1, 2])
TKM	40,955 (95%[35,976, 48,126])	41,235 (95%[36,248, 48,443])	-280 (95%[-317, -246])	-1 (95%[-1, -1])
ALB	4,008 (95%[3,471, 4,757])	3,965 (95%[3,429, 4,706])	43 (95%[32, 51])	1 (95%[1, 1])
BDI	290,919 (95%[252,776, 335,393])	288,632 (95%[250,886, 333,020])	2,287 (95%[1,776, 3,554])	1 (95%[1, 1])
BTN	2,217 (95%[1,705, 2,714])	2,200 (95%[1,690, 2,693])	17 (95%[15, 21])	1 (95%[1, 1])
CPV	3,809 (95%[3,275, 4,472])	3,778 (95%[3,249, 4,437])	31 (95%[23, 36])	1 (95%[1, 1])
MLI	576,331 (95%[502,281, 649,999])	572,866 (95%[499,183, 646,306])	3,465 (95%[2,654, 4,508])	1 (95%[1, 1])
NER	736,499 (95%[663,956, 812,887])	731,747 (95%[659,948, 807,922])	4,751 (95%[3,789, 6,620])	1 (95%[1, 1])

NPL	127,046 (95%[110,057, 144,409])	125,822 (95%[108,983, 143,130])	1,224 (95%[952, 1,447])	1 (95%[1, 1])
SSD	220,866 (95%[181,646, 263,033])	219,556 (95%[180,422, 261,674])	1,310 (95%[1,155, 1,476])	1 (95%[1, 1])
TLS	8,426 (95%[6,838, 10,031])	8,351 (95%[6,760, 9,954])	76 (95%[66, 87])	1 (95%[1, 1])
PSE	21,593 (95%[17,147, 28,426])	21,711 (95%[17,263, 28,543])	-118 (95%[-136, -101])	-1 (95%[-1, 0])
IRQ	141,854 (95%[125,127, 163,606])	140,132 (95%[124,709, 159,149])	1,722 (95%[-637, 4,403])	1 (95%[0, 3])
YEM	175,142 (95%[136,530, 264,518])	173,737 (95%[136,100, 258,086])	1,405 (95%[-485, 5,887])	1 (95%[0, 3])
BFA	446,174 (95%[393,454, 492,504])	443,817 (95%[391,537, 489,367])	2,357 (95%[937, 4,491])	1 (95%[0, 1])
COD	3,315,344 (95%[2,496,865, 4,253,500])	3,293,803 (95%[2,476,901, 4,228,602])	21,541 (95%[12,680, 31,334])	1 (95%[0, 1])
GUY	2,733 (95%[2,301, 3,148])	2,709 (95%[2,287, 3,121])	24 (95%[12, 35])	1 (95%[0, 1])
MHL	160 (95%[108, 223])	159 (95%[107, 222])	1 (95%[1, 1])	1 (95%[0, 1])
MNG	24,677 (95%[20,611, 29,416])	24,549 (95%[20,501, 29,274])	128 (95%[110, 148])	1 (95%[0, 1])
NGA	4,385,556 (95%[3,741,371, 5,044,918])	4,351,778 (95%[3,717,600, 4,997,317])	33,779 (95%[19,654, 52,202])	1 (95%[0, 1])
BGD	556,559 (95%[461,320, 667,645])	556,039 (95%[460,362, 669,122])	520 (95%[-3,230, 1,474])	0 (95%[-1, 0])
BEN	274,061 (95%[235,879, 325,455])	273,240 (95%[235,344, 324,553])	821 (95%[460, 1,652])	0 (95%[0, 1])

DZA	192,172 (95%[168,400, 215,970])	191,228 (95%[167,921, 214,617])	944 (95%[433, 1,352])	0 (95%[0, 1])
GIN	230,579 (95%[189,345, 273,163])	229,644 (95%[188,446, 272,037])	935 (95%[611, 1,358])	0 (95%[0, 1])
TUN	22,026 (95%[19,337, 24,549])	21,922 (95%[19,242, 24,407])	104 (95%[85, 131])	0 (95%[0, 1])
WSM	1,230 (95%[929, 1,776])	1,225 (95%[924, 1,772])	5 (95%[4, 6])	0 (95%[0, 1])
BLR	10,492 (95%[9,061, 11,869])	10,472 (95%[9,042, 11,849])	20 (95%[18, 22])	0 (95%[0, 0])
CHN	2,659,118 (95%[2,241,551, 3,126,575])	2,648,452 (95%[2,232,825, 3,113,948])	10,666 (95%[8,554, 12,903])	0 (95%[0, 0])
COM	13,146 (95%[11,131, 15,263])	13,112 (95%[11,101, 15,228])	34 (95%[29, 39])	0 (95%[0, 0])
CUB	11,424 (95%[9,855, 12,869])	11,392 (95%[9,825, 12,833])	32 (95%[28, 36])	0 (95%[0, 0])
EGY	394,483 (95%[338,366, 463,320])	394,141 (95%[338,036, 462,927])	342 (95%[304, 388])	0 (95%[0, 0])
ERI	36,485 (95%[30,650, 41,749])	36,452 (95%[30,622, 41,714])	33 (95%[23, 43])	0 (95%[0, 0])
FJI	5,025 (95%[4,081, 6,399])	5,025 (95%[4,081, 6,399])	0 (95%[-1, 0])	0 (95%[0, 0])
GHA	539,675 (95%[462,462, 618,954])	537,675 (95%[460,690, 616,844])	2,000 (95%[1,592, 2,314])	0 (95%[0, 0])
IRN	150,223 (95%[128,536, 179,212])	149,936 (95%[128,287, 178,872])	286 (95%[256, 326])	0 (95%[0, 0])
KEN	485,987 (95%[437,157, 536,415])	485,701 (95%[436,883, 536,199])	286 (95%[-2, 436])	0 (95%[0, 0])

LSO	27,324 (95%[23,905, 30,761])	27,275 (95%[23,860, 30,715])	48 (95%[37, 63])	0 (95%[0, 0])
MAR	127,781 (95%[117,144, 139,247])	127,579 (95%[116,954, 139,055])	202 (95%[179, 230])	0 (95%[0, 0])
NAM	19,216 (95%[17,307, 21,405])	19,211 (95%[17,302, 21,399])	6 (95%[5, 10])	0 (95%[0, 0])
SOM	305,466 (95%[244,481, 361,164])	304,334 (95%[243,521, 359,968])	1,132 (95%[890, 1,388])	0 (95%[0, 0])
TCD	429,378 (95%[353,979, 509,950])	429,843 (95%[354,372, 510,769])	-465 (95%[-818, -290])	0 (95%[0, 0])
TON	728 (95%[582, 870])	727 (95%[581, 869])	1 (95%[1, 1])	0 (95%[0, 0])
TUV	56 (95%[47, 65])	56 (95%[47, 65])	0 (95%[0, 0])	0 (95%[0, 0])

Table B1: List of included countries for VIMC.

Country	Country name
AFG	Afghanistan
AGO	Angola
ALB	Albania
ARM	Armenia
AZE	Azerbaijan
BDI	Burundi
BEN	Benin
BFA	Burkina Faso
BGD	Bangladesh
BIH	Bosnia and Herzegovina
BLR	Belarus
BLZ	Belize
BOL	Bolivia, Plurinational State of
BTN	Bhutan
CAF	Central African Republic
CHN	China
CIV	Cote d'Ivoire
CMR	Cameroon
COD	Congo, the Democratic Republic of the
COG	Congo
COL	Colombia
COM	Comoros
CPV	Cabo Verde
CUB	Cuba
DJI	Djibouti
DZA	Algeria
ECU	Ecuador
EGY	Egypt
ERI	Eritrea
ETH	Ethiopia
FJI	Fiji
FSM	Micronesia, Federated States of
GEO	Georgia
GHA	Ghana
GIN	Guinea
GMB	Gambia
GNB	Guinea-Bissau
GTM	Guatemala
GUY	Guyana
HND	Honduras
HTI	Haiti
IDN	Indonesia
IND	India
IRN	Iran, Islamic Republic of
IRQ	Iraq
JAM	Jamaica
JOR	Jordan
KEN	Kenya
KGZ	Kyrgyzstan
KHM	Cambodia
KIR	Kiribati
LAO	Lao People's Democratic Republic
LBR	Liberia
LKA	Sri Lanka
LSO	Lesotho
MAR	Morocco
MDA	Moldova, Republic of
MDG	Madagascar
MHL	Marshall Islands
MKD	Macedonia, the former Yugoslav Republic of
MLI	Mali
MMR	Myanmar
MNG	Mongolia
MOZ	Mozambique
MRT	Mauritania
MWI	Malawi
NAM	Namibia
NER	Niger
NGA	Nigeria
NIC	Nicaragua
NPL	Nepal
PAK	Pakistan
PER	Peru
PHL	Philippines
PNG	Papua New Guinea
PRK	Korea, Democratic People's Republic of
PRY	Paraguay
PSE	Palestine, State of
RWA	Rwanda
SDN	Sudan
SEN	Senegal
SLB	Solomon Islands
SLE	Sierra Leone
SLV	El Salvador
SOM	Somalia
SRB	Serbia
SSD	South Sudan
STP	Sao Tome and Principe
SWZ	Swaziland
SYR	Syrian Arab Republic
TCD	Chad
TGO	Togo
THA	Thailand
TJK	Tajikistan
TKM	Turkmenistan
TLS	Timor-Leste
TON	Tonga
TUN	Tunisia
TUV	Tuvalu
TZA	Tanzania, United Republic of
UGA	Uganda
UKR	Ukraine
UZB	Uzbekistan
VEN	Venezuela, Bolivarian Republic of
VNM	Viet Nam
VUT	Vanuatu
WSM	Samoa
XK	Kosovo
YEM	Yemen
ZAF	South Africa
ZMB	Zambia
ZWE	Zimbabwe

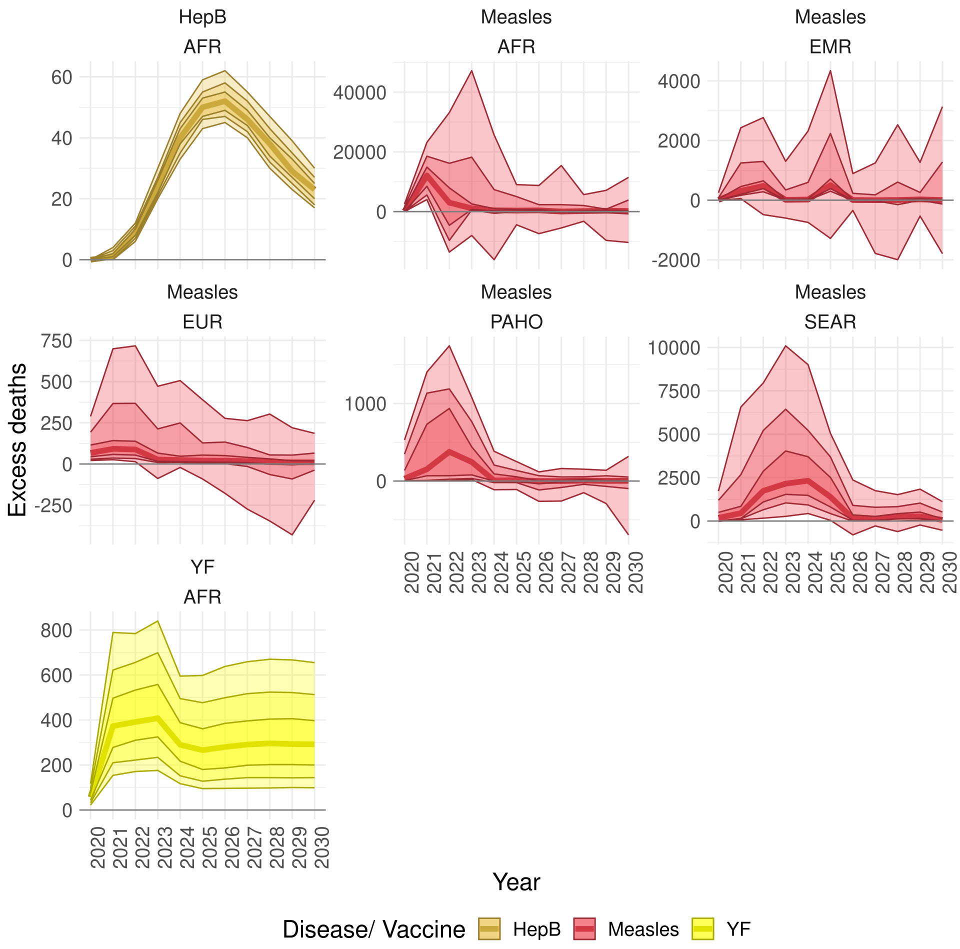

Figure B1: Excess deaths by calendar year due to coverage disruptions for each disease/WHO region. Time series for disease/vaccine-WHO region combinations where the median additional deaths do not exceed 50 in at least one year are not shown. The ribbons show 95%CrI in the estimates. The median estimate is shown with a solid line. Similar figures for cases and DALYs are provided in the appendix. Note y-axis scales vary substantially by panel. There are 42 VIMC countries in AFR, 14 VIMC countries in EMR, 15 VIMC countries in EUR, 15 VIMC countries in PAHO, 10 VIMC countries in SEAR and 16 VIMC countries in WPR Western Pacific Region.

Table B2: Proportion of excess deaths averted for years of vaccination 2020-2030 that are due to disruption in each activity type. Campaign here also applies to MAC and SIAs.

Disease	Activity type	Proportion
Diphtheria	routine	1 [1, 1]
HepB	routine	1 [1, 1]
Hib	routine	1 [1, 1]
HPV	campaign	0 [0, 0]
HPV	routine	1 [1, 1]
JE	campaign	0 [0, 0]
JE	routine	1 [1, 1]
Measles	campaign	0.086 [0.0602, 0.1172]
Measles	routine	0.914 [0.8828, 0.9398]
MenA	campaign	0.0596 [0.0247, 0.0973]
MenA	routine	0.9404 [0.9027, 0.9753]
PCV	routine	1 [1, 1]
Pertussis	routine	1 [1, 1]
Rota	routine	1 [1, 1]
Rubella	campaign	0.9382 [0.3448, 2.9912]
Rubella	routine	0.0618 [-1.9912, 0.6552]
Tetanus	routine	1 [1, 1]
Typhoid	campaign	0.1532 [0.0318, 0.315]
Typhoid	routine	0.8468 [0.685, 0.9682]
YF	campaign	0.2314 [0.161, 0.3141]
YF	routine	0.7686 [0.6859, 0.839]

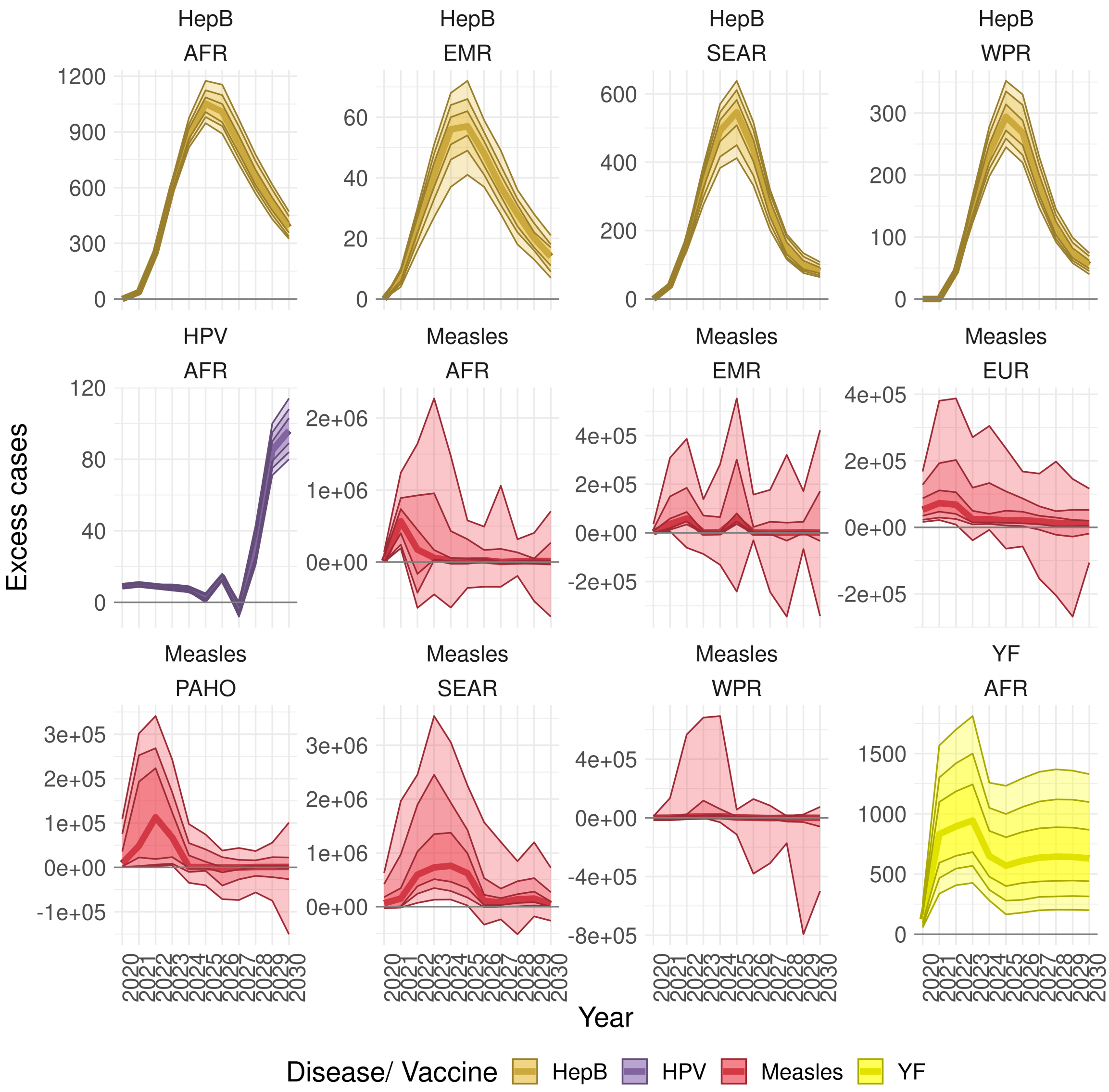

Figure B2: Excess cases by calendar year due to coverage disruptions for each disease/ WHO region. Time series were the median additional cases do not exceed 50 in any one year are not shown. The ribbons show 95%CrI, 80%CrI and 50%CrI in the estimates. The median estimate is shown with a solid line.

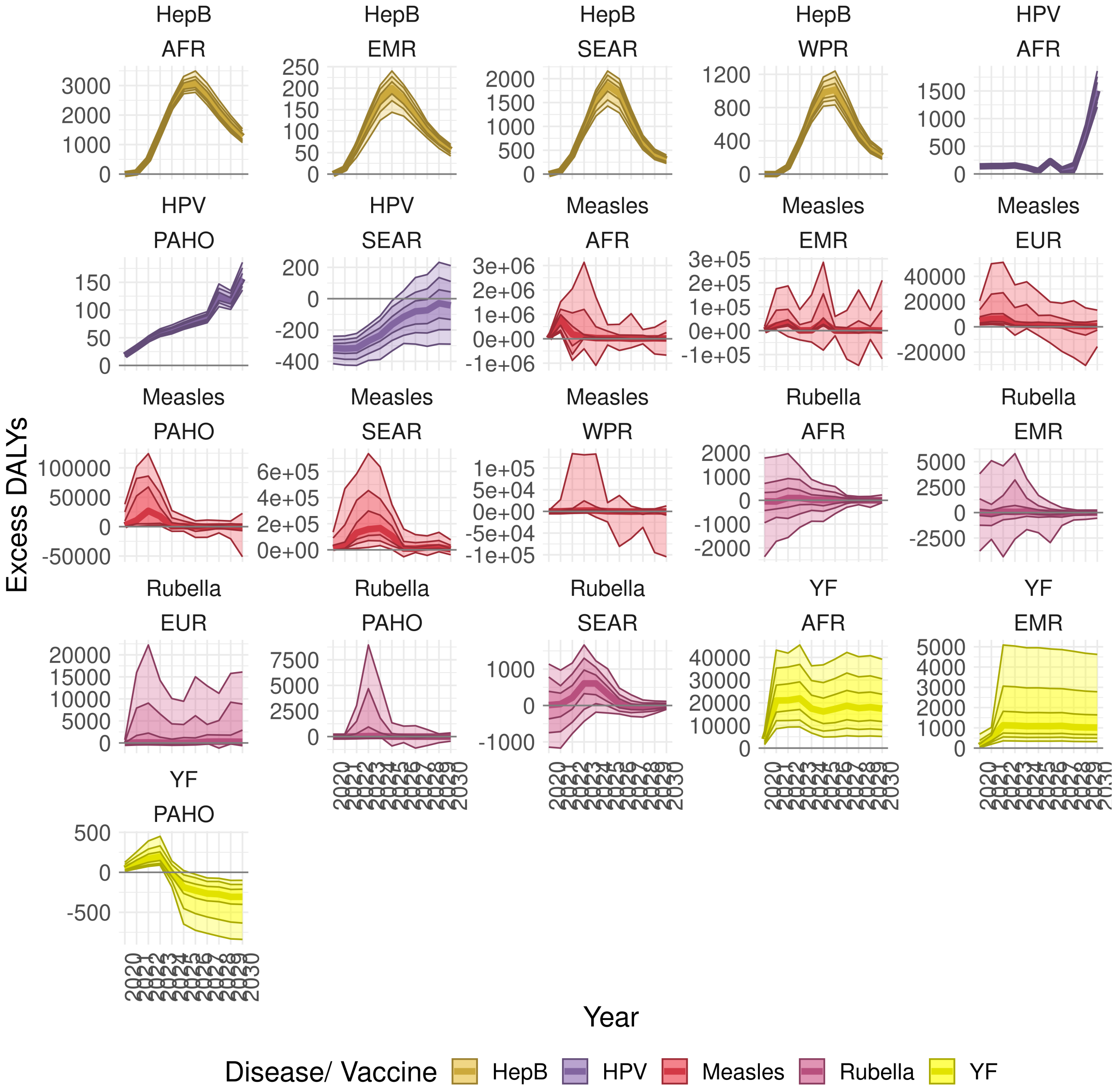

Figure B3: Excess DALYs by calendar year due to coverage disruptions for each disease/ WHO region. Time series were the median additional DALYs do not exceed 50 in any one year are not shown. The ribbons show 95%CrI, 80%CrI and 50%CrI in the estimates. The median estimate is shown with a solid line.

Table B3: Excess deaths due to coverage disruption for calendar years 2020-2030 calculated by comparing the baseline-recovery and no-disruption scenarios. Mean estimates are shown with 95%CrI shown in brackets, median estimates are shown after 'med='. *MenA was run for five countries only. There are 42 VIMC countries in AFR, 14 VIMC countries in EMR, 15 VIMC countries in EUR, 15 VIMC countries in PAHO, 10 VIMC countries in SEAR and 16 VIMC countries in WPR.

Disease	AFR	EMR	EUR	PAHO	SEAR	WPR	Total
HPV	96 (95%[86, 106]) med=96	0 (95%[0, 0]) med=0	1 (95%[1, 1]) med=1	13 (95%[12, 15]) med=13	-86 (95%[-123, -53]) med=-85	3 (95%[2, 4]) med=3	26 (95%[-12, 61]) med=29
HepB	315 (95%[279, 354]) med=312	15 (95%[9, 22]) med=15	0 (95%[0, 0]) med=0	0 (95%[0, 0]) med=0	122 (95%[95, 143]) med=122	67 (95%[48, 82]) med=67	518 (95%[456, 572]) med=520
Measles	26,498 (95%[4,884, 79,034]) med=17,637	2,918 (95%[927, 13,999]) med=1,444	642 (95%[283, 1,360]) med=456	1,389 (95%[74, 3,849]) med=1,027	12,899 (95%[5,529, 35,276]) med=9,727	199 (95%[-221, 1,565]) med=77	44,544 (95%[13,794, 130,657]) med=30,435
MenA	2 (95%[0, 11]) med=0	NA	NA	NA	NA	NA	2 (95%[0, 11]) med=0
Rubella	2 (95%[-70, 47]) med=4	14 (95%[-265, 287]) med=5	264 (95%[-1, 946]) med=211	30 (95%[-2, 160]) med=6	19 (95%[-5, 75]) med=29	0 (95%[-15, 13]) med=0	329 (95%[-107, 1,148]) med=303
YF	3,499 (95%[1,247, 6,817]) med=3,200	217 (95%[50, 701]) med=162	NA	-15 (95%[-58, 18]) med=-12	NA	NA	3,701 (95%[1,455, 7,003]) med=3,463
Total	30,410 (95%[9,033, 83,115]) med=21,351	3,163 (95%[973, 14,568]) med=1,693	906 (95%[318, 1,794]) med=847	1,417 (95%[77, 3,907]) med=1,090	12,954 (95%[5,551, 35,321]) med=9,819	268 (95%[-162, 1,629]) med=150	49,119 (95%[17,248, 134,941]) med=34,713

Table B4: Excess cases due to coverage disruption for calendar years 2020-2030 calculated by comparing the baseline-recovery and no-disruption scenarios. Mean estimates are shown with 95%CrI shown in brackets, median estimates are shown after 'med='. *MenA was run for five countries only. There are 42 VIMC countries in AFR, 14 VIMC countries in EMR, 15 VIMC countries in EUR, 15 VIMC countries in PAHO, 10 VIMC countries in SEAR and 16 VIMC countries in WPR. Totals are not included across diseases as cases do not have a common definition.

Disease	AFR	EMR	EUR	PAHO	SEAR	WPR	Total
HPV	269 (95%[231, 315]) med=267	0 (95%[0, 0]) med=0	1 (95%[1, 1]) med=1	21 (95%[17, 24]) med=20	-143 (95%[-218, -78]) med=-141	6 (95%[4, 7]) med=6	153 (95%[73, 227]) med=152
HepB	6,235 (95%[5,524, 7,103]) med=6,216	332 (95%[244, 404]) med=333	21 (95%[9, 35]) med=21	41 (95%[14, 67]) med=41	2,598 (95%[2,060, 3,065]) med=2,613	1,421 (95%[1,164, 1,727]) med=1,409	10,648 (95%[9,466, 11,845]) med=10,670
Measles	1,443,000 (95%[252,896, 4,294,211]) med=1,017,918	343,944 (95%[105,393, 1,369,510]) med=181,409	490,537 (95%[252,062, 961,669]) med=392,895	344,862 (95%[21,294, 765,795]) med=278,117	4,911,221 (95%[2,103,449, 11,422,589]) med=4,093,641	54,790 (95%[-175,332, 400,862]) med=32,599	7,588,354 (95%[3,020,945, 17,701,459]) med=6,167,862
MenA	17 (95%[0, 135]) med=0	NA	NA	NA	NA	NA	17 (95%[0, 135]) med=0
Rubella	5 (95%[-153, 104]) med=9	42 (95%[-644, 786]) med=13	795 (95%[-3, 2,625]) med=658	100 (95%[-5, 507]) med=15	16 (95%[-8, 155]) med=64	0 (95%[-29, 30]) med=0	958 (95%[-248, 2,801]) med=865
YF	7,618 (95%[2,818, 14,587]) med=7,105	544 (95%[145, 1,809]) med=403	NA	-41 (95%[-123, 11]) med=-35	NA	NA	8,121 (95%[3,016, 15,177]) med=7,628

Table B5: Excess DALYs due to coverage disruption for calendar years 2020-2030 calculated by comparing the baseline-recovery and no-disruption scenarios. Mean estimates are shown with 95%CrI shown in brackets, median estimates are shown after 'med='. *MenA was run for five countries only. There are 42 VIMC countries in AFR, 14 VIMC countries in EMR, 15 VIMC countries in EUR, 15 VIMC countries in PAHO, 10 VIMC countries in SEAR and 16 VIMC countries in WPR.

Disease	AFR	EMR	EUR	PAHO	SEAR	WPR	Total
HPV	3,387 (95%[2,994, 3,894]) med=3,377	-1 (95%[-1, 0]) med=0	48 (95%[41, 54]) med=47	859 (95%[775, 971]) med=854	-1,980 (95%[-3,648, -336]) med=-1,894	122 (95%[98, 149]) med=122	2,434 (95%[745, 4,103]) med=2,472
HepB	18,111 (95%[16,190, 20,524]) med=18,092	1,113 (95%[853, 1,324]) med=1,115	56 (95%[30, 79]) med=57	164 (95%[136, 201]) med=164	8,956 (95%[7,116, 10,570]) med=9,010	5,028 (95%[4,142, 6,080]) med=4,994	33,429 (95%[29,906, 37,087]) med=33,475
Measles	1,727,977 (95%[297,966, 5,142,464]) med=1,149,607	200,097 (95%[63,454, 955,025]) med=101,553	44,958 (95%[19,228, 97,257]) med=31,351	100,343 (95%[5,267, 280,684]) med=72,057	938,553 (95%[409,327, 2,516,153]) med=714,294	13,687 (95%[-15,376, 111,063]) med=5,304	3,025,616 (95%[956,260, 8,799,095]) med=2,070,177
MenA	111 (95%[0, 828]) med=0	NA	NA	NA	NA	NA	111 (95%[0, 828]) med=0
Rubella	125 (95%[-4,018, 2,960]) med=208	1,003 (95%[-15,869, 19,625]) med=337	21,316 (95%[-43, 72,848]) med=16,801	2,300 (95%[-81, 11,605]) med=358	318 (95%[-230, 3,821]) med=1,616	4 (95%[-577, 618]) med=0	25,066 (95%[-6,230, 79,145]) med=22,616
YF	203,849 (95%[70,427, 407,115]) med=188,545	13,964 (95%[3,306, 45,556]) med=10,366	NA	-1,258 (95%[-3,983, 500]) med=-1,040	NA	NA	216,555 (95%[81,738, 415,720]) med=202,571
Total	1,953,559 (95%[559,623, 5,309,858]) med=1,340,218	216,177 (95%[72,045, 991,795]) med=117,034	66,378 (95%[21,496, 128,028]) med=63,492	102,409 (95%[5,859, 288,488]) med=75,846	945,848 (95%[418,984, 2,523,860]) med=721,485	18,841 (95%[-10,745, 115,866]) med=10,477	3,303,211 (95%[1,157,510, 8,986,297]) med=2,376,996

Table B6: Projected numbers of excess deaths due to coverage disruption between years of vaccination 2020 and 2030 calculated by comparing baseline-recovery and no-disruption scenarios. This approach extrapolates impact using impact ratios and performs best for static models. 95%CrI are given in square brackets.

**Disease**	**AFR**	**EMR**	**PAHO**	**SEAR**	**WPR**	**EUR**	**Total**
**Diphtheria**	81 [-1, 273]	39 [-2, 140]	6 [0, 18]	240 [-46, 886]	24 [-5, 113]	1 [0, 3]	**4,298 [4,298, 4,298]**
**HPV**	11,289 [-3,336, 28,794]	3 [-165, 118]	1,449 [0, 4,472]	11,152 [0, 25,205]	1,644 [0, 3,786]	239 [0, 349]	**282,177 [272,850, 298,738]**
**HepB**	6,028 [0, 18,930]	1,163 [-144, 4,189]	216 [0, 587]	14,028 [0, 37,479]	3,843 [0, 12,722]	137 [0, 392]	**279,566 [245,199, 320,894]**
**Hib**	1,626 [-927, 7,368]	403 [-365, 1,998]	160 [0, 456]	833 [-1,259, 5,318]	140 [-37, 765]	8 [-10, 77]	**34,873 [27,443, 42,301]**
**JE**	~	0 [0, 0]	~	245 [0, 662]	47 [0, 122]	~	**3,205 [1,723, 5,028]**
**Measles**	16,117 [0, 61,764]	2,783 [0, 7,028]	534 [-189, 2,161]	4,377 [-543, 16,501]	326 [-580, 2,540]	94 [-74, 1,069]	**265,221 [229,551, 288,047]**
**MenA**	38 [0, 196]	78 [-20, 442]	~	~	~	~	**1,280 [835, 1,734]**
**PCV**	2,198 [0, 7,786]	145 [-177, 814]	77 [-235, 334]	1,359 [-2,245, 7,450]	182 [0, 605]	0 [-27, 81]	**43,574 [7,613, 78,903]**
**Pertussis**	3,576 [-79, 12,177]	1,208 [-94, 4,205]	205 [-1, 661]	5,799 [-1,141, 21,539]	650 [-86, 2,929]	29 [-7, 116]	**126,138 [126,138, 126,138]**
**Rota**	737 [0, 2,485]	65 [-30, 315]	27 [-75, 133]	37 [-43, 204]	-76 [-1,690, 1,045]	11 [0, 40]	**8,804 [2,155, 14,399]**
**Rubella**	274 [0, 2,001]	14 [0, 67]	42 [-18, 370]	-176 [-957, 661]	16 [-25, 121]	162 [-5, 2,220]	**3,640 [93, 10,733]**
**Tetanus**	286 [-3, 976]	130 [-5, 452]	23 [0, 73]	541 [-109, 2,008]	67 [-4, 294]	3 [-1, 11]	**11,560 [11,560, 11,560]**
**Typhoid**	847 [-782, 5,149]	310 [-166, 3,041]	-15 [-122, 0]	71 [-2,237, 3,142]	126 [-189, 927]	-7 [-58, 0]	**14,987 [2,238, 50,370]**
**YF**	2,684 [0, 13,696]	0 [0, 0]	5 [-66, 119]	~	~	~	**31,501 [12,612, 56,362]**
**Total**	**457,371 [423,382, 500,319]**	**56,753 [49,428, 71,151]**	**27,425 [25,510, 29,864]**	**353,077 [296,821, 413,783]**	**67,947 [57,444, 79,643]**	**7,128 [5,396, 10,388]**	**967,635 [896,596, 1,049,981]**

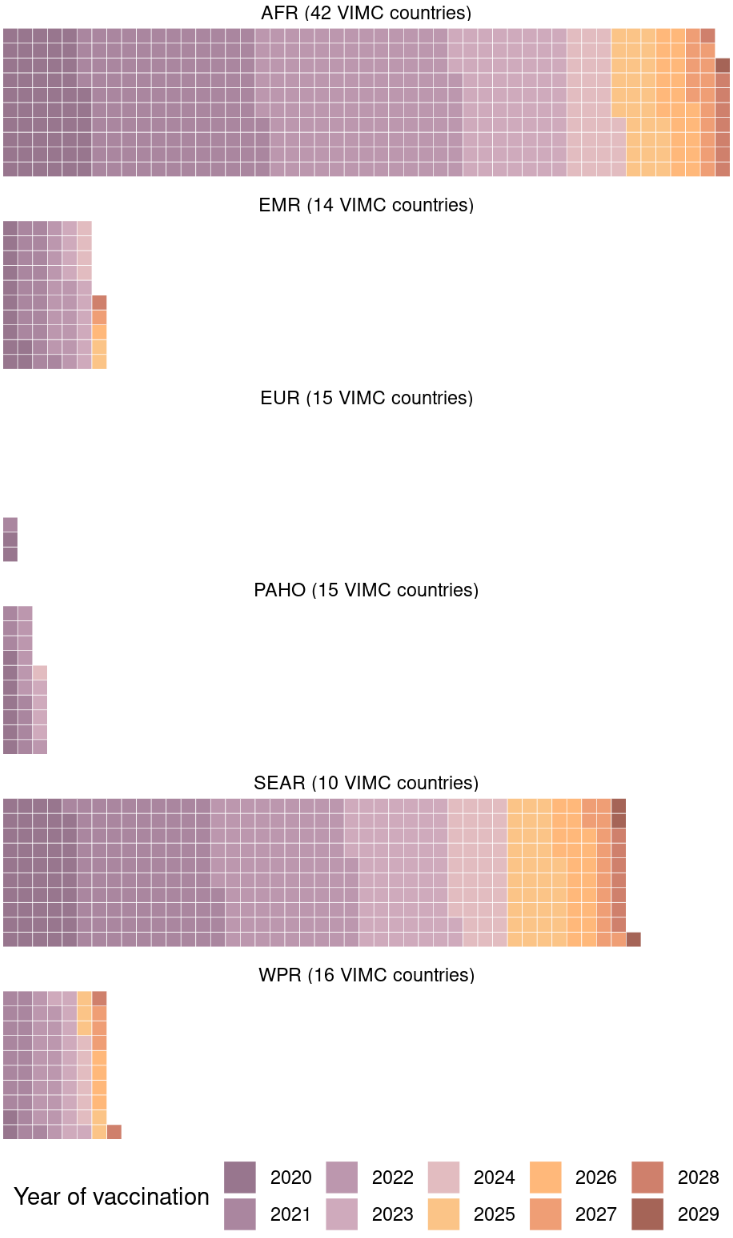

Figure B4: Mean projected numbers of additional deaths due to coverage disruption between years of vaccination 2020 and 2030 by region calculated by comparing baseline-recovery and no-disruption scenarios.

Appendix B.3. Mitigated burden due to catch-up activities

Table B8: Mitigated deaths per WHO region from calendar years 2020-2030 due to catch-up activities. Mean estimates are shown with 95%CrI shown in brackets, median estimates are shown after 'med='. *MenA was run for five countries only. There are 42 VIMC countries in AFR, 14 VIMC countries in EMR, 15 VIMC countries in EUR, 15 VIMC countries in PAHO, 10 VIMC countries in SEAR and 16 VIMC countries in WPR.

Disease	AFR	EMR	EUR	PAHO	SEAR	WPR	Total
HPV	143 (95%[116, 176]) med=141	0 (95%[0, 0]) med=0	1 (95%[0, 1]) med=1	23 (95%[20, 26]) med=23	95 (95%[53, 137]) med=96	4 (95%[2, 6]) med=4	266 (95%[206, 330]) med=265
HepB	216 (95%[181, 256]) med=217	6 (95%[2, 11]) med=5	0 (95%[0, 0]) med=0	0 (95%[0, 0]) med=0	53 (95%[36, 70]) med=53	36 (95%[27, 50]) med=35	310 (95%[260, 368]) med=311
Measles	7,148 (95%[536, 38,127]) med=2,725	1,505 (95%[441, 4,766]) med=1,099	86 (95%[-8, 265]) med=74	329 (95%[-33, 1,346]) med=197	7,013 (95%[1,997, 16,990]) med=6,168	158 (95%[-5, 951]) med=50	16,239 (95%[4,187, 57,331]) med=10,195
MenA	0 (95%[0, 0]) med=0	NA	NA	NA	NA	NA	0 (95%[0, 0]) med=0
Rubella	2 (95%[-52, 45]) med=3	-4 (95%[-322, 237]) med=0	21 (95%[-131, 215]) med=6	-18 (95%[-168, 18]) med=0	9 (95%[-19, 44]) med=12	0 (95%[-15, 16]) med=0	10 (95%[-343, 274]) med=21
YF	1,513 (95%[369, 3,244]) med=1,391	0 (95%[0, 0]) med=0	NA	-17 (95%[-79, 28]) med=-14	NA	NA	1,496 (95%[325, 3,231]) med=1,378
Total	9,022 (95%[1,706, 39,882]) med=4,819	1,508 (95%[332, 4,723]) med=1,112	108 (95%[-60, 371]) med=94	317 (95%[-105, 1,360]) med=186	7,170 (95%[2,182, 17,178]) med=6,331	197 (95%[35, 998]) med=89	18,321 (95%[6,246, 58,522]) med=12,485

Table B9: Mitigated cases per WHO region from calendar years 2020-2030 due to catch-up activities. Mean estimates are shown with 95%CrI shown in brackets, median estimates are shown after 'med='. *MenA was run for five countries only. There are 42 VIMC countries in AFR, 14 VIMC countries in EMR, 15 VIMC countries in EUR, 15 VIMC countries in PAHO, 10 VIMC countries in SEAR and 16 VIMC countries in WPR. Totals are not included across diseases as cases do not have a common definition.

Disease	AFR	EMR	EUR	PAHO	SEAR	WPR	Total
HPV	644 (95%[543, 740]) med=642	0 (95%[0, 0]) med=0	3 (95%[2, 4]) med=3	121 (95%[93, 152]) med=121	159 (95%[106, 211]) med=159	25 (95%[13, 40]) med=25	953 (95%[831, 1,094]) med=954
HepB	4,212 (95%[3,597, 4,940]) med=4,209	177 (95%[128, 229]) med=177	13 (95%[4, 28]) med=11	23 (95%[7, 37]) med=23	1,151 (95%[836, 1,434]) med=1,158	778 (95%[590, 983]) med=772	6,354 (95%[5,420, 7,395]) med=6,372
Measles	403,061 (95%[17,474, 2,029,204]) med=126,338	196,489 (95%[64,016, 541,064]) med=139,739	62,214 (95%[-21,400, 165,420]) med=56,936	54,539 (95%[-34,972, 261,774]) med=26,708	2,473,984 (95%[566,619, 5,824,381]) med=2,076,672	46,957 (95%[-12,913, 309,391]) med=12,905	3,237,244 (95%[793,977, 8,057,409]) med=2,533,648
MenA	0 (95%[0, 0]) med=0	NA	NA	NA	NA	NA	0 (95%[0, 0]) med=0
Rubella	2 (95%[-106, 107]) med=5	-6 (95%[-566, 688]) med=1	60 (95%[-282, 539]) med=19	-63 (95%[-603, 33]) med=-2	19 (95%[-45, 94]) med=26	0 (95%[-32, 34]) med=1	11 (95%[-963, 709]) med=40
YF	3,185 (95%[581, 6,632]) med=2,961	0 (95%[0, 0]) med=0	NA	-50 (95%[-153, 28]) med=-42	NA	NA	3,135 (95%[505, 6,609]) med=2,938

Table B10: Mitigated DALYs per WHO region from calendar years 2020-2030 due to catch-up activities. Mean estimates are shown with 95%CrI shown in brackets, median estimates are shown after 'med='. *MenA was run for five countries only. There are 42 VIMC countries in AFR, 14 VIMC countries in EMR, 15 VIMC countries in EUR, 15 VIMC countries in PAHO, 10 VIMC countries in SEAR and 16 VIMC countries in WPR.

Disease	AFR	EMR	EUR	PAHO	SEAR	WPR	Total
HPV	10,506 (95%[8,620, 12,819]) med=10,323	0 (95%[0, 0]) med=0	36 (95%[28, 43]) med=36	1,589 (95%[1,367, 1,805]) med=1,593	6,925 (95%[3,841, 9,995]) med=7,006	301 (95%[182, 436]) med=294	19,356 (95%[15,128, 23,846]) med=19,361
HepB	12,318 (95%[10,527, 14,381]) med=12,295	591 (95%[450, 748]) med=592	37 (95%[15, 63]) med=34	88 (95%[70, 107]) med=88	3,999 (95%[2,926, 4,941]) med=4,022	2,765 (95%[2,117, 3,472]) med=2,754	19,798 (95%[16,912, 22,991]) med=19,888
Measles	476,901 (95%[37,685, 2,448,331]) med=192,966	103,247 (95%[30,619, 319,768]) med=75,222	6,035 (95%[30, 19,472]) med=5,137	23,917 (95%[-1,653, 97,633]) med=14,219	515,105 (95%[152,488, 1,238,023]) med=448,551	11,648 (95%[-104, 68,119]) med=3,997	1,136,852 (95%[300,789, 3,935,816]) med=735,101
MenA	0 (95%[0, 0]) med=0	NA	NA	NA	NA	NA	0 (95%[0, 0]) med=0
Rubella	70 (95%[-2,973, 3,086]) med=127	-151 (95%[-14,537, 17,662]) med=30	1,629 (95%[-6,057, 14,035]) med=437	-1,476 (95%[-13,801, 707]) med=-45	435 (95%[-1,240, 2,383]) med=619	-2 (95%[-647, 674]) med=16	506 (95%[-24,253, 17,515]) med=1,012
YF	96,607 (95%[23,876, 208,038]) med=88,736	0 (95%[0, 0]) med=0	NA	-1,505 (95%[-5,998, 1,388]) med=-1,198	NA	NA	95,102 (95%[20,009, 207,079]) med=87,563
Total	596,401 (95%[105,101, 2,560,719]) med=316,749	103,687 (95%[28,058, 318,482]) med=77,619	7,736 (95%[-2,412, 23,078]) med=6,924	22,613 (95%[-5,940, 98,374]) med=12,814	526,464 (95%[161,813, 1,251,379]) med=461,640	14,711 (95%[3,186, 71,456]) med=7,251	1,271,613 (95%[428,550, 3,989,816]) med=863,855

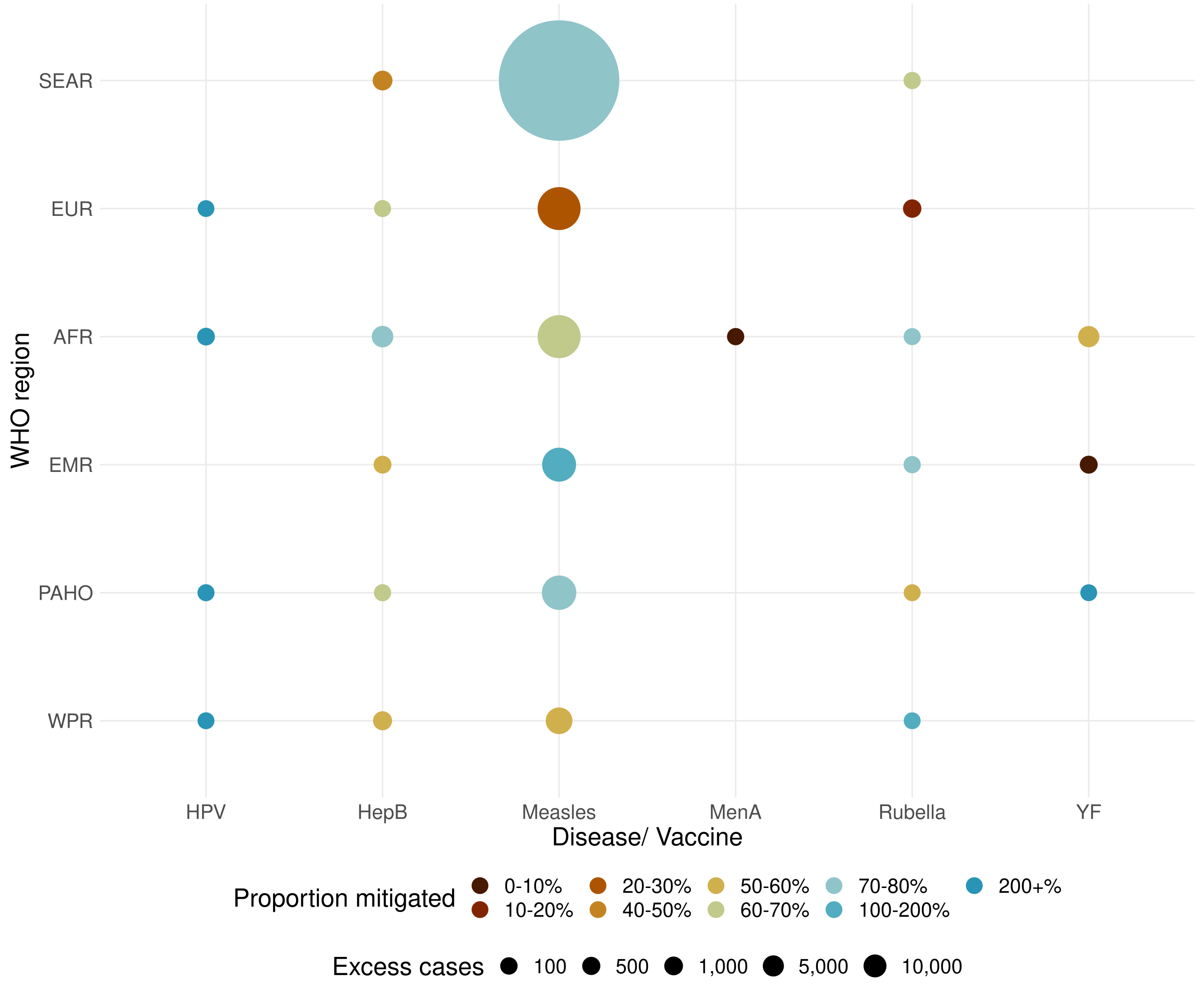

Figure B5: Excess cases between calendar years 2023-2030 due to coverage disruptions (size of circle) and proportion of those cases mitigated by catch-up activities over the decade (colour of circle).

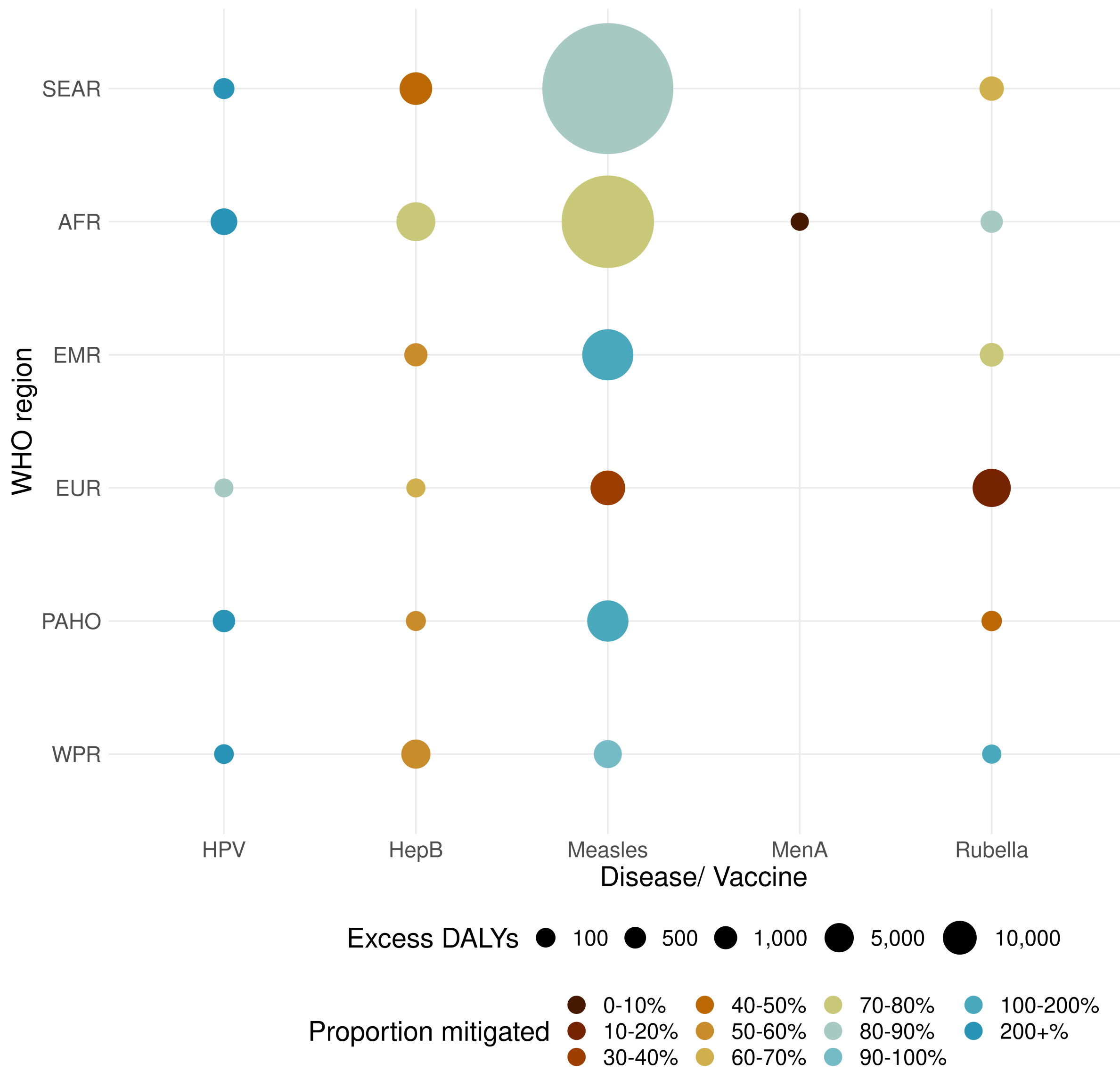

Figure B6: Excess DALYs between calendar years 2023-2030 due to coverage disruptions (size of circle) and proportion of those DALYs mitigated by catch-up activities over the decade (colour of circle).

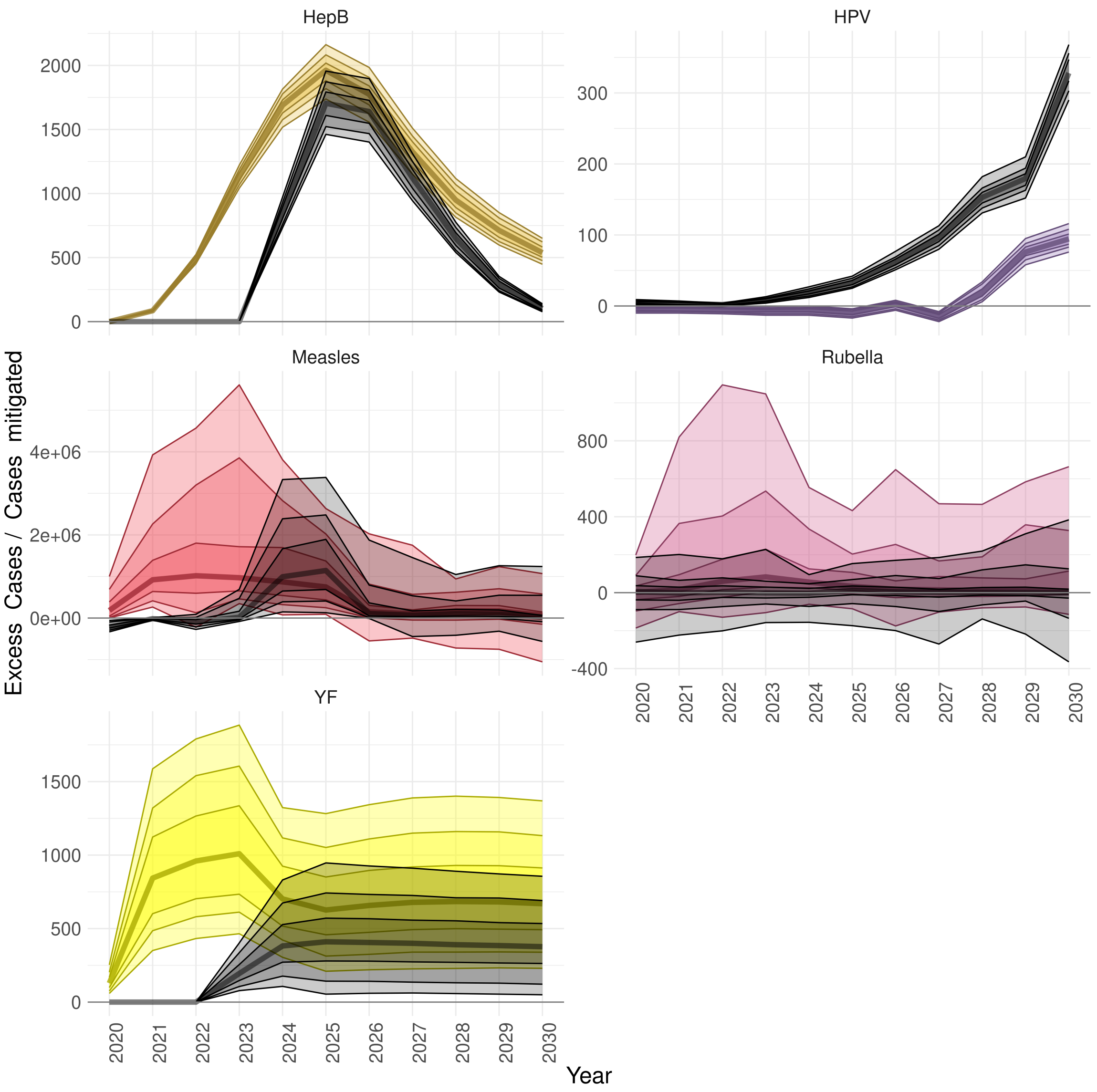

Figure B7: Excess cases by calendar year due to coverage disruptions (shown in colour) and catch-up activities (shown in greyscale). Confidence intervals for 95%, 80% and 50% levels are shown in ribbons, the median estimate is shown with a solid line. Diseases where the median excess burden does not exceed 50 in any one year are not shown.

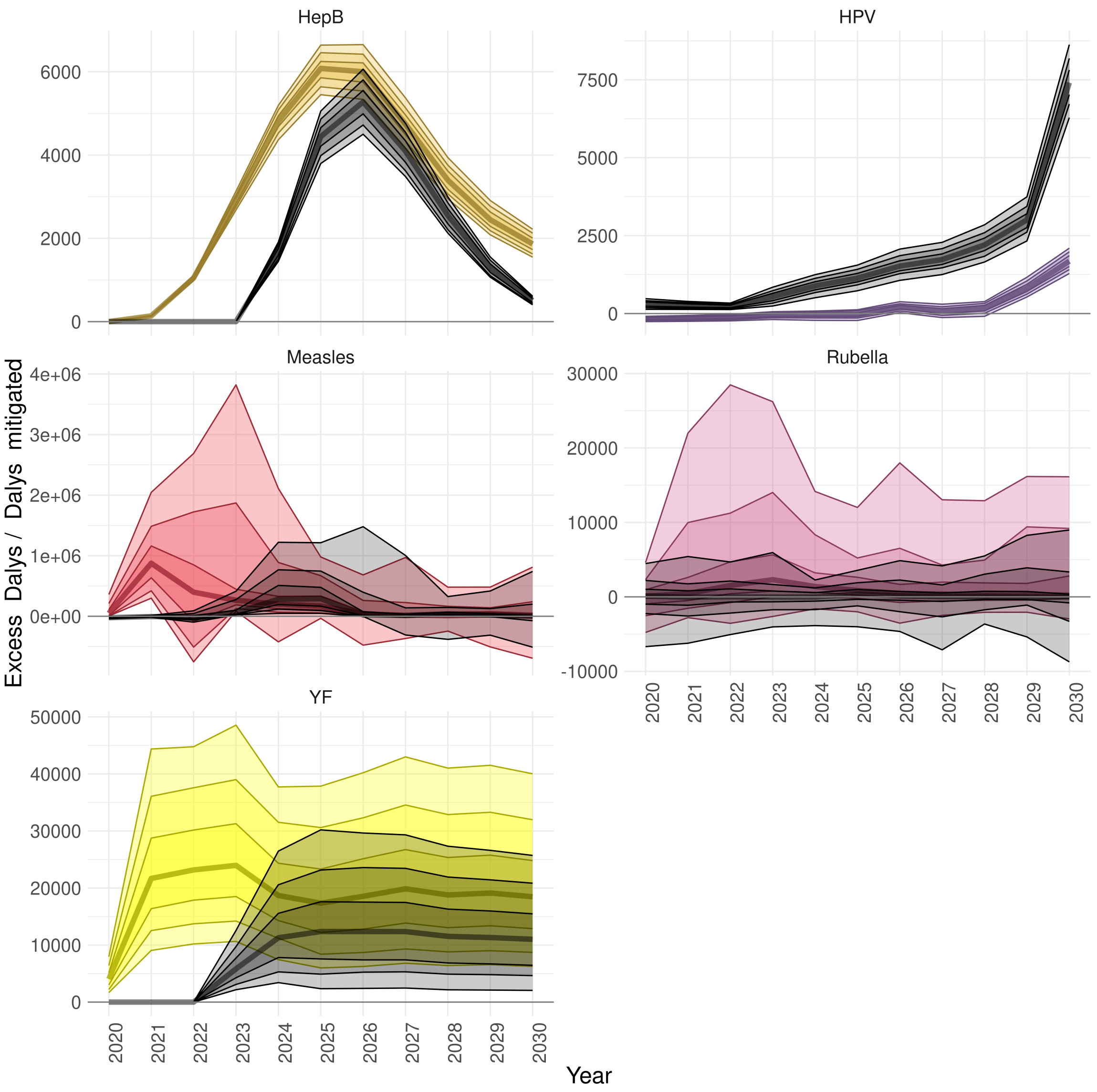

Figure B8: Excess DALYs by calendar year due to coverage disruptions (shown in colour) and catch-up activities (shown in greyscale). Confidence intervals for 95%, 80% and 50% levels are shown in ribbons, the median estimate is shown with a solid line. Diseases where the median excess burden does not exceed 50 in any one year are not shown.

Appendix B.4. Further notes on negative estimates

There are four main reasons for negative estimates of impact or excess when comparing two vaccination scenarios. In this manuscript they arise where i) non-integer rounding errors accumulate, ii) there are slight stochastic variations between scenarios, iii) projected recovery following disruption exceeds baseline projections of coverage, and iv) for excess burden, where mitigation activities are more successful at averting burden than disruption was in creating it. We will elaborate on these points with examples below.

Non-integer rounding errors and stochastic variation. These issues occur for some estimates of HPV and rubella excess burden where estimates are presented to a high degree of precision, eg. 6 decimal places, it can occur that presenting cumulative figures over multiple years, age groups, countries and vaccination activities can lead to small integer differences.

Projected recovery following disruption exceeds baseline projections of coverage. We project a nonlinear scale-up of coverage for all routine activities for all vaccines. Where we include disruption to routine coverage (eg. from WUENIC data) we scale-up following this disruption. However, we do not cap the baseline-recovery scenario by the no-disruption scenario. As such, for some country year combinations, the coverage achieved in the baseline-recovery scenario exceeds that in the no-disruption scenario. This aligns with expectations following coverage disruptions that counties may 'bounce back' either through renewed effort in existing activities or through the 'big catch up'.

Mitigation activities avert more burden than was created by disruption. To calculate the proportion of excess burden mitigated, we compare both the no-disruption and the baseline-recovery+catchup scenarios with the baseline-recovery scenario. In some country/ vaccine combinations disruption only causes a modest increase in burden within the time frame. However, catch-up activities, essentially another opportunity to increase coverage in immunisation can create large benefits which exceed the damages of disruption. This is particularly seen for HPV where the burden averted per vaccine course is extremely high, so any additional vaccination activities will have large benefits, but burden occurs later in life and so there is a wider window of opportunity to protect those cohorts missed or affected by disruption.

Appendix C. Vaccination coverage figures

Appendix C.1. Examining Coverage by Scenario

Appendix C.2. Examining Excess FVPs by Disease and WHO Region

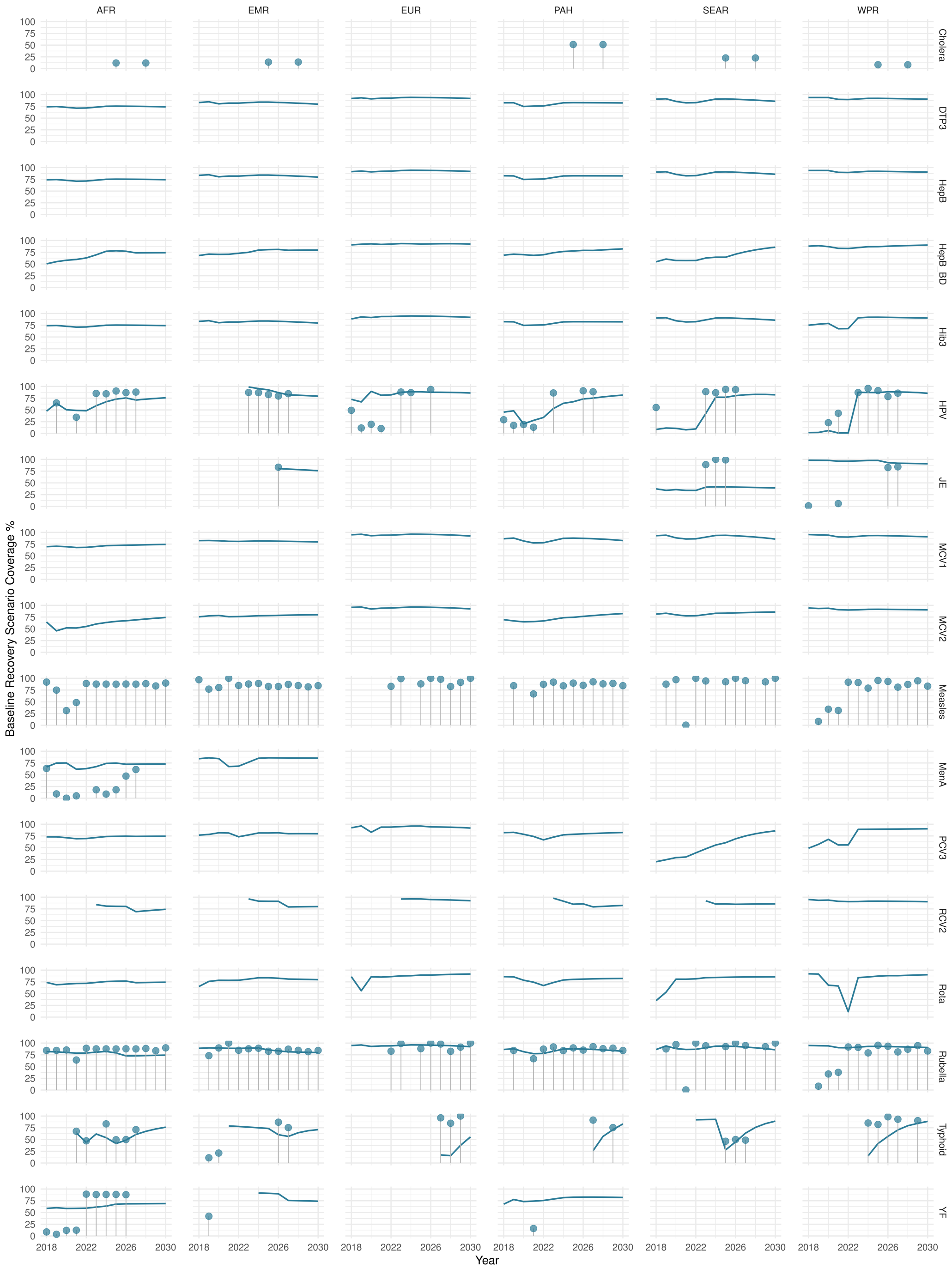

Figure C1: Coverage (%) for routine (line) and campaign (lollipops) immunization under the baseline recovery scenario between 2018 and 2030, split by WHO Region and vaccine.

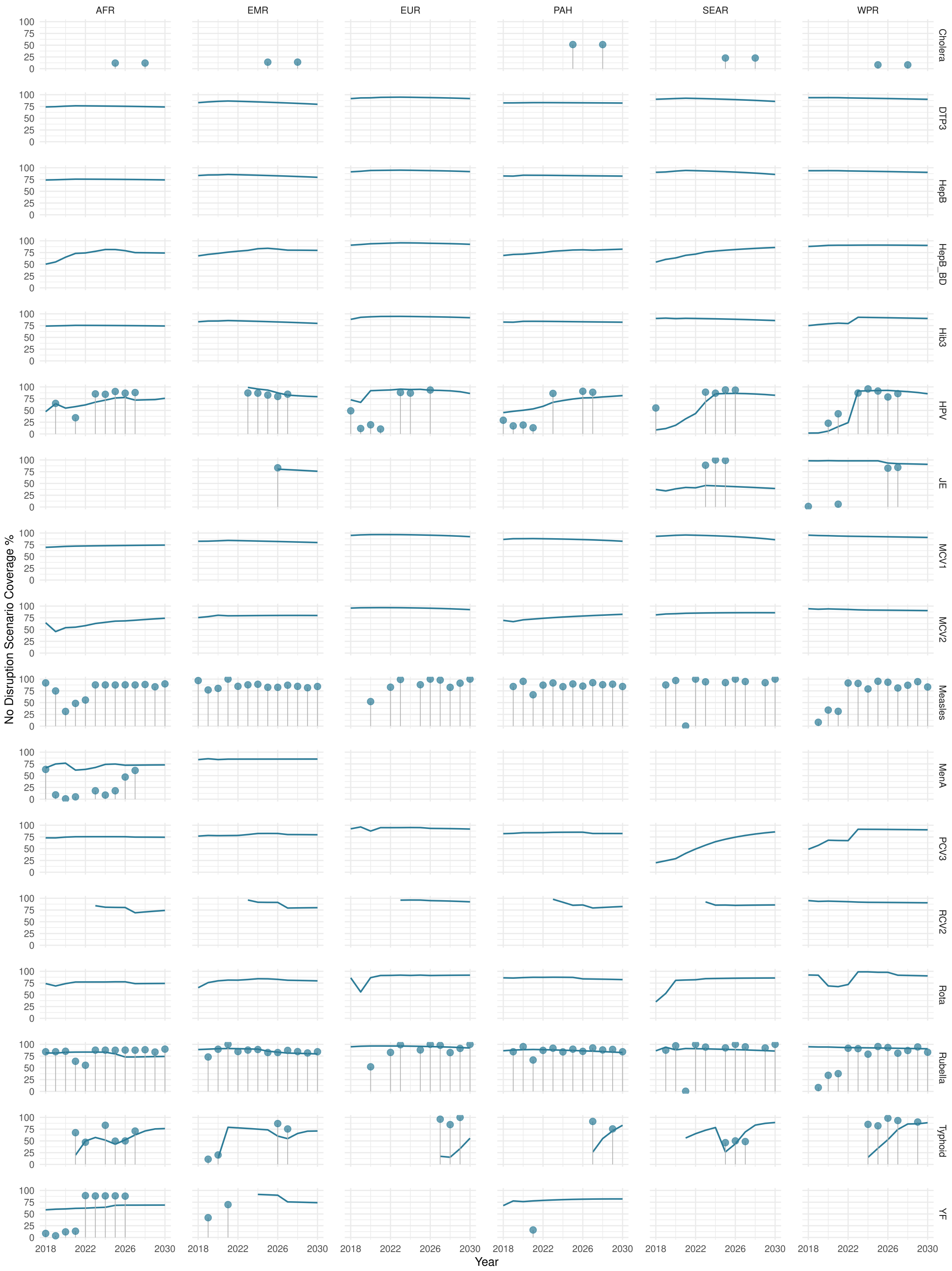

Figure C2: Coverage (%) for routine (line) and campaign (lollipops) immunization under the no disruption scenario between 2018 and 2030, split by WHO Region and vaccine.

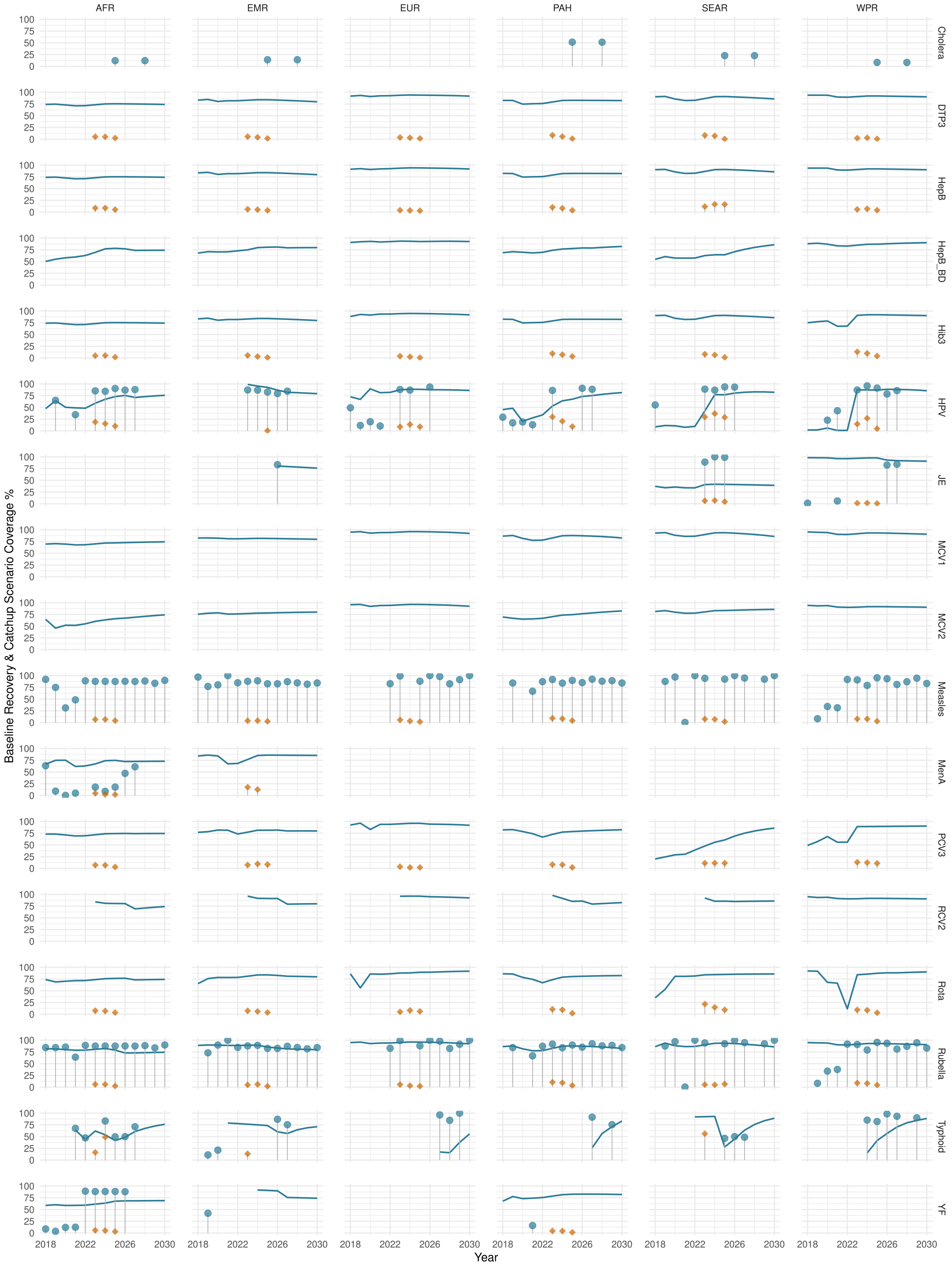

Figure C3: Coverage (%) for routine (line), campaign (blue lollipops), and routine intensified (yellow lollipops) immunization under the baseline recovery and catchup scenario between 2018 and 2030, split by WHO Region and vaccine.

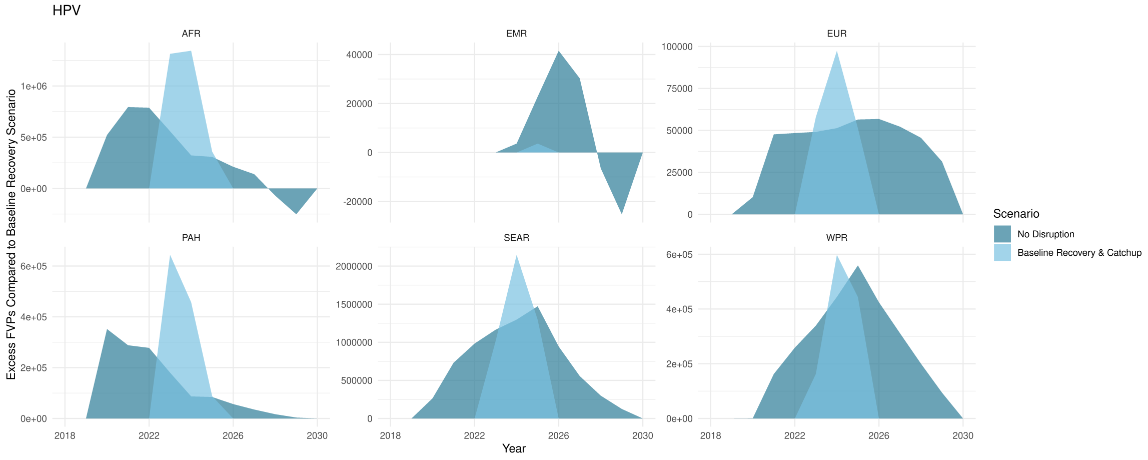

Figure C4: Additional FVPs between 2018 and 2030 for HPV routine, campaign, and routine-intensified immunisation (where applicable), under the respective scenarios as compared to the baseline recovery scenario.

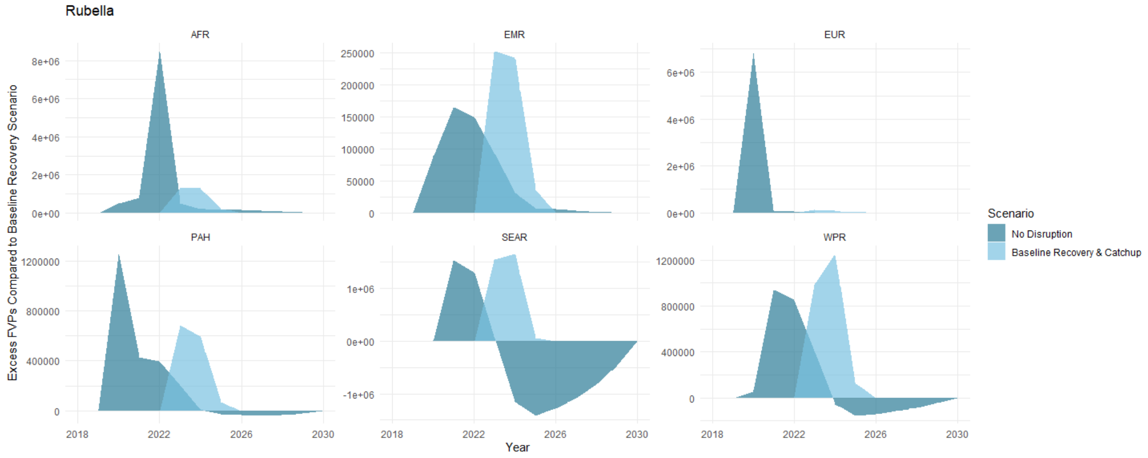

Figure C5: Additional FVPs between 2018 and 2030 for rubella routine, campaign, and routine-intensified immunisation (where applicable), under the respective scenarios as compared to the baseline recovery scenario.

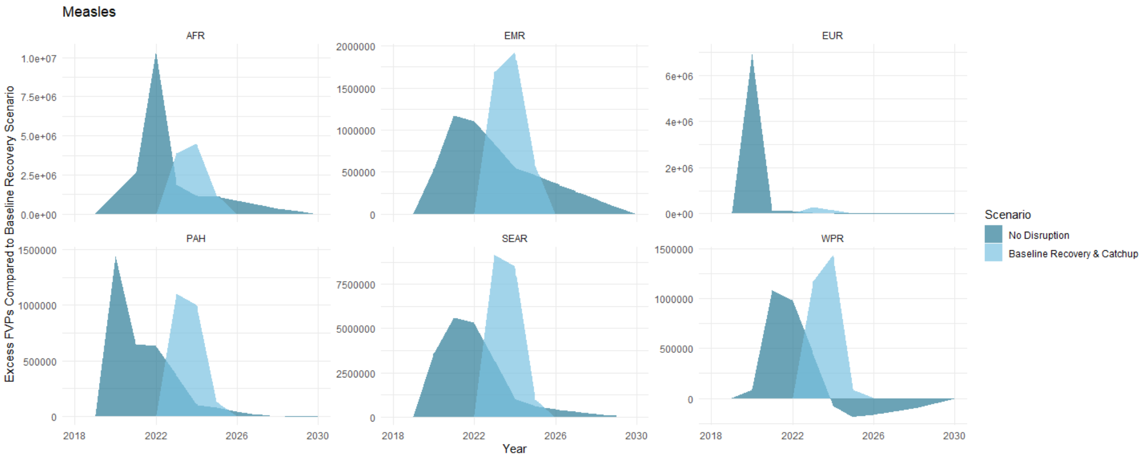

Figure C6: Additional FVPs between 2018 and 2030 for mealses routine, campaign, and routine-intensified immunisation (where applicable), under the respective scenarios as compared to the baseline recovery scenario.

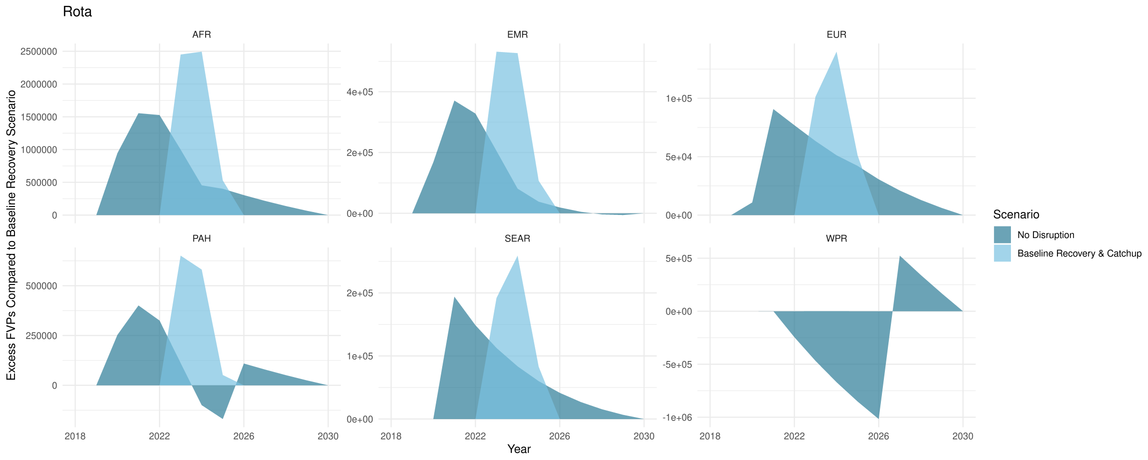

Figure C7: Additional FVPs between 2018 and 2030 for rota routine, campaign, and routine-intensified immunisation (where applicable), under the respective scenarios as compared to the baseline recovery scenario.

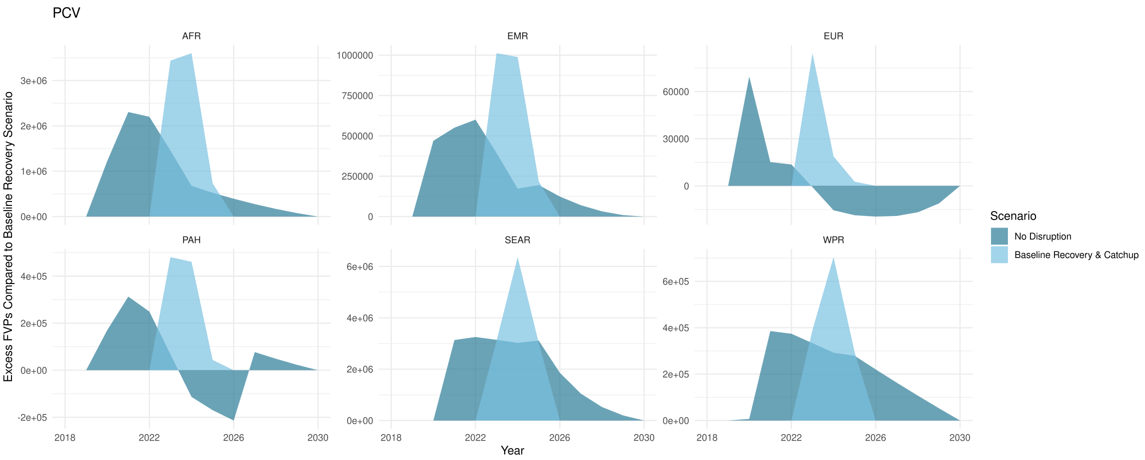

Figure C8: Additional FVPs between 2018 and 2030 for PCV routine, campaign, and routine-intensified immunisation (where applicable), under the respective scenarios as compared to the baseline recovery scenario.

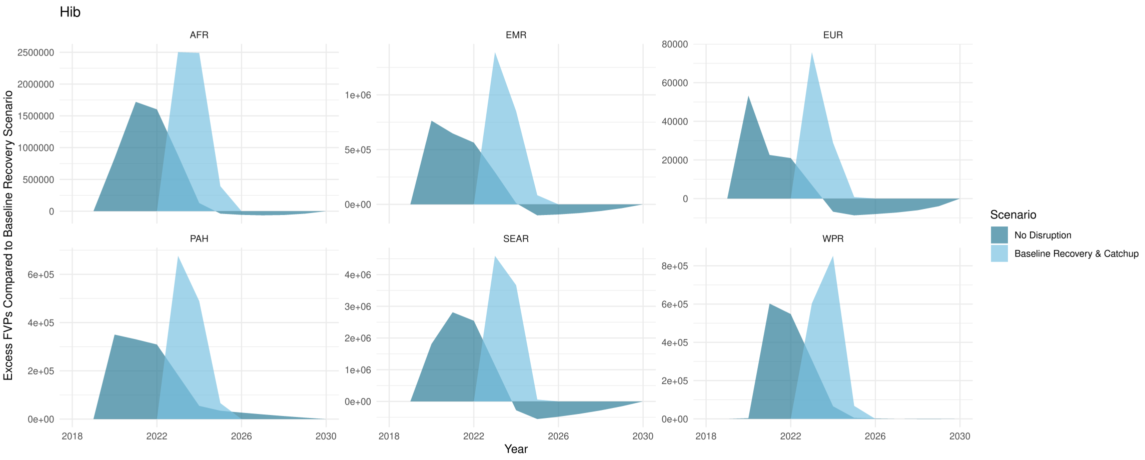

Figure C9: Additional FVPs between 2018 and 2030 for Hib routine, campaign, and routine-intensified immunisation (where applicable), under the respective scenarios as compared to the baseline recovery scenario.

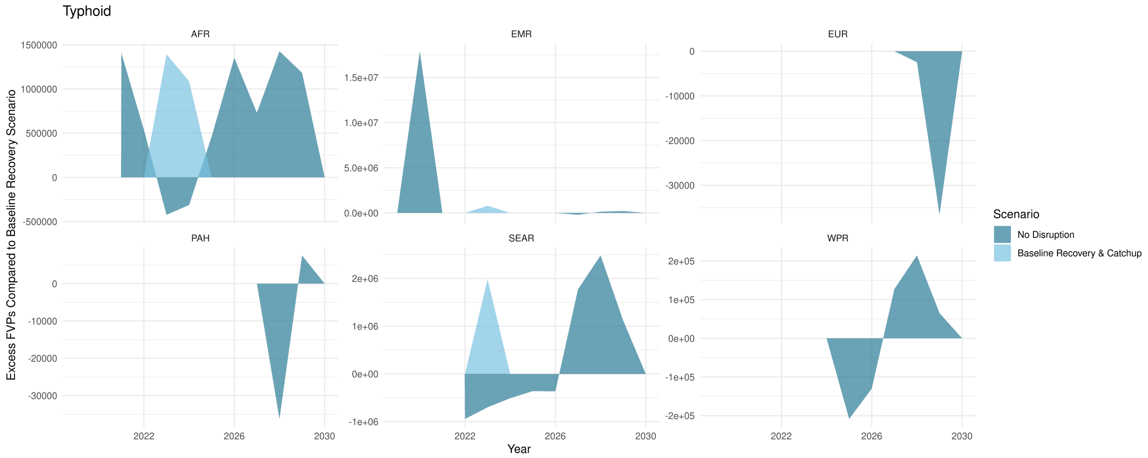

Figure C10: Additional FVPs between 2018 and 2030 for Typhoid routine, campaign, and routine-intensified immunisation (where applicable), under the respective scenarios as compared to the baseline recovery scenario.

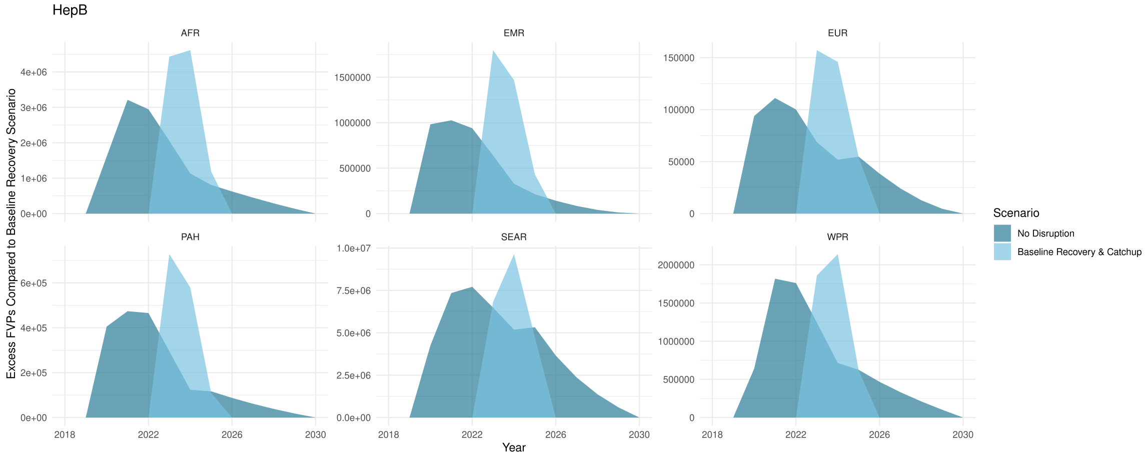

Figure C11: Additional FVPs between 2018 and 2030 for HepB routine, campaign, and routine-intensified immunisation (where applicable), under the respective scenarios as compared to the baseline recovery scenario.

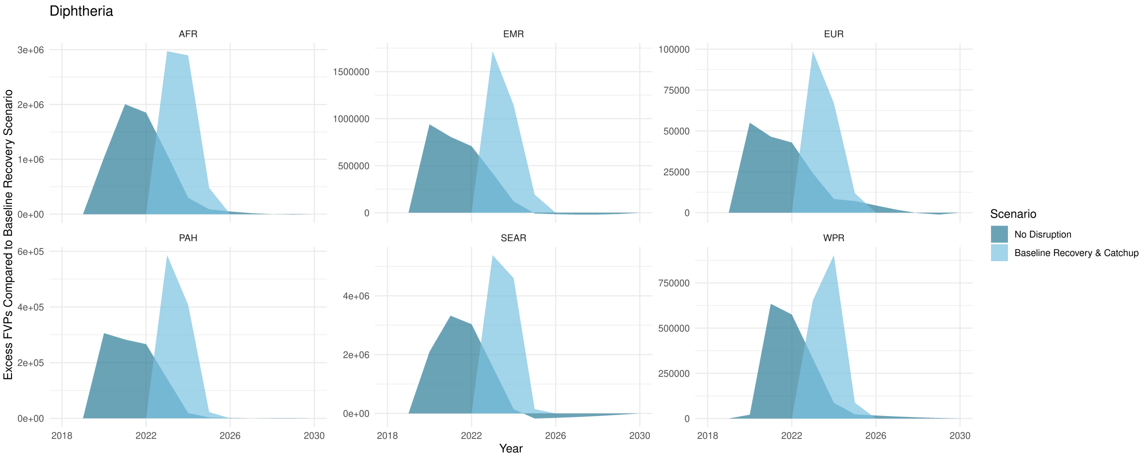

Figure C12: Additional FVPs between 2018 and 2030 for Diptheria routine, campaign, and routine-intensified immunisation (where applicable), under the respective scenarios as compared to the baseline recovery scenario.

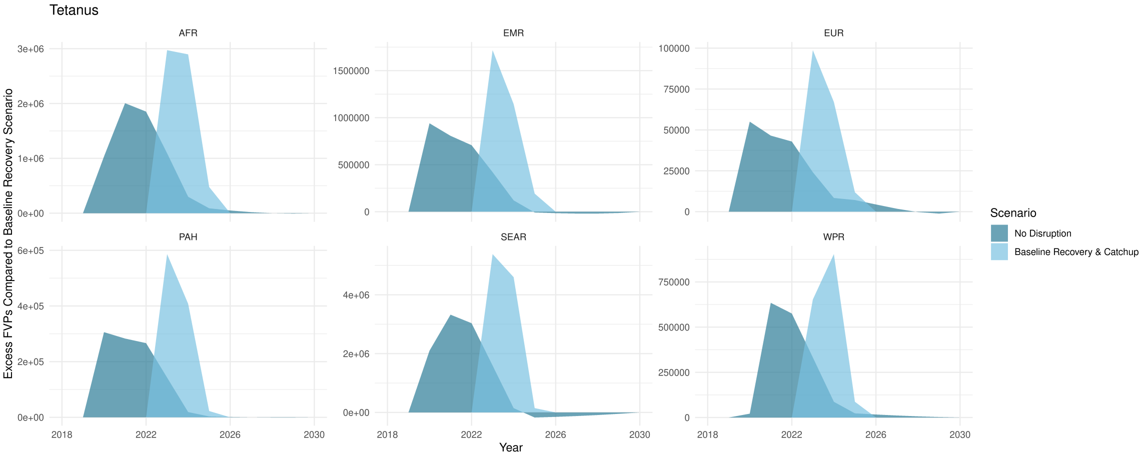

Figure C13: Additional FVPs between 2018 and 2030 for Tetanus routine, campaign, and routine-intensified immunisation (where applicable), under the respective scenarios as compared to the baseline recovery scenario.

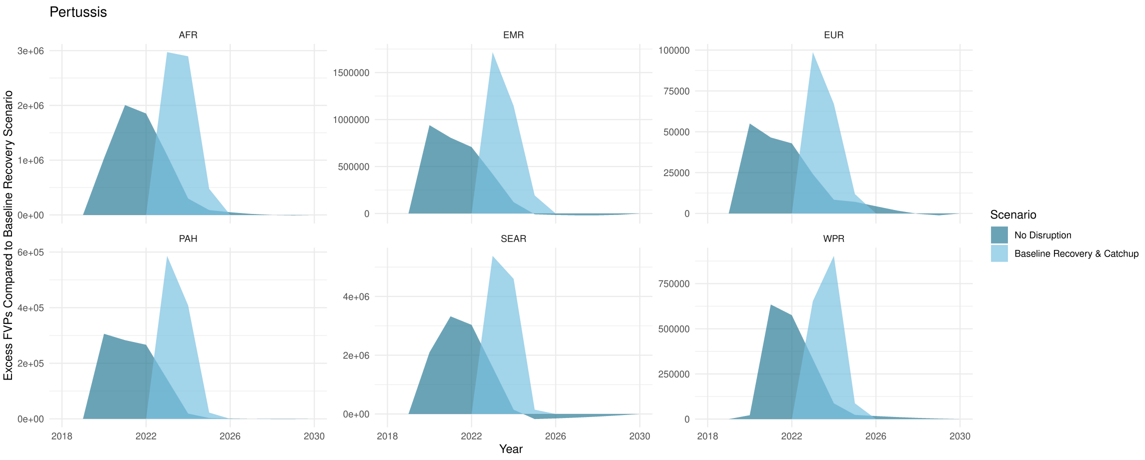

Figure C14: Additional FVPs between 2018 and 2030 for Pertussis routine, campaign, and routine-intensified immunisation (where applicable), under the respective scenarios as compared to the baseline recovery scenario.

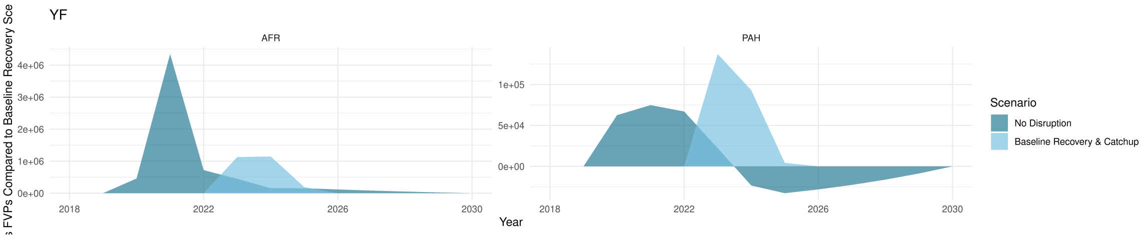

Figure C15: Additional FVPs between 2018 and 2030 for yellow fever routine, campaign, and routine-intensified immunisation (where applicable), under the respective scenarios as compared to the baseline recovery scenario.

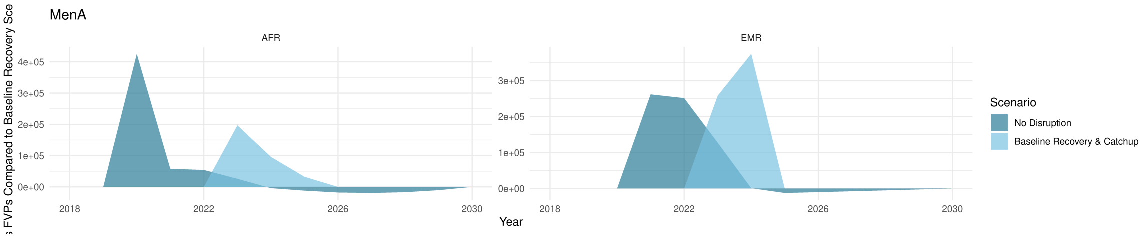

Figure C16: Additional FVPs between 2018 and 2030 for MenA routine, campaign, and routine-intensified immunisation (where applicable), under the respective scenarios as compared to the baseline recovery scenario.

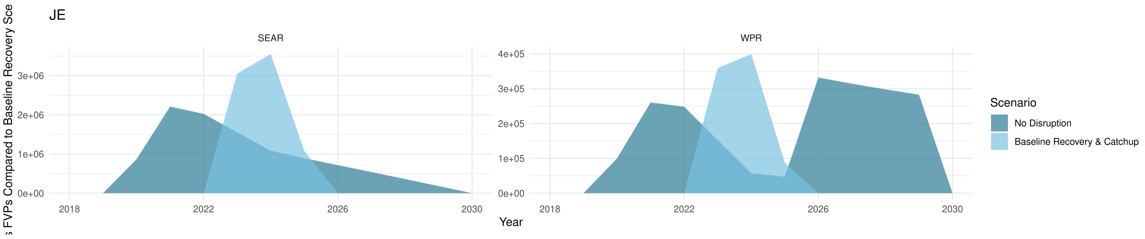

Figure C17: Additional FVPs between 2018 and 2030 for JE routine, campaign, and routine-intensified immunisation (where applicable), under the respective scenarios as compared to the baseline recovery scenario.

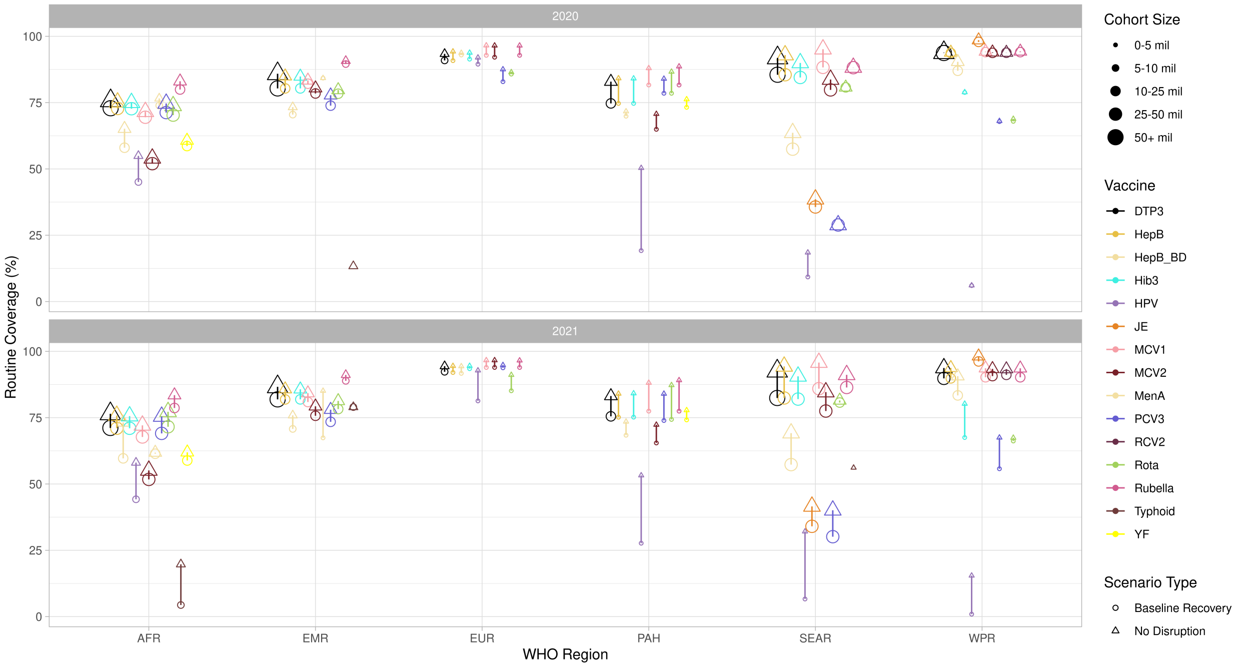

Figure C18: Difference in adjusted routine immunisation coverage (%) between the no disruption and baseline recovery scenario for 2020 and 2021. Data is split by vaccine and WHO Region.

Appendix D. Model descriptions

Appendix D.1. Cholera – Johns Hopkins Bloomberg School of Public Health

This phenomenological, spatial model assumes that the mean annual incidence rate of suspected cholera in a given location represents a baseline cholera risk, and that cholera incidence may be projected by considering direct and indirect vaccine effects and waning effectiveness over time, population size changes and spatial turnover, and secular trends in country-level cholera incidence rates. The model is based a previously published cholera vaccine impact model [7].

For the 35 modeled countries in sub-Saharan Africa, the baseline spatial cholera risk was the estimated mean annual suspected cholera incidence rate from 2010-2016 down-scaled from the 20 km by 20 km to 5 km by 5 km grid cell scale [8]. Baseline spatial cholera risk for Bangladesh was derived from unpublished estimates of the mean annual clinical cholera incidence rate in 5 km by 5 km grid cells across Bangladesh from 2014-2018 using a Bayesian hierarchical model similar to that in [8]. Acute watery diarrhea (AWD) cases were acquired from the Directorate General Health Services (DGHS) of Bangladesh from participating public and private hospitals in all 64 districts, and culture-confirmed V. cholerae surveillance data were obtained from systematic sampling of AWD patients at 22 sentinel hospital sites that represent an enteric disease surveillance system. We applied the national mean annual incidence rate in Bangladesh to 5 km by 5 km grid cells in India in a population-weighted manner. For the 10 remaining modeled countries, annual country-level reports of cholera to WHO [8] were used to estimate mean annual cholera incidence rates, and the rate was assumed to apply homogeneously to all 5 km by 5 km cells in the country.

Vaccine doses were administered to second-level administrative units (i.e., districts) with the greatest cholera-affected population sizes. Affected population size was defined as the product of the proportion of the country population living in the district, as calculated from 2020 WorldPop population estimates and GADM district shapefiles [9, 10], and the mean annual incidence rate across all 5 km by 5 km cells in the district. We assumed that vaccination campaign coverage was 80% and that a district could not receive vaccines more than once every three years.

As in [7], indirect vaccine protection was modeled with a logistic function fit to the relative reduction in incidence among unvaccinated individuals and OCV coverage in a 5 km by 5 km grid cell, based on data from vaccine trials in India and Bangladesh [11, 12]. Also as in [7], waning vaccine efficacy was obtained by fitting a log-linear decay function to 2-dose vaccine efficacy reported 0 to 5 years after vaccination according to a previously published meta-analysis [13].

Model uncertainty in the 200 stochastic simulations is derived from different posterior draws for baseline cholera risk, and four simulation settings that cross two dimensions –

temporal incidence rate trend and stochastic outbreak variability – with two options each (i.e., 50 simulations per setting).

In one dimension, we consider a constant or modeled temporal incidence rate trend from 2000 to 2100, the VIMC projection period. The constant trend assumes that baseline spatial cholera risk is constant over the 100-year period. The modeled trend fits a log-linear regression model with country-level group effects to all available annual country-level reports of cholera to WHO [14]. The model fit was then used to calculate a multiplier for each projection year relative to the 2014 reference year, and the multiplier was then used to project the country's baseline spatial cholera risk forwards and backwards from 2014.

The second dimension considered sub-Saharan African countries did or did not have stochastic outbreak variability. Using published data on suspected cholera outbreaks in sub-Saharan Africa from 2010 to 2020 [15], we calculated the annual probability of an outbreak and annualized multipliers for outbreak and non-outbreak attack rates relative to the baseline cholera risk in each second-level administrative unit. For each simulation with outbreak variability, the model performed stochastic draws over a single 10-year period to determine which years a given district had an outbreak. This same 10-year pattern was recycled over the 101-year projection period (e.g., 2000-2009, 2010-2019, etc had the same pattern). Outbreak and non-outbreak multipliers were then multiplied with the baseline spatial cholera risk accordingly. Only 21 African countries had stochastic outbreak variability in our model.

The cholera vaccine impact model is limited in that it does not explicitly model immunity due to natural infection. Additionally, temporal variation in baseline cholera risk is considered only in countries and simulations that include stochastic outbreak variability even though cholera transmission can vary substantially from year to year.

The model package `ocvImpact` and scripts may be be found on Github at https://github.com/HopkinsIDD

Appendix D.2. Cholera and typhoid fever - International Vaccine Institute (IVI)

IVI model is a static and age-structured model. It calculates the expected number of cases by multiplying UN-projected population size with incidence rate per Capita for each country for 1-year age group for each year from 2000 to 2100. This is to assume that the incidence rate per Capita for each of the countries accounts for population-level of immunity and the occurrence of cases in one year does not affect occurrences in the subsequent years. The expected number of deaths is calculated by multiplying case fatality ratio and similarly, the disability-adjusted life years (DALYs) is calculated by multiplying disability weights and duration of illness.

Incidence rates are based on the existing estimates for a particular year (e.g., 2010 for cholera or 2017 for typhoid fever) and incidence rates for other years are projected using the information on the proportion of population with access to improved water, sanitation,

and hygiene (WASH) and relative risk depending on the WASH status as in the previous study [16]. We used pooled odds ratios based on case-control studies of WASH and cholera [17] and typhoid fever [18] to approximate the relative risk. The proportion of high- and low-risk groups change over time because of the improvement in WASH and this change leads to change in the total incidence while subpopulation-specific incidence rates remain constant. To project the incidence rate into the period (2000-2100), we used the data on the percentage of the population who have access to the improved WASH from the WHO/UNICEF Joint Monitoring Programme for Water Supply, Sanitation, and Hygiene (JMP). We used the data from 2000 to 2020 to fit the trend and project it into the future.

We assumed a so-called all-or-nothing vaccine, which leads to that a fraction VE of the vaccine recipients are completely protected from infection. The fraction VE represents a vaccine efficacy and is modelled as a function of time (i.e., year post-vaccination) (also by age of the vaccine recipients in case of cholera).

For simulation, we sampled model parameters using Sabol's low-discrepancy sequence generator implemented in R package pomp [19, 20] as uniform random variables ranging from 0 to 1 and transformed to appropriate distributions (such as beta, lognormal, or uniform with different bounds). Two hundred samples of parameter sets were simulated for each country. To determine the parameters for the parametric distributions, we used the method provided in the study [21]. For a given 95% confidence interval with upper (UB) and lower bounds (LB), the mean is assumed to be point estimate and standard deviation was calculated as (UB - LB)/2/1.96.

Appendix D.2.1. Cholera

Incidence rates are based on the study by Lessler et al. [8] for 35 target countries of sub-Saharan Africa: Angola, Burundi, Benin, Burkina Faso, Central African Republic, Côte d'Ivoire, Cameroon, the Democratic Republic of the Congo, Republic of the Congo, Ethiopia, Ghana, Guinea, Guinea-Bissau, Kenya, Liberia, Madagascar, Mali, Mozambique, Mauritania, Malawi, Namibia, Niger, Nigeria, Rwanda, Senegal, Sierra Leone, Somalia, South Sudan, Chad, Togo, Tanzania, Uganda, South Africa, Zambia, and Zimbabwe. The study provides 20 km × 20 km grid-level incidence and we summed across grids that comprise a country and divided by the UN-projected population size in 2010 to the county-level incidence rate for the country. Incidence rates for the 10 countries outside of sub-Saharan Africa (Afghanistan, Algeria, Haiti, Iran, Iraq, Nepal, Pakistan, Philippines, Thailand, and Yemen) were based on WHO Global Health Observatory [22] or public health department of a government (e.g., Ministere Sante Publique et de La Population of Haiti). India and Bangladesh were assumed to have the same incidence rate, 160 per 100,000 person-years, based on the observation from Kolkata, India from 1 May 2003 to 30 April 2005 [23]. We modelled the incidence rate for each country is a random variable following a lognormal distribution of which the mean is the point estimate and standard deviation is 1.5 times the mean roughly based on variations in WHO data [8]. Case fatality ratios were calcu-

lated based on the data from the WHO Global Health Observatory [22]. We used only the observations since 2000 to reflect recent findings.

Vaccine efficacy at year t post-vaccination was determined based on the meta-analyses of seven trials (with 695 patients with cholera) and six observational studies (217 patients with cholera) [14]. Indirect effect of the vaccine was implemented as a function of vaccine coverage in the population. The magnitude of indirect effect for a given vaccine coverage was based on the cluster-randomized clinical trials in Matlab, Bangladesh [12] and Kolkata, India [13]. These data provide incidence rates of unvaccinated people in the vaccine clusters with differing vaccine coverage rates. We fit a logistic function in which the indirect vaccine efficacy is the dependent variable and the effective vaccine coverage of the cluster, defined as the direct vaccine efficacy multiplied by the vaccine coverage rate, as the explanatory variable as in [7]. We do not make any explicit assumptions on the natural immunity.

Appendix D.2.2. Typhoid fever

Target countries are Afghanistan, Angola, Albania, Armenia, Azerbaijan, Burundi, Benin, Burkina Faso, Bangladesh, Belize, Bolivia, Bhutan, Central African Republic, Cote d'Ivoire, Cameroon, Congo, Congo, Comoros, Cabo Verde, Cuba, Djibouti, Eritrea, Ethiopia, Fiji, Micronesia, Federated States of, Georgia, Ghana, Guinea, Gambia, Guinea-Bissau, Guatemala, Guyana, Honduras, Haiti, Indonesia, India, Iran, Kenya, Kyrgyzstan, Cambodia, Kiribati, Lao People's Democratic Republic, Liberia, Sri Lanka, Lesotho, Morocco, Moldova, Republic of, Madagascar, Mali, Myanmar, Mongolia, Mozambique, Mauritania, Malawi, Namibia, Niger, Nigeria, Nicaragua, Nepal, Pakistan, Peru, Philippines, Papua New Guinea, Korea, Democratic People's Republic of, Paraguay, Rwanda, Sudan, Senegal, Solomon Islands, Sierra Leone, El Salvador, Somalia, South Sudan, Sao Tome and Principe, Swaziland, Chad, Togo, Thailand, Tajikistan, Timor-Leste, Tonga, Tanzania, United Republic of, Uganda, Ukraine, Uzbekistan, Venezuela, Bolivarian Republic of, Viet Nam, Vanuatu, Samoa, Yemen, South Africa, Zambia, and Zimbabwe. We assumed that country-level incidence rates are uniformly-distributed between the minimum and the maximum of incidence rate estimates from previous studies [24, 25, 26, 27]. Case fatality ratio varies by region and age (children vs. adults) based on the meta-analysis [28].

We divided typhoid fever into 4 classes according to symptoms: moderate typhoid fever, severe typhoid fever, severe typhoid fever with gastrointestinal bleeding, and severe typhoid fever with abdominal complications (other than gastrointestinal bleeding) following the previous study [27].

Vaccine efficacy is based on the total vaccine effectiveness of the Vi-tetanus toxoid conjugate vaccine (Vi-TT) observed in Bangladesh [29]. Estimates from clinical trials indicates varying vaccine effectiveness [29, 30, 31] and we assumed that total protective efficacy [29] was the most relevant for the simulation as it compares the vaccine recipients in the vaccinated population and the people from the unvaccinated population. We assumed that

vaccination provides indirect effect [29]. The study showed that indirect effect was suggestive and not statistically significant at the 5% significance level. However, we believed that the indirect effects of the vaccine would be natural considering the fecal-oral transmission of typhoid fever and it was shown to be present in another fecal-oral transmission of cholera [12, 13]. Total and indirect vaccine efficacy, case fatality ratio, the proportion and disability weights for different classes of typhoid fever, and duration of illness in term of year were assumed to follow a Beta distribution.

Appendix D.3. Hib and Rotavirus – London School of Hygiene and Tropical Medicine (LSHTM)

UNIVAC (universal vaccine decision support model) is a static cohort model with a finely disaggregated age structure (weeks of age ¡5 years, single years of age 5–99 years). A detailed description of the model and the methods for estimating vaccine impact are available in [32]. UNIVAC is available as an R script for desk-based multi-country analyses. It is also available as an Excel-based decision-support model, where it has been widely used by national Ministries of Health in low and middle income countries (LMICs) to estimate the potential impact, cost-effectiveness and benefit-risk of alternative vaccine policy options. In the context of the vaccine impact modelling consortium (VIMC), the R version of UNIVAC was used to generate transparent desk-based estimates of the impact (% reduction in cases, clinic visits, hospitalisations, lifelong sequelae, deaths and DALYs) of two vaccines (haemophilus influenza type b – Hib, and rotavirus) over the period 2000–2030 in 112 LMICs.

Interpolated 1 year time and age estimates [33] were used to calculate the number of life-years between birth and birth and 5 years of age for each of the 31 births cohorts (2000–2030) in each of the 112 countries. Life-years <5 yrs were multiplied by rates of disease cases and deaths (per 100,000 aged <5 yrs) to estimate numbers of cases and deaths expected to occur without vaccination between birth and 5 years of age. The rates of disease cases and deaths due to Hib were based on estimates generated by [34] for the year 2015. For Hib, these included estimates for non-severe Hib pneumonia, severe Hib pneumonia, Hib meningitis and Hib non-pneumonia/non-meningitis (NPNM) in children aged <5 years. The risk of meningitis sequelae was based on a systematic review and meta-analysis by [35]. For rotavirus, country-specific estimates of rotavirus deaths <5 years were based on the mean of three independent sources of international burden estimates, recently compared in [36]. Estimates of rotavirus disease cases (non-severe and severe) were based on systematic reviews and meta-analyses by [37] and [38]. Granular rotavirus disease age distributions (by week of age <5 years) were based on a recent systematic review and statistical analyses by [39].

Historical time-series estimates of diarrhoea deaths have declined in the absence of vaccination [40]. To avoid over-stating the impact of vaccination, we assume the disease-specific mortality rate will decrease without vaccination at the same rate as the overall

under-five mortality rate [33]. For consistency, we make the same assumption for Hib. We do not assume any decline in the incidence of disease cases, so case fatality ratios (CFRs) decline in each successive year.

Life expectancy estimates by age and year [33] were used to calculate YLLs (years of life lost due to premature mortality) from the age/year of disease death. YLDs (years of life with disease) were calculated by multiplying disability weights by the average duration of illness. DALYs (YLLs + YLDs) were attributed to the year of disease onset.

For all three vaccines, estimates of vaccination impact were restricted to children aged <5 years. The impact was calculated by multiplying the expected number of disease events (cases, clinic visits, hospitalisations, deaths) in each week of age <5 years, by the expected coverage of vaccination in each week of age (adjusted for realistic vaccine delays/timeliness) and the expected efficacy of vaccination in each week of age (adjusted for the waning vaccine protection). The model accounted for partial vaccination by calculating the incremental impact of each dose of vaccination in each week of age. Rotavirus was modelled as a two-dose vaccine co-administered with DTP1 and DTP2 without age restrictions. Hib vaccine was modelled as a three-dose vaccine co-administered with DTP1, 2 and 3. For each vaccine, coverage projections by country and year were provided by Gavi, the Vaccine Alliance, over the period 2000–2030. Estimates of the timeliness of vaccination (coverage by week of age) were based on the timeliness of DTP1, 2 and 3 reported in USAID Demographic and Health Surveys (DHS) [41] and Multiple Cluster Indicator Surveys (MICS) [42]. Methods for estimating vaccine timeliness have been described previously in [43] . For Hib vaccination, dose-specific efficacy was based on a global systematic review and meta-analysis of RCTs by [44]. For rotavirus, vaccine efficacy by dose and duration of follow-up (year 1 and year 2) was based on a Bayesian meta-regression of RCTs by [45].

UNIVAC is not a transmission dynamic model, and thus excludes indirect effects (both positive and negative). This is likely to lead to substantial under-estimates of impact in some countries, particularly for Hib vaccine. More detailed validation against real-world post-introduction evidence of impact is needed. However, the available data in many of the countries included in this desk-based analysis are insufficient to allow validation of modelled estimates (against real-world estimates of post-introduction vaccine impact) and/or parameterisation of a country-specific transmission dynamic model. As such, there is a good deal of uncertainty in the predicted estimates for many countries.

Appendix D.4. Hib, PCV and Rota – Johns Hopkins University

The Lives Saved Tool (LiST) is a deterministic linear mathematical model for estimating the health impact of changes in health intervention coverage in low- and middle-income countries (LMICs) described in Walker, Tam, and Friberg [38]. LiST is a publicly available module within the Spectrum suite, a policy modelling system comprised of several software components. LiST contains over 80 health interventions, including vaccines, and has been used for over a decade to assist in public health decision-making and program

evaluation. Evidence-based interventions included in the model have been demonstrated to reduce stillbirths, neonatal deaths, deaths among children aged 1-59 months, maternal mortality or risk factors.

The model describes fixed relationships between inputs (intervention coverage) and outputs (cause-specific mortality or risk factor prevalence) specified in terms of the effectiveness of the intervention for reducing the probability of that outcome under the assumptions that 1) country-specific mortality rates and cause of death structure will not change dynamically, 2) changes in mortality occur in response to changes in intervention coverage, and 3) distal factors, such as improvements in wealth, affect mortality by increasing intervention coverage or reducing risk factors.

The model is built on an underlying demographic projection derived from the United Nations Population Division (UNPD) and age structure for children (0-1, 1-5, 6-11, 12-23, 24-59 months) which serves as a theoretical cohort. Each model uses country-specific inputs of demographic growth [33], under-five mortality rates [33], and cause of death structure [46, 40]. Together, these values are used to calculate cause-specific mortality and the potential deaths averted by increasing coverage of interventions. LiST attributes lives saved to changes in coverage of specific interventions, attributing impact first to preventative and then curative interventions, ordered sequentially from periconception, through pregnancy, delivery, followed by the specific age group. By using cause-specific efficacy and applying each intervention to the residual deaths remaining after the previous intervention, LiST ensures that double counting is avoided, and the potential impact of multiple interventions is not erroneously inflated.

Estimates of intervention efficacy are derived from existing reviews, many of which were published in five journal supplements [47, 38, 48, 49]. National and subnational level impact estimates modelled by LiST have been validated against measured mortality reduction in various LMIC settings and for various packages of interventions [50, 51, 52, 53, 54, 55].

LiST is used to generate estimates of cases and deaths averted for 0, 1, 2, 3 and 4 years of age due to coverage scale-up of pneumococcal conjugate vaccines (PCV), Hemophilus influenza type b (Hib) vaccine, and rotavirus vaccines. Deaths and cases are calculated separately in LiST. Incidence of diseases (number of cases per child per year) is used instead of cause-specific mortality as a baseline input to calculate cases. Cases and deaths averted by vaccination were calculated by applying estimates of scale-up in coverage in each of the countries. The model accounts for impact of other interventions in each country that could lower the risk of pneumonia, meningitis, or diarrhoea incidence (e.g. clean water and sanitation), or reduce mortality from pneumonia, meningitis, or diarrhoea (e.g. antibiotic treatment) using country-specific coverage of interventions drawing primarily on data from the Demographic and Health Survey (dhsprogram.com) and/or Multiple Indicator Cluster Survey (mics.unicef.org). The specific associations between interventions, risk factors, and mortality within LiST can be accessed via an interactive tool (LiSTVisualizer.org). Deaths

and cases averted were calculated holding coverage of all other interventions constant.

The LiST uncertainty bounds are produced using a Monte Carlo approach. For each of the key assumptions in the model we have developed distributions around those values. These include efficacy of interventions, mortality rates, causes of death, relative risks of risk factor for mortality and incidence for severe pneumonia, meningitis and diarrhoea. In general, beta distributions were used for effectiveness of interventions, correlated normal distribution for mortality rates, Dirichlet distribution for death causes, and log-normal distribution for relative risks. Further information regarding rationale for sampling distributions can be provided upon request.

For the estimates presented here we were asked to only vary efficacy of Hib, PCV, and Rotavirus vaccines, and causes of death of pneumonia, meningitis, and diarrhoea in our uncertainty analysis. For each scenario we were provided 200 sets of varied vaccine efficacies and causes of death from the VIMC Scientific and technical team, based on the 95% confidence intervals of the vaccine efficacies and causes of death. The distribution of model outputs from the 200 runs were then used to produce the uncertainty bounds, which here were set to capture 95% of the distribution of results.

Appendix D.4.1. PCV-specific assumptions

LiST generates estimates of pneumococcal pneumonia and meningitis cases and deaths averted by the coverage scale-up of PCV. The potential envelope of deaths and cases averted by PCV was derived by applying a proxy for the proportion of pneumonia (product of proportion of S. pneumoniae among chest x-ray positive episodes of pneumonia, and proportion of S. pneumoniae due to PCV13 serotypes by region) [56, 57] and meningitis (product of proportion of S. pneumoniae among severe bacterial meningitis cases, and proportion of S. pneumoniae due to PCV13 serotypes by region) [57, 58] deaths due to S. pneumoniae in the pre-vaccine era to the country-specific estimates of pneumonia and meningitis mortality. Country-specific incidence of severe pneumonia pre-vaccine introduction was derived from an analysis by Rudan and colleagues [56]. The country-specific incidence of bacterial meningitis was calculated using the proportions of bacterial meningitis deaths due to S. pneumoniae and Hib from Davis et al [58], the S. pneumoniae case-fatality rates from O'Brien et al [59], and Hib case-fatality rates from Watt et al [60], divided by the total population 1-59 months of age. The proportion of pneumonia and meningitis cases and deaths averted was calculated by applying the 3-dose coverage of PCV scaled by the 80% efficacy of PCV in preventing PCV13 serotypes of invasive pneumococcal disease [61], and 84% efficacy of PCV in preventing severe bacterial meningitis [58], to the fraction of deaths due to S. pneumoniae. The model includes only the direct effect of complete three-dose vaccination coverage.

Appendix D.4.2. Hib vaccine-specific assumptions

LiST generates estimates of Hib pneumonia and meningitis cases and deaths averted by the coverage scale-up of Hib vaccine. The potential envelope of deaths and cases averted by Hib vaccine was derived by applying proxy estimates of proportion of pneumonia (proportion of Hib among chest x-ray positive episodes of pneumonia) and meningitis (proportion of Hib among severe bacterial meningitis cases) deaths due to Hib in the pre-vaccine era to the country-specific estimates of pneumonia and meningitis mortality [58, 56]. The same country-specific estimates of the incidence of severe pneumonia and meningitis for the PCV impact analysis were used. The proportion of pneumonia and meningitis cases and deaths averted was calculated by applying the three-dose coverage of Hib scaled the 93% efficacy of Hib in preventing invasive pneumococcal disease [44], and the 94% efficacy of Hib in preventing severe bacterial meningitis [58], to the fraction of deaths due to Hib. The model includes only the direct effect of complete three-dose vaccination coverage.

Appendix D.4.3. Rotavirus vaccine-specific assumptions

LiST generates estimates of rotavirus diarrhoea cases and deaths averted by the coverage scale-up of rotavirus vaccine. The potential envelope of deaths averted by rotavirus vaccine was derived by applying region-specific estimates of the proportion of rotavirus among severe diarrhoea cases and deaths in the pre-vaccine era to the country-specific estimates of diarrhoea mortality [38]. Region-specific estimates of the incidence of severe diarrhoea were derived from the same source. The proportion of diarrhoea cases and deaths averted was calculated by applying the complete dose coverage of rotavirus vaccine scaled by the region-specific efficacy of rotavirus vaccine in reducing severe rotavirus gastroenteritis [62] to the fraction of deaths due to rotavirus. The model includes only the direct effect of complete rotavirus vaccination coverage.

Appendix D.5. HepB - Goldstein

The model was developed by Susan Goldstein, Fangjun Zhou, Stephen Hadler, Beth Bell, Eric Mast and Harold Margolis at the US Centers for Disease Control and Prevention (CDC)[63]. It is a static deterministic model that estimates the global burden of hepatitis B and the impact of hepatitis B immunization programs. The model examines the mortality outcomes due to hepatitis B virus (HBV) infection, including deaths of fulminant hepatitis, and deaths of liver cirrhosis and hepatocellular carcinoma as results of chronic hepatitis B. The model assumes infections occur in three age periods with different probabilities of developing symptomatic infections and progressing to chronic hepatitis B, which are: perinatal period, early childhood period (under 5 years), and the period over 5 years of age. The rate of perinatal infection was determined by the prevalence of hepatitis B surface antigen (HBsAg) and hepatitis B e antigen (HBeAg) among pregnant women. The model assumed infants born to HBsAg positive and HBeAg positive mothers had a 90% chance of perinatal infection, while infants born to HBsAg positive and HBeAg negative mothers

had a 10% chance of perinatal infection. The rate of infection in early childhood was determined by the prevalence of antibody to hepatitis B core antigen (anti-HBc) at age 5 after excluding perinatal infections, and the rate of infections between age 5 and 30 was determined by anti-HBc prevalence at 5 and 30 years of age. The prevalence at 30 years of age was assumed to have reached its peak in lifetime. A literature review was conducted on the prevalence of the hepatitis B seromarkers worldwide, and countries were grouped into 15 strata with stratum-specific prevalence based on the reported prevalence in literature and the geographic proximity of the countries.

The model assumes 99% of the infants infected perinatally were asymptomatic during the acute infection phase, and 90% progressed to chronic hepatitis B, regardless of whether they were symptomatic or not. The model assumed 90% of children infected horizontally before the age of 5 had asymptomatic infection and 70% progressed to chronic hepatitis B. After the age of five, the chance of progressing to chronic hepatitis B was much lower: 70% of infections that occurred after the age of 5 were asymptomatic and only 6% progressed to chronic hepatitis B. Of the acute symptomatic infections, the risks of developing fulminant hepatitis B were assumed to be 0.1% for perinatal infections, and 0.6% for infections after the perinatal period. The case-fatality rate of fulminant hepatitis was 70% for all ages. Starting from 20 years of age, a small percentage of chronically infected persons (0.5% annually) seroconverted from HBsAg positive to negative, and were no longer at risk of complications related to chronic hepatitis B.

Liver cirrhosis and hepatocellular carcinoma account for the majority of hepatitis B deaths worldwide. The age-specific liver cirrhosis mortality rates were derived from mortality statistics from the United States and Taiwan (China). The age-specific hepatocellular carcinoma incidence was derived by fitting a polynomial function to data from populations with high HBV prevalence, including Alaska Natives, China, the Gambia and Taiwan (China). Given the low survival rates of hepatocellular carcinoma, the death rate of hepatocellular carcinoma was assumed to be the same as the incidence. The rates were adjusted by the prevalence of HBeAg in each country: populations who were HBeAg positive had 6 times higher the risk of developing hepatocellular carcinoma. The background all-cause mortality rates were from the life table published in the United Nations World Population Prospects. The lives saved by hepatitis B vaccine were calculated as the difference between predicted deaths of hepatitis B in an unvaccinated cohort and a vaccinated cohort born in a certain year in one country. The vaccination coverages, namely the coverage of the timely birth dose (HepB birth dose within 24 hours of birth) and the coverage of the complete series of at least three doses of hepatitis B vaccine (HepB3) were from the WHO-UNICEF Estimates of National Immunization Coverage (WUENIC) of the past years, and the coverage projection was provided by the VIMC secretariat. 95% of infants who received the timely birth dose were assumed to be protected from perinatal infection, and 95% of infants who received the complete series of hepatitis B vaccine (indicated by HepB3 coverage) were assumed to be protected from horizontal infection in their lifetime. The model does

not include herd immunity, or the effect of partial vaccination series.

The key uncertainties of the model resulted from estimates on the prevalence of hepatitis B seromarkers from the pre-vaccination era that were based on limited number of studies in some countries, and the change in mortality of chronic hepatitis B in the long term due to improved access to antiviral treatment. The sensitivity test was run with a spread of six parameters that are normally distributed around the original values, with a range of +/- 5%. Two vaccine efficacy parameters (for HepB3 and birth dose respectively) were originally set to 0.95 and then varied together (with the same value for both) between 0.9 and 1.0, normally distributed. These were not country specific. Four prevalence parameters (HBsAg prevalence, HBeAg prevalence, anti-HBc prevalence at age 5, anti-HBc prevalence at age 30) all had country group-specific central values and were varied by +/- 5% around their central values.

We assumed the same chance of HBV exposure among children who are vaccinated and those who are unvaccinated, and that the probability of receiving HepB3 is independent of the birth dose.

Appendix D.6. HepB - Imperial

This population-level, deterministic, dynamic transmission model contains both acute (Severe Acute and Non-severe Acute) and chronic (Immune Tolerant, Immune Reactive, Asymptomatic Carrier, Chronic Hepatitis B, Compensated Cirrhosis, Decompensated Cirrhosis and Liver Cancer) mutually exclusive disease states [64, 65, 66]. The two acute states as well as the Immune Tolerant and the Immune Reactive states are assumed to contain HBsAg+ HBeAg+ individuals, which are assumed to be 15 times more infectious than the HBsAg+ HBeAg- individuals in the other states [67, 68, 69]. The model also contains separate state variables for susceptible and recovered/vaccinated individuals as well as for individuals on tenofovir antiviral treatment. HBV-related deaths can occur from the Severe Acute, Compensated Cirrhosis, Decompensated Cirrhosis, Liver Cancer and treatment states. The rates of progression through disease stages are informed by literature reviews and assumed to be the same in all settings.

Infection is spread in the population by both vertical and horizontal transmission, the rates of which are informed through fitting. The risk of acute infection becoming chronic is highest in the younger age groups and controlled by an exponential function that ranges in value from 88·5% for vertical infections in infants to less than 5% risk for acute sufferers over 30 years of age [70]. Background mortality and migration are applied equally to individuals in all states. Younger age groups are assumed to undergo seroconversion from being HBsAg+ HBeAg+ to HBsAg+ HBeAg- at a faster rate than older age groups. In contrast, older age groups are at greater risk of developing liver cancer than are younger age groups. Since new HBV cases predominantly occur among the younger age groups (infants and 1 to 5 year olds), population make-up and fertility rates heavily influence the rate of spread of the disease in the population.

The model takes into account the effects of the birth-dose (BD) and the infant vaccines. The BD vaccine is assumed to be 95% effective in protecting infants of mothers that are HBsAg+ HBeAg-, and 83% effective in protecting infants of mothers that are HBsAg+ HBeAg+. All infants are equally likely to be given the BD vaccine within 24 hours of birth. The infant vaccine is assumed to be 95% effective in conferring life-long protection to vaccinated individuals. Individuals are assumed to be either unvaccinated or have been given all infant vaccine doses necessary in their first six months of life to confer the full protection specified in the model. All six-month-olds are equally likely to be given the infant vaccine series.

The model is calibrated to country-level, age-specific HBsAg+ prevalence data and HBeAg+/HBsAg+ prevalence data in pregnant women, obtained from the Polaris Observatory and from other sources,[71, 72] as well as to HBV-related cirrhosis and liver cancer death rates, obtained from the Global Burden of Disease Results Tool website. The calibrated model parameters include the risk of horizontal transmission to susceptible one to five year-olds and the risk of vertical transmission from HBsAg+ HBeAg- mothers to their infants. Calibration is performed by the Approximate Bayesian Computation Sequential Monte Carlo algorithm [73].

Other data sources include demographic data (female and male population sizes of one-year age groups, migration, female fertility rates of five-year age groups, sex ratio of infants, female and male life expectancy of five-year age groups, female and male mortality rates of five-year age groups) from the United Nations World Population Prospects (UN-WPP) 2019 Revision and infant and BD vaccine coverage data from the WHO/UNICEF Estimates of National Immunization Coverage (WUENIC) and Gavi, the Vaccine Alliance.

Uncertainty in model estimates are due to uncertainties in the prevalence data that are used in the model calibration, as well as uncertainties in historical coverage data, vaccine efficacies and the number of individuals on antiviral treatment. The model structure is regularly updated to reflect the latest understanding of the natural history of HBV.

All model code can be found at https://github.com/mrc-ide/icl-hbv.

Appendix D.7. Human Papilloma Virus (HPV) – London School of Hygiene and Tropical Medicine (LSHTM)

The Papillomavirus Rapid Interface for Modelling and Economics (PRIME) is a static, proportional impact model that can estimate the impact of HPV vaccination on cervical cancer cases, deaths, and disability-adjusted life years as well as the cost-effectiveness of vaccination programmes at the global, regional, and national levels [74, 75, 76]. The PRIME model was developed by LSHTM in collaboration with the World Health Organization (WHO), Laval University and Johns Hopkins University. It is designed to estimate the impact and cost-effectiveness of HPV vaccination in low- and middle-income countries (LMICs). In addition to its application in the Vaccine Impact Modelling Consortium

(VIMC), it has been used to support vaccine recommendations by WHO, as well as individual countries. It has been validated against published studies using HPV vaccine economic models set in LMICs [74]. It was also endorsed by the WHO's expert advisory committee, the Immunization and Vaccines Implementation Research Advisory Committee (IVIR-AC) to provide a conservative estimate of the cost effectiveness of vaccinating girls prior to sexual debut. In a model comparison exercise for four countries, it provided similar results to the Harvard static model, with differences mainly driven by alternative interpretation of data on HPV type distribution in cervical cancer [76].

The Excel-based version of the model and documentation are publicly accessible at http://primetool.org/ for use by country programme managers and planners to facilitate country-specific decision-making in LMICs. The R package of the model (prime) has additional functionality such as multiple cohorts and probabilistic sensitivity analysis and is available at https://github.com/lshtm-vimc/prime. It can be used for research, global analyses and to generate the vaccine impact estimates used by VIMC. Data inputs include country and age-specific cervical cancer incidence, prevalence, and mortality among females. The model estimates vaccination impact in terms of reduction in age-dependent incidence of cervical cancer and mortality in direct proportion to vaccine efficacy against HPV 16/18, vaccine coverage, and HPV type distribution. It assumes that vaccinating girls prior to infection with HPV types 16 and 18 fully protects them from developing cervical cancer caused by HPV 16 and 18, in accordance with vaccine trials [77].

The model assumes a one- or two-dose schedule with perfect timeliness at the target ages given in the coverage estimates. Herd effects are not considered meaning that the vaccine impact estimates produced are conservative, although the model can be used in conjunction with transmission dynamic models to project indirect effects. The impact of vaccinating multiple age cohorts is estimated by using the most conservative assumption that 9-14 year old girls who have sexually debuted are not protected, although these assumptions do not change the overall impact estimates significantly [78].

Appendix D.8. Human Papilloma Virus (HPV) – Harvard

Model description matches that published in [6].

Appendix D.9. Japanese Encephalitis – National University of Singapore

This deterministic dynamic model uses a catalytic model in which individuals become infected and are then immune as in [79]. Vaccination is modelled as a removal of susceptibles from the susceptible class. The key parameter is the force of infection (FOI) which is estimated on a small spatial scale using a machine learning algorithm using available data on age of cases and environmental covariates. As humans are dead-end hosts for Japanese encephalitis (JE), infection comes from animal reservoirs via mosquitoes. This model captures the transmission of JE on very fine 5kmx5km scales.

Tree-based machine learning models were used to fit geographic, demographic and bioclimatic data in order to predict the FOI for the pixel level data points. These variables included population numbers, agricultural data, data on pig populations and climatic variables like precipitation. Data from studies in 17 countries was fit and the output of the model was in the form of an FOI estimate for each pixel which was further used to calculate the burden of JE in that pixel. The calculation of cases was done under the catalytic model [79] framework using R programming. In order to generate uncertainty, confidence intervals for each data point were created using quantile regression methods. The symptomatic rate was sampled from uniform distribution (0·002, 0·004) [80]. The proportion of these symptomatic cases that died was calculated using estimates of the Case Fatality Ratio (CFR) that were obtained from modelling data our systematic review of available JE mortality literature [3]. Data from 82 studies which gave 469 age and year stratified data points for the JE CFR records which were included in the analysis [81]. Similar modelling was conducted for the morbidity ratio as well to calculate the DALYs for which 76 records were obtained from 50 articles [81]. The vaccine was assumed to be 100% effective, and protection lifelong. Disease burden was generated from the 'bottom up': i.e. from infection rates applying parameters governing the proportion of infections that are symptomatic and the proportion that die (case fatality ratio).

Appendix D.10. Japanese Encephalitis – University of Notre Dame

We developed a static, stochastic model of Japanese encephalitis virus (JEV) transmission with a constant force of infection (FOI) to estimate the burden of JE and the potential impact of vaccination in JE-endemic countries. JEV is a mosquito-transmitted, zoonotic pathogen that requires an animal host for ongoing transmission, given humans are believed to be a dead-end host [82]. Therefore, JE incidence is limited to geographic regions where there are suitable hosts and vectors to sustain both ongoing transmission in animal hosts and spill-over to humans. To estimate the number of JEV infections, the model first estimates the number of people at risk of infection, and then estimates the transmission intensity in each country. JE burden (including cases, deaths, and DALYs) was then estimated from the number of JEV infections. Key sources of uncertainty in our model are the spatial variation in JEV transmission intensity and the proportion of JEV infections that result in either a severe case or death. Full model details, along with estimates of JE burden and vaccine impact in JE-endemic countries, are available in [83].

To identify the areas suitable for sustained JEV transmission, and the size of the population living in at-risk areas, a spatial analysis of the risk factors associated with JEV was conducted. Potential JEV-endemic areas were identified using large-scale spatiotemporal datasets related to suitable climate conditions for the vector species, suitable habitat conditions for the vector, and the presence of potential zoonotic hosts. Transmission was assumed to occur only in areas occupied by the primary vector, Culex tritaeniorhynchus [84], or where the annual minimum temperature exceeded 20C and annual precipitation

exceeded 150 cm. Suitable habitat conditions included areas with rice cultivation or nearby wetlands [85]. Within these suitable areas, people were considered at risk of infection if the density of domestic pigs or fowl exceeded 2 per km (with uncertainty represented by varying the animal threshold from 0-10 per km) [86]. Risk maps were validated using seroprevalence and surveillance data obtained from a literature review [83].

Next, the FOI in each country was estimated from age-specific incidence data using a catalytic model. FOI represents the per-capita rate at which susceptible individuals are infected. Age-specific incidence data was obtained from a literature search, restricted to studies conducted in areas with no history of vaccination (or prior to documented vaccination) to simplify the estimation process. For several countries where no age-specific incidence data was available, FOI estimates were drawn from the posterior estimate of a neighbouring country. FOI estimates for each study were estimated using a maximum likelihood approach, using the observed numbers of JE cases per age class in each year. Study-specific FOI values were estimated using a Bayesian framework via a Markov chain Monte Carlo (MCMC) approach implemented in the software package STAN.

The annual number of JEV infections for a given study area were then calculated from the FOI estimate and the size of the at-risk population. In the absence of vaccination, the number of infections in age class was calculated by multiplying the age-specific probability of infection by the number of at-risk individuals in the age class. Vaccination reduced the number of at-risk individuals in each targeted age class based on provided coverage estimates. We assumed that all vaccinated individuals received a full vaccine regimen, and that routine and campaign-based vaccinations were independent. The number of JE cases and deaths were then estimated from the number of JEV infections based on the proportion of infections that are symptomatic or fatal. The probability of asymptomatic infection and the case fatality ratio for symptomatic infections were obtained from a published systematic review [81]. The annual burden of JE at the national-level was calculated using disability-adjusted life years (DALYs) with disability weights taken from the Global Burden of Disease 2016 report [87].

Appendix D.11. Measles – London School of Hygiene and Tropical Medicine (LSHTM)

DynaMICE (DYNAmic Measles Immunisation Calculation Engine) is a measles transmission and vaccination model developed by LSHTM with input from Harvard University and the University of Montreal [88, 89]. It has been previously used to inform policies on measles-containing vaccines by WHO; this work has been reviewed by the WHO Immunization and Vaccines Implementation Research Advisory Committee as well as WHO Strategic Advisory Group of Experts on Immunization measles and rubella working group. The model structure and assumptions have previously been published in detail [88, 89].

DynaMICE is an age-structured compartmental transmission dynamic model with compartments for maternal immune, susceptible, infected, recovered, and vaccinated subpopulations. In the model, a measles case is defined as a susceptible who acquires new infection,

and the number of deaths is estimated by age-specific and country-specific case-fatality risks [90]. The population is stratified by age with weekly age classes up to age 3 years, and annual age classes thereafter up to 100 years. The force of infection is calculated by combining information from synthetic social contact matrices based on country survey data and the POLYMOD study [91], demographic characteristics from each country, the transmission potential of measles from a systematic review of the basic reproduction number in different settings [92], the length of the infectious period (14 days), and time-varying proportions of infectious population. Vaccination is incorporated as a pulse function and can be delivered to targeted age or groups through either routine immunisation or supplementary immunisation activities (SIAs). The uncertainty around SIA dose distribution to children who miss routine vaccination is captured using analyses of Demographic and Health Surveys and Multiple Indicator Cluster Surveys [93, 94]. Vaccine efficacy is dependent on age and the number of doses received [95, 96]. The immunity derived from vaccination or natural infection is assumed to be lifelong.

The ability of SIAs to reach children who miss routine vaccination is determined using analyses of Demographic and Health Survey (DHS) data [93]. Vaccine efficacy is dependent on age and the number of doses received [95]. The model has been previously described in detail [88].

Appendix D.12. Measles – Pennsylvania State University (PSU)

The PSU measles model is a dynamic, age-structured, discrete time-step, annual SIR model. Unlike conventional SIR models, which describe dynamics at the scale of an infectious generation [97] or finer [98], it models the aggregate number of cases over one-year time steps. While this is coarse relative to the time scale of measles transmission, it matches the annual reporting of measles cases available for all countries, since approximately 1980, for all countries through the WHO Joint Reporting Form (JRF). To account for the fine-scale dynamics that are being summed over a full year, the model describes the number of infections ($I_{i,t}$) in country i and year t, and age class a as an increasing function of the fraction, $p_{i,t}$, of the population susceptible in age class a at the start of year t, $S_{i,t}$:

$$E[I_{a,i,t}] = p_{i,t} * S_{a,i,t},$$

where $E[\cdot]$ indicates the expectation and $p_{i,t}$ is a country and year specific annualized attack rate modeled as:

$$p_{i,t} = invlogit(-\beta_{0,i} + \beta_{1,i} * \frac{\sum_a S_{i,t}}{N_{i,t}} + e_t),$$

where invlogit() indicates the inverse logit function, $N_{i,t}$ is the total population size in country i and year t over all age classes, and e_t is a Gaussian random variable with mean 0 and variance σ^2. The parameters $\beta_{0,i}$, $\beta_{1,i}$, and σ^2 are fit to each country independently using a state-space model fitted to observed annual cases reported through the JRF from 1980-2016 as described by Eilertson et al (2019) and Dixon et al. (2021) [99, 100]. Inferred cases

are not constrained by external mortality estimates. Historical population and vaccination coverage values are provided by WHO as described by Simons et al (2012) [101].

The number of susceptible individuals in each single-year age class a (a=2,..., 100) is equal to the number not infected in the previous year, nor immunized through supplemental immunization activities (SIAs). The number susceptible is further deprecated by the crude death rate. The efficacy of doses administered through SIAs is assumed to be 99%; SIA doses are assumed to be independent of prior routine immunization. The number of susceptible individuals in age class a=1 is assumed to be 50% of the annual live birth cohort; this assumes that all children have protective maternal immunity until 6 months of age. Age class a=2 and a=m is assumed to receive a first and second dose (respectively) of routine measles vaccination before the start of the time step. We assume that the second routine dose is delivered only to those who have received the first routine dose. Efficacy is assumed to be 85% and 93% for the first dose in countries delivering at 9m and 12m of age, respectively, and assumed to be 99% for the second dose.

Deaths are calculated by applying an age and country specific case fatality ratio (CFR) to each country. CFRs for cases below 59 months of age for all countries were taken from Portnoy et al., 2019; CFR for cases above 59 months of age are assumed to be 50% lower than those applying to under 5s [90].

Forward simulations of this model assume random variation in the annual attack rate according to the parameter σ^2. Future vaccination coverage values, for routine and SIAs, are assumed known and future birth and death rates are assumed known.

Appendix D.13. MenA - Cambridge

The University of Cambridge MenA model is a compartmental transmission dynamic model of Neisseria meningitidis group A (NmA) carriage and disease to investigate the impact of immunisation with a group A meningococcal conjugate vaccine, known as MenAfriVac, as published by Karachaliou et al. [102] The model is age-structured (1-year age groups up to age 100) with continuous ageing between groups. Model parameters were based on the available literature and African data wherever possible, with the model calibrated on an ad-hoc basis as described below.

The population is divided into four states, which represent their status with respect to the meningitis infection. Individuals may be susceptible, carriers, ill or recovered, and in each of these states be vaccinated or unvaccinated, with vaccinated individuals having lower risks of infection (carriage acquisition) and disease (rate of invasion). We assume that both carriers and ill individuals are infectious and can transmit the bacteria to susceptible individuals. The model captures the key features of meningococcal epidemiology, including seasonality, which is implemented by forcing the transmission rate, the extent of which varies stochastically every year.

Since only a small proportion of infected individuals develop the invasive disease, disease-induced deaths are not included in the model. From each compartment, there is

a natural death rate from all causes. Carriage prevalence and disease incidence vary with age, and the model parameterised these distributions using a dataset from Niger [103]; the case:carrier ratio consequently varies with age. The duration of 'natural immunity' is an important driver of disease dynamics in the absence of vaccination but good data on this parameter is lacking; instead, prior estimates are used [104].

The model assumes that mass vaccination campaigns occur as discrete events whereas routine immunisation takes place continuously. We allowed the duration of protection to vary uniformly between 5 years and 20 years for the 0-4-year-olds and 10-20 years for over 5-year-olds. For the 200 runs, we selected pairs of values for these two parameters so that duration of protection for the older age group is not shorter than the duration of protection for 0-4-year-olds [105, 106]. Vaccine efficacy against carriage and disease is 90%.

Disease surveillance is not comprehensive across the meningitis belt, so the disease burden is uncertain in several countries. Therefore, the model classifies the countries into three categories, based on the incidence levels using historical data. This classification defines the transmission dynamic parameters. The model generates estimates of case incidence, to which a 10% case-fatality ratio is applied to estimate mortality [107]. To estimate DALYs it is assumed that 7·2% of survivors have major disabling sequelae with a disability weight of 0·26 [35].

Countries were stratified into high and medium risk, and different infection risks applied based on this stratification. As there was insufficient information to define infection risk on a country-by-country basis, the approach/stratification was agreed upon with experts in the WHO meningitis team. For countries only partly within the meningitis belt, only the (subnational) area at risk was included.

To produce estimates on the impact of vaccination, 200 simulation runs were generated by stochastically varying the baseline transmission rate to reflect between-year climactic or another external variability. Although each individual simulation reflects the reality of irregular and periodic epidemics, as visually compared to time series from Chad and Burkina Faso and analysis of inter-epidemic periods, the resulting averaged estimates give a stable expected burden of disease over time. Uncertainty in other model parameters is currently not quantified.

Appendix D.14. MenA - KPW

Appendix D.15. PCV – London School of Hygiene and Tropical Medicine (LSHTM) and National University of Singapore (NUS)

Our model re-evaluates of the epidemiological impact of PCV on children under-5 by combining and adapting two existing models from Chen et al. [108] and Clark et al [32, 109]. As the UNIVAC model did not account for indirect effects (herd protection and serotype replacements), we estimated the impact of PCV introduction from Chen et al [108], which in turn used projections from a quasi-dynamic model by Flasche et al [108, 110]. The quasi-dynamic model is an ecological model [110] of PCV impact on invasive

pneumococcal disease (IPD), which simplifies long-term disease projection using a single equation known as the predicted incidence risk ratio (IRR).

We used estimates from the UNIVAC model (i.e., disaggregated age-weeks groups, country-specific PCV coverage and vaccine timeliness) in Clark et al [109] and updated the vaccine impact using the predicted IRR from the ecological model in Chen et al [108, 110]. The updated model also included recent country-specific data wherever possible (i.e., disease incidence rates, disease mortality rates, vaccine coverages and vaccine efficacy). Additionally, we extended the model by Chen et al [108] to incorporate three additional characteristics sing recent emerging research from low- and middle-income countries: (i) time taken to near elimination of VT, (ii) vaccine coverage required to reach full vaccine impact, (iii) differentiated PCV impact on non-invasive pneumococcal disease (nIPD).

In the updated model, we included four categories of disease outcomes attributable to S. pneumoniae, namely, pneumonia, meningitis, non-pneumonia non-meningitis (NPNM), and acute otitis media (AOM). Under the no vaccination scenario, we estimated disease burden (i.e., cases, deaths and DALYs) for each birth cohort in age weeks (from birth to under-5) by multiplying country-specific disease rates and the size of each birth cohort. Accounting for vaccine introduction and timeliness, estimates in the no vaccination scenario were then multiplied by the predicted IRRs from the ecological model adjusted for the three characteristics mentioned above to reflect the burden of diseases after PCV introduction. The predicted IRRs from the ecological model [110] were specific to six United Nations (UN) regions (Africa, Asia, Europe, Latin American and the Caribbean, North America and Oceania), where countries in each region will have the same IRRs.

Appendix D.16. Rotavirus – Emory University

The Emory model is a dynamic, deterministic, age-structured compartmental transmission model simulates rotavirus transmission and estimates disease incidence/burden in a given country. The model is based on a Susceptible–Infected–Recovered (SIR) structure, with elaborations in order to capture the complexities of rotavirus immunity and transmission. In particular, individuals can be infected up to four times. We model the following age groups: 0-1 months, 2-3 months, 4-11 months, 1-year age bands from 1 to 4 years old, and 5 years and older. We use realistic, age-specific population sizes, aging and death rates.

In the model, infants are born with maternal immunity [111]. After maternal immunity wanes, infants become susceptible to a primary rotavirus infection. We assume that protection is conferred by previous infections against subsequent infections, such that the proportion of individuals that remain susceptible to re-infection decreases with each subsequent infection [112, 113]. Primary, secondary, tertiary, and quaternary infections are assumed to have the same duration of infectiousness, however non-primary infections have lower per-contact infectiousness relative to primary infections [114]. Immunity is assumed to be a mix of ‘take type’ where a portion of individuals develop long-term immunity, while others remain fully susceptible to subsequent infections. We assume primary, secondary,

tertiary, and quaternary infections had different probabilities for developing rotavirus gastroenteritis [113]. We assume only severe rotavirus gastroenteritis cases are reported to surveillance and can result in death. This model incorporates the introduction of vaccines in a specified year, delivered to 2- and 4-month olds. We assume that vaccine doses are independent. We incorporate an immunogenicity parameter that determines whether individuals will respond to the vaccine [115]. If individuals respond to vaccination, we assume that the vaccine acts like a natural infection; the probability of becoming infected, given vaccination and natural history of infections, goes down with each subsequent vaccine dose and natural infection. Values for natural history parameters were set to values identified in birth cohort and challenge studies.

In lieu of fitting this model to estimate country-specific effective contact rates, we used a linear regression model to estimate the mean age of severe rotavirus infection, and subsequently calculated the basic reproduction number. Variables considered for inclusion were: under 5 mortality rates [116], gross domestic product (GDP) per capita [116], total GDP [116], region, sub-region, birthrate [33], life expectancy [33], and percent of the population living in a rural setting [116]. The linear regression model was fit using a training (80%) and validation (20%) data set, with the optimal model for each country being selected to optimize the correlation accuracy and the mean absolute percent error (MAPE). The basic reproduction number was calculated by dividing the life expectancy for each country [116] by the fitted average age of severe infection.

To account for uncertainty, we generated 200 parameter sets by uniformly sampling from the published range of vaccine immunogenicity and the 95% confidence interval of the regression models estimated mean age of infection for each country. The remaining parameters were fixed. We then simulated the model for each set of fixed and sampled parameters. We calculated the central burden/impact estimate as the median of the 200 probabilistic runs. To calculate the number of deaths, we estimated the number of previous rotavirus infections (with first infections being most severe and subsequent infections being less likely to cause severe disease) and then multiplied this quantity by the estimated rotavirus case fatality ratio for each country and age group, based on data from the Global Burden of Disease Study [117].

Appendix D.17. Rubella – University of Georgia

We developed a discrete-time stochastic age-structured compartmental rubella transmission model, building from previous work describing rubella dynamics [118, 119]. The key feature of the model is a matrix that at every time-step defines transitions from every combination of epidemiological stage (maternally immune 'M', susceptible 'S', infected 'I', recovered 'R', and vaccinated 'V', taken to indicate the effectively vaccinated) and age group (1 month age groups up to 20 years old, then 1 year age groups up to 100 years old) to every other possible combination of epidemiological stage and age group. The discrete time-step was set to about two weeks (i.e. 24 time steps in a year), the approximate

generation time of rubella.

Demographic parameters (population size, crude birth rates, and age-specific death rates) and vaccination coverage were time and country-specific, and were supplied by VIMC. We assumed dependence between routine vaccine doses. We adjusted campaign coverage based on the assumptions that a portion of the population may always remain inaccessible to campaigns. We assumed the age and time-specific proportion inaccessible corresponds to WUENIC DTP routine vaccination rates [120] if it does not exceed routine coverage. Duration of maternal immunity [121] and vaccine efficacy [122] were assumed from published literature and are constant across time and country. The annual introduction of infected individuals was scaled with the median time-specific population size of each country set to trigger an outbreak if the size of the susceptible population was large enough but small enough to not effect probability of elimination.

Country-specific transmission to individuals in age group a from individuals in age group j for each time-step t is defined by $\beta_{a,j,t} = \overline{\beta_{a,j,}}(1+\alpha cos(2\pi t))$, where $\overline{\beta_{a,j}}$ is mean transmission from individuals in age group j to age group a, and α is a parameter controlling the magnitude of seasonal fluctuations (assumed 0.15 [119] and constant over time and country). Mean transmission from individuals in age class j to age class a, $\overline{\beta_{a,j}}$, was estimated by rescaling population-adjusted age-contact rates (time constant and country-specific [123]) to reflect the assumed basic reproductive number (R_0) of rubella. R_0 distributions were country-specific and estimated by fitting a dampened exponential model [124] with likelihood-based MCMC to published rubella immunoglobulin G (IgG) seroprevalence data.

Rubella burden is generated from a 'bottom up' approach in which we calculate CRS cases, and deaths, from modeled output. Country- age- and time-specific CRS cases were estimated by multiplying the country, age and time-specific number of susceptible individuals, the country and time-specific sex ratio of the population, the country- and age-specific fertility rate, the country- age- and time-specific probability of becoming infected over 16 week period, and finally the probability of CRS following rubella infection during the first 16 weeks of pregnancy (estimated ~0.4 [125, 126, 127, 128, 129, 130, 131]). Fetal deaths were estimated directly from rubella infections among women in the first 16 weeks of pregnancy as 20.7 per 100 [129, 132, 133, 134, 135] and infant deaths were estimated from the number of CRS cases as 8.9 per 100 [133, 132, 136, 137, 138].

We simulated 200 stochastic runs for each country from the year 1980 to 2100. Model uncertainty includes process uncertainty for all epidemiological and demographic transition and uncertainty from the R_0, CRS rate, and CRS death rate distributions. Model input parameters (e.g., R_0) were fit to empirical data, however the mechanistic transmission model itself is not directly fit to data.

Appendix D.18. Rubella – UKHSA

This is an age and sex-structured, deterministic, compartmental model of the transmission dynamics of rubella [139, 140, 141]. The population is stratified into those with maternal immunity (lasting 6 months), susceptible, pre-infectious (infected but not yet infectious), infectious and immune, using annual age bands and a "Realistic Age Structure" [142]. Country-specific birth and age-specific death rates were fixed at 2010 levels and calculated from UN population survival data for 2010-15 [143] respectively. The supplement to reference [139] provides the model's differential equations.

The force of infection (rate at which susceptibles are infected) changes over time and is calculated using the number of infectious individuals and the effective contact rate (rate at which infectious and susceptible individuals come into effective contact). Contact is described using the following matrix of "Who Acquires Infection From Whom":

$$\begin{pmatrix} \beta_1 & 0.7\beta_2 \\ 0.7\beta_2 & \beta_2 \end{pmatrix}$$

The effective contact rate differs between <13 and ≥ 13 year olds, with its relative size based on contact survey data [91]. β_1 and β_2 are calculated from the average force of infection in <13 and ≥ 13 year olds, estimated from age-stratified rubella seroprevalence data, which had been collected before rubella containing vaccine (RCV) was introduced [140]. Seroprevalence data were available for 39 countries (see [141] and manuscript in preparation). For countries lacking seroprevalence data, we used data from countries in the same WHO region [140, 141]. Confidence intervals (CI) on the force of infection were calculated using 1000 bootstrap-derived-seroprevalence datasets [140, 141]. The vaccine doses were assumed to be correlated, with 100% of those vaccinated previously being vaccinated I SIAs, where possible and 50% of those who have received RCV1 receiving RCV2, where possible.

Country-specific numbers of congenital rubella syndrome (CRS) cases in year y during 2001-2080 were calculated by summing the number of CRS cases born each day to women aged 15-49 years. As assumed elsewhere [139, 140, 141, 144], infection during the first 16 weeks of pregnancy carries a 65% risk of the newborn having CRS. The number of CRS deaths in year y was calculated by multiplying the number of CRS cases born in year y by the assumed case fatality rate (30%). The latter was assumed to have a plausible range of 10-50%, consistent with the number of DALYs for cases in year y was calculated by multiplying the number of CRS cases in year y by the corresponding DALY [145], which was based on the country-specific World Bank Income group for 2017 [146]. Both the DALYs and the assigned World Bank income group remained fixed over time. As rubella infections are mild, rubella-specific deaths are not included and people with rubella infection are assumed to die at the general all-cause, age and sex-specific mortality rate.

Confidence intervals on the outputs for each setting were calculated as the 95% range of the outputs obtained by running the model using 200 combinations of 5 randomly-sampled

parameters. The parameters were the pre-vaccination force of infection which was used to calculate the contact parameters (see above), the risk of a child being born with CRS if his/her mother had been infected during pregnancy, the CRS-related case-fatality rate, the vaccine coverage and the vaccine efficacy. The pre-vaccination force of infection was sampled from 1000 bootstrap-derived force of infection estimates, obtained by fitting catalytic models to bootstrap-derived seroprevalence data for that setting, or, if that setting lacked seroprevalence data, from bootstrap-derived force of infection estimates from countries in the same WHO region as the country of interest [140, 141]. The remaining parameters were randomly sampled from distributions reflecting their plausible range, as implied by published studies, wherever possible [139]. For example, the CRS-related mortality was sampled from the uniform distribution in the range 10-50%, consistent with estimates from 3 studies in Vietnam, Greece and Panama, in which the 95% confidence intervals were 20-51%, 12-50% and 15-40% respectively [138, 147, 136]. The risk of a child being born with CRS to a mother infected in the first 16 weeks of pregnancy was sampled from the Gamma distribution with shape and scale parameters 37 and 56 respectively. This assumption leads to a median and 95% range of 65% and 47-88% respectively for this risk, consistent with estimates from several studies [133, 127, 126]) which, as found in a recent review [148] were likely to have been more reliable than those in other studies. The sampling was conducted assuming that the parameters were independent.

Appendix D.19. Typhoid fever - Yale

Our model is an age-structured, deterministic compartmental model that follows an SIRS-like framework. The model includes six infection states: S1, fully susceptible to typhoid infection; I1, infected with the potential for symptomatic illness and diagnosis with clinical disease; R, recovered from infection and temporarily immune to reinfection; S2, susceptible to subclinical infection; I2, subclinically infected with Salmonella Typhi; and C, chronically infected with S. Typhi. We assume individuals are born into the S compartment of the lowest age-stratum. In the absence of immunizations, the susceptible population is infected and enters the I1 compartment at a rate λ, i.e. the force of infection. Following primary infection, an age-specific fraction of infectious individuals become lifelong chronic carriers (C), and the remaining (majority) fraction recover and gain temporary immunity to reinfection (R). Individuals lose immunity and enter compartment S2, where they are susceptible to subclinical reinfection (I2). Individuals who experience re-infection are all assumed to recover and again enter the R compartment, where they are temporarily immune to reinfection. We assume no waning of partial immunity to symptomatic reinfection (i.e. from S2 to S1), since previous estimates of this rate were not significantly different from zero. The model also differentiates between fully susceptible individuals who are vaccinated and protected (V1), and those who were vaccinated after a previous infection (V2); individuals return to the respective susceptible state (S1 or S2) following the waning of vaccine-induced immunity. Estimated parameters include the basic reproductive

number (R0), factors for the relative risk of infection for children under age 2 (m1) and children ages 2 to 5 (m2), and a reporting fraction.

The dynamic model includes 6 age groups: 0-9m, 9m-2y, 2y-4y, 5y-14y, 15y-24y, 25y+. The model outputs total incidence and age-specific incidence (per 100,000 person-years) over time for those age groups. In order to make the model calibration process computationally feasible for a large number of countries, we simulated typhoid fever incidence across a broad range of R0 values for three standard demographic profiles, then sampled from the values of R0 that yielded an average age of infection consistent with estimates for each country (as described in [149]). We then estimated a reporting fraction to scale the model-predicted incidence of infection to the range of typhoid fever incidence rates predicted by three different published burden models for the year 2016 [150, 26, 27].

Given the long time-horizon of the analysis (2000 to 2100), we modeled a scenario in which we assumed the typhoid transmission rate would decline over time in the absence of vaccination due to improvements in water, sanitation, and hygiene. We assumed an exponential decrease in the transmission rate parameter (b) in year y at a rate w:

$$\beta(y) = \beta_0 \exp -w(y-2016), \tag{D.1}$$

where β_0 is the baseline transmission parameter, which is derived from the R0 value that matches estimates of the average age of typhoid fever cases in each country in 2016. Since the rate of decline in typhoid transmission is uncertain and likely varies from country to country, we sampled the rate of decline, w, from a uniform distribution between 0 (i.e. no decline in the typhoid transmission rate) and 0.025 (i.e. a 25% decline in typhoid transmission over 10 years).

To generate age-specific incidence for the 1-year age categories, we used the model to predict the age-specific typhoid incidence rates for each of the 6 age groups, and then interpolated the 1-year age-specific incidence rates using flexible splines. We did this for each year from 2000 to 2100 for each vaccination scenario and multiplied the 1-year age-specific rates by the cohort size for their respective year and age group to generate age-specific case numbers for each year.

To estimate the number of deaths, we multiplied the number of cases in each year and age group by the case fatality risk (CFR). Because we had more reliable data on the CFR for typhoid inpatients, we used an estimate of the proportion of patients hospitalized [25] multiplied by the CFR among hospitalized patients [151] multiplied by the case count to generate an estimate of the number of deaths among hospitalized patients. We used country-specific data for the CFR where available and a meta-analysis estimate of the CFR for the remaining countries [149]. To account for the large uncertainty in the CFR among non-hospitalized patients, we assumed that a proportion of deaths, p, between 0% and 75% (uniform distribution) occurs outside the hospital. Thus, to estimate the total number of deaths, we divided the estimated number of deaths among hospitalized patients by (1 – p),

i.e. the sampled proportion of deaths occurring in hospital. We assumed that the CFR does not vary by age or over time.

To estimate years of life lost due to death (YLLs), we multiplied the number of deaths in each age group in each year by a corresponding matrix of the life expectancy at birth for individuals of each age in each year. For individuals born before 1950, we used the life expectancy at birth values for 1950. To estimate years of life lived with disability (YLDs), we based our assumptions about the disability weights for inpatients, outpatients, and those not seeking care for typhoid fever on the disability weights for acute infectious diseases (mild, moderate, severe) from the 2010 GBD study [152]). We used the 2010 GBD estimates because we felt these more accurately reflected the perspective of individuals in LMICs where typhoid is most prevalent. In order to acknowledge the uncertainty around how to assign disability weights to the different healthcare use groups (i.e. inpatients, outpatients, and those not seeking care for typhoid fever), we sampled evenly from two different options, one which yielded an average disability weight for an episode of typhoid fever of 0.04, and another which yielded an average weight of 0.15 [149].

Appendix D.20. Yellow Fever – Imperial College London

The Imperial College yellow fever (YF) transmission model is a static force of infection (FOI) epidemiological model. The first iteration was originally published by Garske et al.; however, this was extensively updated by Gaythorpe et al. to provide the 2019 model estimates [153, 154] and the same model is used for the 2021 estimates. The model is fitted at the first administrative level or province level for all countries considered at risk or endemic for YF. In each administrative unit, the force of infection is assumed to be constant across the observation period and across age groups. This is analogous to assuming that all yellow fever transmission occurs as a result of spillover events from the sylvatic reservoir. As a result, this model variant includes no herd immunity effects.

We use published values of the proportion of infections which are severe and of the CFR to calculate the burden of disease. The estimates of the proportions of infections that are severe are taken from Johansson, et al. [155], as 12% [5%, 26%]. The case fatality ratio is taken from Servadio et al. as 39% (95% CI: [30%, 48%]) [156]. This is in contrast to the published estimates in the previous VIMC-wide publication which used the higher CFR estimate of Johansson et al. [6]. As such, the estimates of YF vaccine impact measured in deaths have dropped for the Imperial modelling group, but the impact in terms of cases has remained unchanged. However, these estimates remain uncertain since the disease is notoriously misdiagnosed and under-reported. As such, whilst the updated data and model averaging framework have improved the uncertainty ranges, these are still broad.

Appendix D.21. Yellow Fever – University of Notre Dame

Model description matches that published in [6].

Appendix E. GATHER guidelines

Table E1: GATHER guidelines from [157] with either the location in the text where the information is included or a direct response.

Item number	Checklist item	Response/ location in text
Objectives and funding		
1	Define the indicator(s), populations (including age, sex, and geographic entities), and time period(s) for which estimates were made.	Indicators: deaths, cases and DALYs; populations: 112 countries, all ages, all genders; time periods: 2020 - 2030 for vaccination activities but modelling 2020 - 2100.
2	List the funding sources for the work.	Funding stated in brief in the abstract and in full in the declaration of interests section (section **??**)
Data inputs		
For all data inputs from multiple sources that are synthesised as part of the study:		
3	Describe how the data were identified and how the data were accessed.	Data detailed in methods and epidemiological data is detailed in the model descriptions- section Appendix D. In summary: Demography: UNWPP; Coverage: detailed in methods and in section Appendix A.2.
4	Specify the inclusion and exclusion criteria. Identify all ad-hoc exclusions.	NA

5	Provide information about all included data sources and their main characteristics. For each data source used, report reference information or contact name/institution, population represented, data collection method, year(s) of data collection, sex and age range, diagnostic criteria or measurement method, and sample size, as relevant.	Data detailed in methods and epidemiological data is detailed in the model descriptions- section Appendix D.
6	Identify and describe any categories of input data that have potentially important biases (eg, based on characteristics listed in item 5).	Data detailed in methods and epidemiological data is detailed in the model descriptions- section Appendix D. Limitations and uncertainty detailed in discussion.
For data inputs that contribute to the analysis but were not synthesised as part of the study:		
7	Describe and give sources for any other data inputs.	Data detailed in methods and epidemiological data is detailed in the model descriptions- section Appendix D.
For all data inputs:		
8	Provide all data inputs in a file format from which data can be efficiently extracted (eg, a spreadsheet rather than a PDF), including all relevant meta-data listed in item 5. For any data inputs that cannot be shared because of ethical or legal reasons, such as third-party ownership, provide a contact name or the name of the institution that retains the right to the data.	Data access is detailed in the data sharing section.
Data analysis		
9	Provide a conceptual overview of the data analysis method. A diagram may be helpful.	Methods are detailed in the main text with model specific methods detailed in section Appendix D.

10	Provide a detailed description of all steps of the analysis, including mathematical formulae. This description should cover, as relevant, data cleaning, data pre-processing, data adjustments and weighting of data sources, and mathematical or statistical model(s).	Methods are detailed in the main text with model specific methods detailed in section Appendix D.
11	Describe how candidate models were evaluated and how the final model(s) were selected.	Methods are detailed in the main text with model specific methods detailed in section Appendix D.
12	Provide the results of an evaluation of model performance, if done, as well as the results of any relevant sensitivity analysis.	Methods are detailed in the main text with model specific methods detailed in section Appendix D.
13	Describe methods of calculating uncertainty of the estimates. State which sources of uncertainty were, and were not, accounted for in the uncertainty analysis.	Methods are detailed in the main text with model specific methods detailed in section Appendix D.
14	State how analytical or statistical source code used to generate estimates can be accessed.	This will be available in the data sharing section and individual model publications.
Results and discussion		
15	Provide published estimates in a file format from which data can be efficiently extracted.	This will be linked through the data sharing section
16	Report a quantitative measure of the uncertainty of the estimates (eg, uncertainty intervals).	All estimates in tables include the uncertainty intervals - all figures shown uncertainty bounds where feasible.
17	Interpret results in light of existing evidence. If updating a previous set of estimates, describe the reasons for changes in estimates.	Estimates of the impact of coverage disruption have not been published previously, comparisons to estimates of [158] have been made where relevant in the results section

18	Discuss limitations of the estimates. Include a discussion of any modelling assumptions or data limitations that affect interpretation of the estimates.	Included in the discussion.

References

[1] Beth Evans and Thibaut Jombart. Worldwide routine immunisation coverage regressed during the first year of the COVID-19 pandemic. *Vaccine*, 40(26):3531–3535, jun 2022.

[2] Shurendar Selva Kumar, Anna-Maria Hartner, Arunah Chandran, Katy AM Gaythorpe, and Xiang Li. Evaluating effective measles vaccine coverage in the malaysian population accounting for between-dose correlation and vaccine efficacy. 2023.

[3] World Health Organisation. Member States Information Session- The global immunization "Big Catch-Up" effort, 2023.

[4] Emily D Carter, Linnea Zimmerman, Jiage Qian, Tim Roberton, Assefa Seme, and Solomon Shiferaw. Impact of the Early Stages of the COVID-19 Pandemic on Coverage of Reproductive, Maternal, and Newborn Health Interventions in Ethiopia: A Natural Experiment. *Frontiers in public health*, 10:778413, jun 2022.

[5] Immunization Agenda 2030 Secretariat. Framework for Action: Strengthening Immunization to Advance the Global Health Agenda (Version 04). https://www.immunizationagenda2030.org/images/documents/IA2030$_{A}nnex_{F}rameworkForActionv0$[illegible] $May10,2023$.

[6] Jaspreet Toor, Susy Echeverria-Londono, Xiang Li, Kaja Abbas, Emily D Carter, Hannah E Clapham, Andrew Clark, Margaret J de Villiers, Kirsten Eilertson, Matthew Ferrari, et al. Lives saved with vaccination for 10 pathogens across 112 countries in a pre-COVID-19 world. *Elife*, 10:e67635, 2021.

[7] Elizabeth C Lee, Andrew S Azman, Joshua Kaminsky, Sean M Moore, Heather S McKay, and Justin Lessler. The projected impact of geographic targeting of oral cholera vaccination in sub-Saharan Africa: a modeling study. *PLoS medicine*, 16(12):e1003003, 2019.

[8] J. Lessler, S. M. Moore, F. J. Luquero, H. S. McKay, R. Grais, M. Henkens, et al. Mapping the burden of cholera in sub-Saharan Africa and implications for control: an analysis of data across geographical scales. *The Lancet*, 391:1908–1915, 2018.

[9] World Health Organisation. Weekly Epidemiological record: Annual cholera reports, 2020.

[10] Andrew J Tatem. WorldPop, open data for spatial demography. *Scientific data*, 4(1):1–4, 2017.

[11] GADM. Database od global administrative areas, 2020.

[12] Mohammad Ali, Michael Emch, Lorenz Von Seidlein, Mohammad Yunus, David A Sack, Malla Rao, Jan Holmgren, and John D Clemens. Herd immunity conferred by killed oral cholera vaccines in Bangladesh: a reanalysis. *The Lancet*, 366(9479):44–49, 2005.

[13] Mohammad Ali, Dipika Sur, Young Ae You, Suman Kanungo, Binod Sah, Byomkesh Manna, Mahesh Puri, Thomas F Wierzba, Allan Donner, G Balakrish Nair, et al. Herd protection by a bivalent killed whole-cell oral cholera vaccine in the slums of Kolkata, India. *Clinical Infectious Diseases*, 56(8):1123–1131, 2013.

[14] Q. Bi, E. Ferreras, L. Pezzoli, D. Legros, L. C. Ivers, K. Date, et al. Protection against cholera from killed whole-cell oral cholera vaccines: a systematic review and meta-analysis. *The Lancet Infectious Diseases*, 17:1080–1088, 2017.

[15] Qulu Zheng, Francisco J Luquero, Iza Ciglenecki, Joseph F Wamala, Abdinasir Abubakar, Placide Welo, Mukemil Hussen, Mesfin Wossen, Sebastian Yennan, Alama Keita, et al. Cholera outbreaks in sub-Saharan Africa during 2010-2019: A Descriptive Analysis. *International Journal of Infectious Diseases*, 2022.

[16] Kim J-h, V. Mogasale, C. Burgess, and Wierzba TF. Impact of oral cholera vaccines in cholera-endemic countries: A mathematical modeling study. *Vaccine*, 34:2113–2120, 2016.

[17] M. Wolfe, M. Kaur, T. Yates, M. Woodin, and Lantagne D. A Systematic Review and. and Meta-Analysis of the Association between Water, Sanitation, and Hygiene Exposures and Cholera in Case-Control Studies. *Am J Trop Med Hyg*, 99:534–545, 2018.

[18] S. Brockett, M. K. Wolfe, A. Hamot, G. D. Appiah, E. D. Mintz, and Lantagne D. Associations among Water. Sanitation, and Hygiene, and Food Exposures and Typhoid Fever in Case–Control Studies: A Systematic Review and Meta-Analysis. *The American Journal of Tropical Medicine and Hygiene*, 103:1020–1031, 2020.

[19] A. A. King, E. L. Ionides, C. M. Breto, S. P. Ellner, M. J. Ferrari, B. E. Kendall, et al. pomp: Statistical Inference for Partially Observed Markov Processes (R package. *version*, 3:5.

[20] P. Bratley and Fox BL. Algorithm 659. Implementing Sobol's quasirandom sequence generator. *ACM Trans Math Softw*, 14:88–100, 1988.

[21] D. G. Altman and Bland JM. How to obtain the P value from a confidence interval. *BMJ*, 343, 2011.

[22] Global Health Observatory data repository. *https://apps.who.int/gho/data/node.main.174?lang=en*, 2022.

[23] J. L. Deen, L. von Seidlein, D. Sur, M. Agtini, Lucas Mes, A. L. Lopez, et al. The high burden of cholera in children: comparison of incidence from endemic areas in Asia and Africa. *PLoS Negl Trop Dis*, 2, 2008.

[24] Jane J Kim, Karen M Kuntz, Natasha K Stout, Salaheddin Mahmud, Luisa L Villa, Eduardo L Franco, and Sue J Goldie. Multiparameter calibration of a natural history model of cervical cancer. *American Journal of Epidemiology*, 166(2):137–150, jul 2007.

[25] V. Mogasale, B. Maskery, R. L. Ochiai, J. S. Lee, V. V. Mogasale, E. Ramani, et al. Burden of typhoid fever in low-income and middle-income countries: a systematic, literature-based update with risk-factor adjustment. *The Lancet Global Health*, 2, 2014.

[26] M. Antillon, J. L. Warren, F. W. Crawford, et al. The burden of typhoid fever in low- and middle-income countries: A meta-regression approach. *PLoS Neglected Tropical Diseases*, 11:2, 2017.

[27] J. D. Stanaway, R. C. Reiner, B. F. Blacker, et al. The global burden of typhoid and paratyphoid fevers: a systematic analysis for the Global Burden of Disease Study 2017. *Lancet Infect Dis*, 19:369, 2019.

[28] C. S. Marchello, M. Birkhold, and Crump JA. Complications and. and mortality of typhoid fever: A global systematic review and meta-analysis. *J Infect*, 81:902–910, 2020.

[29] F. Qadri, F. Khanam, X. Liu, K. Theiss-Nyland, P. K. Biswas, A. I. Bhuiyan, et al. Protection by vaccination of children against typhoid fever with a Vi-tetanus toxoid conjugate vaccine in urban Bangladesh: a cluster-randomised trial. *The Lancet*, 398:675–684, 2021.

[30] P. D. Patel, P. Patel, Y. Liang, J. E. Meiring, T. Misiri, F. Mwakiseghile, et al. Safety and Efficacy of a Typhoid Conjugate Vaccine in Malawian Children. *New England Journal of Medicine*, 385:1104–1115, 2021.

[31] M. Shakya, R. Colin-Jones, K. Theiss-Nyland, M. Voysey, D. Pant, N. Smith, et al. Phase 3 Efficacy Analysis of a Typhoid Conjugate Vaccine Trial in Nepal. *N Engl J Med*, 381:2209–2218, 2019.

[32] Andrew Clark, Barbara Jauregui, Ulla Griffiths, Cara B Janusz, Brenda Bolaños-Sierra, Rana Hajjeh, Jon K Andrus, and Colin Sanderson. TRIVAC decision-support model for evaluating the cost-effectiveness of Haemophilus influenzae type b, pneumococcal and rotavirus vaccination. *Vaccine*, 31:C19–C29, 2013.

[33] United Nations. World Population Prospects - Population Division, 2019.

[34] Brian Wahl, Katherine L O'Brien, Adena Greenbaum, Anwesha Majumder, Li Liu, Yue Chu, Ivana Lukšić, Harish Nair, David A McAllister, Harry Campbell, et al. Burden of Streptococcus pneumoniae and Haemophilus influenzae type b disease in children in the era of conjugate vaccines: global, regional, and national estimates for 2000–15. *The Lancet Global Health*, 6(7):e744–e757, 2018.

[35] Karen Edmond, Andrew Clark, Viola S Korczak, Colin Sanderson, Ulla K Griffiths, and Igor Rudan. Global and regional risk of disabling sequelae from bacterial meningitis: a systematic review and meta-analysis. *The Lancet Infectious Diseases*, 10(5):317–328, may 2010.

[36] Andrew Clark, Robert Black, Jacqueline Tate, Anna Roose, Karen Kotloff, Diana Lam, William Blackwelder, Umesh Parashar, Claudio Lanata, Gagandeep Kang, Christopher Troeger, James Platts-Mills, Ali Mokdad, Global Rotavirus Surveillance Network, Colin Sanderson, Laura Lamberti, Myron Levine, Mathuram Santosham, and Duncan Steele. Estimating global, regional and national rotavirus deaths in children aged <5 years: Current approaches, new analyses and proposed improvements. *Plos One*, 12(9):e0183392, sep 2017.

[37] Joke Bilcke, Pierre Van Damme, Marc Van Ranst, Niel Hens, Marc Aerts, and Philippe Beutels. Estimating the incidence of symptomatic rotavirus infections: a systematic review and meta-analysis. *PloS one*, 4(6):e6060, 2009.

[38] Christa L Fischer Walker, Igor Rudan, Li Liu, Harish Nair, Evropi Theodoratou, Zulfiqar A Bhutta, Katherine L O'Brien, Harry Campbell, and Robert E Black. Global burden of childhood pneumonia and diarrhoea. *The Lancet*, 381(9875):1405–1416, apr 2013.

[39] Mateusz Hasso-Agopsowicz, Chandresh Nanji Ladva, Benjamin Lopman, Colin Sanderson, Adam L Cohen, Jacqueline E Tate, Ximena Riveros, Ana Maria Henao-Restrepo, Andrew Clark, Global Rotavirus Surveillance Network, and Rotavirus Age Study Collaborators. Global review of the age distribution of rotavirus disease in children aged <5 years before the introduction of rotavirus vaccination. *Clinical Infectious Diseases*, 69(6):1071–1078, aug 2019.

[40] Li Liu, Shefali Oza, Dan Hogan, Yue Chu, Jamie Perin, Jun Zhu, Joy E Lawn, Simon Cousens, Colin Mathers, and Robert E Black. Global, regional, and national causes of under-5 mortality in 2000-15: an updated systematic analysis with implications for the Sustainable Development Goals. *The Lancet*, 388(10063):3027–3035, dec 2016.

[41] USAID. The demographic and health surveys (DHS) program. 2020.

[42] UNICEF. Multiple Indicator Cluster Surveys (MICS). 2020.

[43] Andrew Clark and Colin Sanderson. Timing of children's vaccinations in 45 low-income and middle-income countries: an analysis of survey data. *The Lancet*, 373(9674):1543–1549, may 2009.

[44] U K Griffiths, A Clark, B Gessner, A Miners, C Sanderson, E R Sedyaningsih, and K E Mulholland. Dose-specific efficacy of Haemophilus influenzae type b conjugate vaccines: a systematic review and meta-analysis of controlled clinical trials. *Epidemiology and Infection*, 140(8):1343–1355, aug 2012.

[45] Andrew Clark, Kevin van Zandvoort, Stefan Flasche, Colin Sanderson, Julie Bines, Jacqueline Tate, Umesh Parashar, and Mark Jit. Efficacy of live oral rotavirus vaccines by duration of follow-up: a meta-regression of randomised controlled trials. *The Lancet Infectious Diseases*, 19(7):717–727, jun 2019.

[46] World Health Organization. Global Health Observatory data repository: causes of child death., 2018.

[47] M J Fox, R Marterell, N Van den Broek, and N Walker. Technical inputs, enhancements and applications of the Lives Saved Tool (LiST). *BMC Public Health*, 11(Suppl 3), 2011.

[48] Adrienne Clermont and Neff Walker. Nutrition interventions in the lives saved tool (list). *The Journal of Nutrition*, 147(11):2132S–2140S, sep 2017.

[49] N Walker and I Friberg. The Lives Saved Tool in 2017: Updates, Applications, and Future Directions. *BMC Public Health*, 17(Suppl 4), 2017.

[50] Agbessi Amouzou, Stephanie A Richard, Ingrid K Friberg, Jennifer Bryce, Abdullah H Baqui, Shams El Arifeen, and Neff Walker. How well does LiST capture mortality by wealth quintile? A comparison of measured versus modelled mortality rates among children under-five in Bangladesh. *International Journal of Epidemiology*, 39 Suppl 1:i186–92, apr 2010.

[51] Ingrid K Friberg, Zulfiqar A Bhutta, Gary L Darmstadt, Abhay Bang, Simon Cousens, Abdullah H Baqui, Vishwajeet Kumar, Neff Walker, and Joy E Lawn. Comparing modelled predictions of neonatal mortality impacts using LiST with observed results of community-based intervention trials in South Asia. *International Journal of Epidemiology*, 39 Suppl 1:i11–20, apr 2010.

[52] Elizabeth Hazel, Kate Gilroy, Ingrid Friberg, Robert E Black, Jennifer Bryce, and Gareth Jones. Comparing modelled to measured mortality reductions: applying the Lives Saved Tool to evaluation data from the Accelerated Child Survival Programme in West Africa. *International Journal of Epidemiology*, 39 Suppl 1:i32–9, apr 2010.

[53] David A Larsen, Ingrid K Friberg, and Thomas P Eisele. Comparison of Lives Saved Tool model child mortality estimates against measured data from vector control studies in sub-Saharan Africa. *BMC Public Health*, 11 Suppl 3:S34, apr 2011.

[54] Jim Ricca, Debra Prosnitz, Henry Perry, Anbrasi Edward, Melanie Morrow, Pieter Ernst, and Leo Ryan. Comparing estimates of child mortality reduction modelled in LiST with pregnancy history survey data for a community-based NGO project in Mozambique. *BMC Public Health*, 11 Suppl 3:S35, apr 2011.

[55] Cesar G Victora, Aluisio J D Barros, Tanya Malpica-Llanos, and Neff Walker. How within-country inequalities and co-coverage may affect LiST estimates of lives saved by scaling up interventions. *BMC Public Health*, 13 Suppl 3:S24, sep 2013.

[56] Igor Rudan, Katherine L O'Brien, Harish Nair, Li Liu, Evropi Theodoratou, Shamim Qazi, Ivana Lukšić, Christa L Fischer Walker, Robert E Black, Harry Campbell, and Child Health Epidemiology Reference Group (CHERG). Epidemiology and etiology of childhood pneumonia in 2010: estimates of incidence, severe morbidity, mortality, underlying risk factors and causative pathogens for 192 countries. *Journal of global health*, 3(1):010401, jun 2013.

[57] Hope L Johnson, Maria Deloria-Knoll, Orin S Levine, Sonia K Stoszek, Laura Freimanis Hance, Richard Reithinger, Larry R Muenz, and Katherine L O'Brien. Systematic evaluation of serotypes causing invasive pneumococcal disease among children under five: the pneumococcal global serotype project. *PLoS Medicine*, 7(10), oct 2010.

[58] Stephanie Davis, Daniel Feikin, and Hope L Johnson. The effect of Haemophilus influenzae type B and pneumococcal conjugate vaccines on childhood meningitis mortality: a systematic review. *BMC Public Health*, 13 Suppl 3:S21, sep 2013.

[59] Katherine L O'Brien, Lara J Wolfson, James P Watt, Emily Henkle, Maria Deloria-Knoll, Natalie McCall, Ellen Lee, Kim Mulholland, Orin S Levine, Thomas Cherian,

Hib, and Pneumococcal Global Burden of Disease Study Team. Burden of disease caused by Streptococcus pneumoniae in children younger than 5 years: global estimates. *The Lancet*, 374(9693):893–902, sep 2009.

[60] James P Watt, Lara J Wolfson, Katherine L O'Brien, Emily Henkle, Maria Deloria-Knoll, Natalie McCall, Ellen Lee, Orin S Levine, Rana Hajjeh, Kim Mulholland, Thomas Cherian, Hib, and Pneumococcal Global Burden of Disease Study Team. Burden of disease caused by Haemophilus influenzae type b in children younger than 5 years: global estimates. *The Lancet*, 374(9693):903–911, sep 2009.

[61] M G Lucero, V E Dulalia, R N Parreno, D M Lim-Quianzon, H Nohynek, H Makela, and G Williams. Pneumococcal conjugate vaccines for preventing vaccine-type invasive pneumococcal disease and pneumonia with consolidation on x-ray in children under two years of age. *Cochrane Database of Systematic Reviews*, (4):CD004977, oct 2004.

[62] Laura M Lamberti, Sania Ashraf, Christa L Fischer Walker, and Robert E Black. A systematic review of the effect of rotavirus vaccination on diarrhea outcomes among children younger than 5 years. *The Pediatric Infectious Disease Journal*, 35(9):992–998, 2016.

[63] Susan T Goldstein, Fangjun Zhou, Stephen C Hadler, Beth P Bell, Eric E Mast, and Harold S Margolis. A mathematical model to estimate global hepatitis B disease burden and vaccination impact. *International Journal of Epidemiology*, 34(6):1329–1339, Dec 2005.

[64] Margaret J de Villiers, Ivane Gamkrelidze, Timothy B Hallett, Shevanthi Nayagam, Homie Razavi, and Devin Razavi-Shearer. Modelling hepatitis B virus infection and impact of timely birth dose vaccine: A comparison of two simulation models. *PloS one*, 15(8):e0237525, 2020.

[65] Margaret J de Villiers, Shevanthi Nayagam, and Timothy B Hallett. The impact of the timely birth dose vaccine on the global elimination of hepatitis B. *Nature communications*, 12(1):1–10, 2021.

[66] Shevanthi Nayagam, Mark Thursz, Elisa Sicuri, Lesong Conteh, Stefan Wiktor, Daniel Low-Beer, and Timothy B Hallett. Requirements for global elimination of hepatitis B: a modelling study. *The Lancet Infectious Diseases*, 16(12):1399–1408, 2016.

[67] ME Mendy, M Fortuin, AJ Hall, AD Jack, and HC Whittle. Hepatitis B virus DNA in relation to duration of hepatitis B surface antigen carriage. *British journal of biomedical science*, 56(1):34, 1999.

[68] Maimuna E Mendy, Samuel J McConkey, Sarah Crozier, Steve Kaye, David Jeffries, Andrew J Hall, Hilton C Whittle, et al. Changes in viral load and HBsAg and HBeAg status with age in HBV chronic carriers in The Gambia. *Virology Journal*, 5(1):1–8, 2008.

[69] Elisabeth Keane, AL Funk, and Yusuke Shimakawa. Systematic review with meta-analysis: the risk of mother-to-child transmission of hepatitis B virus infection in sub-Saharan Africa. *Alimentary pharmacology & therapeutics*, 44(10):1005–1017, 2016.

[70] WJ Edmunds, GF Medley, DJ Nokes, AJ Hall, and HC Whittle. The influence of age on the development of the hepatitis B carrier state. *Proceedings of the Royal Society of London. Series B: Biological Sciences*, 253(1337):197–201, 1993.

[71] JJ Ott, GA Stevens, J Groeger, and ST Wiersma. Global epidemiology of hepatitis B virus infection: new estimates of age-specific HBsAg seroprevalence and endemicity. *Vaccine*, 30(12):2212–2219, 2012.

[72] Jördis J Ott, Gretchen A Stevens, and Steven T Wiersma. The risk of perinatal hepatitis B virus transmission: hepatitis B e antigen (HBeAg) prevalence estimates for all world regions. *BMC infectious diseases*, 12(1):1–9, 2012.

[73] Tina Toni, David Welch, Natalja Strelkowa, Andreas Ipsen, and Michael PH Stumpf. Approximate Bayesian computation scheme for parameter inference and model selection in dynamical systems. *Journal of the Royal Society Interface*, 6(31):187–202, 2009.

[74] Mark Jit, Marc Brisson, Allison Portnoy, and Raymond Hutubessy. Cost-effectiveness of female human papillomavirus vaccination in 179 countries: a PRIME modelling study. *The Lancet. Global health*, 2(7):e406–14, jul 2014.

[75] Kaja M Abbas, Kevin van Zandvoort, Marc Brisson, and Mark Jit. Effects of updated demography, disability weights, and cervical cancer burden on estimates of human papillomavirus vaccination impact at the global, regional, and national levels: a PRIME modelling study. *The Lancet. Global health*, 8(4):e536–e544, feb 2020.

[76] Allison Portnoy, Kaja Abbas, Steven Sweet, Jane J Kim, and Mark Jit. Projections of human papillomavirus (HPV) vaccination impact in Ethiopia, India, Nigeria and Pakistan: a comparative modelling study. *BMJ Global Health*, 6(11):e006940, 2021.

[77] John T Schiller, Xavier Castellsagué, and Suzanne M Garland. A review of clinical trials of human papillomavirus prophylactic vaccines. *Vaccine*, 30 Suppl 5:F123–38, nov 2012.

[78] Mark Jit and Marc Brisson. Potential lives saved in 73 countries by adopting multi-cohort vaccination of 9-14-year-old girls against human papillomavirus. *International Journal of Cancer*, 143(2):317–323, jul 2018.

[79] Tran Minh Quan, Tran Thi Nhu Thao, Nguyen Manh Duy, Tran Minh Nhat, and Hannah Clapham. Estimates of the global burden of Japanese encephalitis and the impact of vaccination from 2000-2015. *eLife*, 9, may 2020.

[80] SAGE Working Group on Japanese encephalitis vaccines. Background Paper on Japanese Encephalitis Vaccines. *World Health Organization*, 2014.

[81] Yuwei Cheng, Nhat Tran Minh, Quan Tran Minh, Shreya Khandelwal, and Hannah E Clapham. Estimates of Japanese Encephalitis mortality and morbidity: A systematic review and modeling analysis. *PLOS Neglected Tropical Diseases*, 16(5):e0010361, 2022.

[82] Andrew F van den Hurk, Scott A Ritchie, and John S Mackenzie. Ecology and geographical expansion of Japanese encephalitis virus. *Annual Review of Entomology*, 54:17–35, 2009.

[83] Sean M Moore. The current burden of Japanese encephalitis and the estimated impacts of vaccination: Combining estimates of the spatial distribution and transmission intensity of a zoonotic pathogen. *PLoS neglected tropical diseases*, 15(10):e0009385, 2021.

[84] Joshua Longbottom, Annie J Browne, David M Pigott, Marianne E Sinka, Nick Golding, Simon I Hay, Catherine L Moyes, and Freya M Shearer. Mapping the spatial distribution of the Japanese encephalitis vector, Culex tritaeniorhynchus Giles, 1901 (Diptera: Culicidae) within areas of Japanese encephalitis risk. *Parasites & vectors*, 10(1):148, mar 2017.

[85] Murali Krishna Gumma, Andrew Nelson, Prasad S Thenkabail, and Amrendra N Singh. Mapping rice areas of South Asia using MODIS multitemporal data. *Journal of applied remote sensing*, 5(1):053547, 2011.

[86] Timothy P Robinson, G R William Wint, Giulia Conchedda, Thomas P Van Boeckel, Valentina Ercoli, Elisa Palamara, Giuseppina Cinardi, Laura D'Aietti, Simon I Hay, and Marius Gilbert. Mapping the global distribution of livestock. *Plos One*, 9(5):e96084, may 2014.

[87] Simon I Hay, Amanuel Alemu Abajobir, Kalkidan Hassen Abate, Cristiana Abbafati, Kaja M Abbas, Foad Abd-Allah, Rizwan Suliankatchi Abdulkader, Abdishakur M Abdulle, Teshome Abuka Abebo, Semaw Ferede Abera, et al. Global, regional, and national disability-adjusted life-years (DALYs) for 333 diseases and injuries and healthy life expectancy (HALE) for 195 countries and territories, 1990–2016: a systematic analysis for the Global Burden of Disease Study 2016. *The Lancet*, 390(10100):1260–1344, 2017.

[88] Stéphane Verguet, Mira Johri, Shaun K Morris, Cindy L Gauvreau, Prabhat Jha, and Mark Jit. Controlling measles using supplemental immunization activities: a mathematical model to inform optimal policy. *Vaccine*, 33(10):1291–1296, mar 2015.

[89] Han Fu, Kaja Abbas, Petra Klepac, Kevin van Zandvoort, Hira Tanvir, Allison Portnoy, and Mark Jit. Effect of evidence updates on key determinants of measles vaccination impact: a DynaMICE modelling study in ten high-burden countries. *BMC Medicine*, 19(1):281, nov 2021.

[90] Allison Portnoy, Mark Jit, Matthew Ferrari, Matthew Hanson, Logan Brenzel, and Stéphane Verguet. Estimates of case-fatality ratios of measles in low-income and middle-income countries: a systematic review and modelling analysis. *The Lancet. Global health*, 7(4):e472–e481, feb 2019.

[91] Joël Mossong, Niel Hens, Mark Jit, Philippe Beutels, Kari Auranen, Rafael Mikolajczyk, Marco Massari, Stefania Salmaso, Gianpaolo Scalia Tomba, Jacco Wallinga, Janneke Heijne, Malgorzata Sadkowska-Todys, Magdalena Rosinska, and W John Edmunds. Social contacts and mixing patterns relevant to the spread of infectious diseases. *PLoS Medicine*, 5(3):e74, mar 2008.

[92] Fiona M Guerra, Shelly Bolotin, Gillian Lim, Jane Heffernan, Shelley L Deeks, Ye Li, and Natasha S Crowcroft. The basic reproduction number (R0) of measles: a systematic review. *The Lancet Infectious Diseases*, 17(12):e420–e428, 2017.

[93] Allison Portnoy, Mark Jit, Stéphane Helleringer, and Stéphane Verguet. Impact of measles supplementary immunization activities on reaching children missed by routine programs. *Vaccine*, 36(1):170–178, jan 2018.

[94] Bianca O Cata-Preta, Thiago M Santos, Tewodaj Mengistu, Daniel R Hogan, Aluisio JD Barros, and Cesar G Victora. Zero-dose children and the immunisation cascade: understanding immunisation pathways in low and middle-income countries. *Vaccine*, 39(32):4564–4570, 2021.

[95] Stephanie L Hughes, Shelly Bolotin, Sumaiya Khan, Ye Li, Caitlin Johnson, Lindsay Friedman, Andrea C Tricco, Susan J M Hahné, Jane M Heffernan, Alya Dabbagh,

David N Durrheim, Walter A Orenstein, William J Moss, Mark Jit, and Natasha S Crowcroft. The effect of time since measles vaccination and age at first dose on measles vaccine effectiveness - A systematic review. *Vaccine*, 38(3):460–469, jan 2020.

[96] Christopher R Sudfeld, Ann Marie Navar, and Neal A Halsey. Effectiveness of measles vaccination and vitamin A treatment. *International journal of epidemiology*, 39(suppl_1):i48–i55, 2010.

[97] B F Finkenstadt and B T Grenfell. Timeseries modelling of childhood diseases. *Applied statistics*, 49(Part 2):187–205, 2000.

[98] R M Anderson and R M May. *Infectious Disease of Humans: Dynamics and Control.* Oxford Univerity Press, 1991.

[99] Kirsten E Eilertson, John Fricks, and Matthew J Ferrari. Estimation and prediction for a mechanistic model of measles transmission using particle filtering and maximum likelihood estimation. *Statistics in Medicine*, 38(21):4146–4158, sep 2019.

[100] Meredith G Dixon, Matt Ferrari, Sebastien Antoni, Xi Li, Allison Portnoy, Brian Lambert, Sarah Hauryski, Cynthia Hatcher, Yoann Nedelec, Minal Patel, et al. Progress toward regional measles elimination—worldwide, 2000–2020. *Morbidity and Mortality Weekly Report*, 70(45):1563, 2021.

[101] Emily Simons, Matthew Ferrari, John Fricks, Kathleen Wannemuehler, Abhijeet Anand, Anthony Burton, and Peter Strebel. Assessment of the 2010 global measles mortality reduction goal: results from a model of surveillance data. *The Lancet*, 379(9832):2173–2178, jun 2012.

[102] Andromachi Karachaliou, Andrew J K Conlan, Marie-Pierre Preziosi, and Caroline L Trotter. Modeling Long-term Vaccination Strategies With MenAfriVac in the African Meningitis Belt. *Clinical Infectious Diseases*, 61 Suppl 5:S594–600, nov 2015.

[103] G Campagne, A Schuchat, S Djibo, A Ousséini, L Cissé, and J P Chippaux. Epidemiology of bacterial meningitis in Niamey, Niger, 1981-96. *Bulletin of the World Health Organization*, 77(6):499–508, 1999.

[104] T J Irving, K B Blyuss, C Colijn, and C L Trotter. Modelling meningococcal meningitis in the African meningitis belt. *Epidemiology and Infection*, 140(5):897–905, may 2012.

[105] Seydou Yaro, Berthe-Marie Njanpop Lafourcade, Soumeya Ouangraoua, Aline Ouoba, Hervé Kpoda, Helen Findlow, Haoua Tall, Joy Seanehia, Catherine Martin, Jean-Bosco Ouedraogo, Bradford Gessner, Nicolas Meda, Ray Borrow, Caroline Trotter, and Judith E Mueller. Antibody Persistence at the Population Level 5 Years After Mass Vaccination With Meningococcal Serogroup A Conjugate Vaccine (PsA-TT) in Burkina Faso: Need for a Booster Campaign? *Clinical Infectious Diseases*, 68(3):435–443, jan 2019.

[106] Michael White, Olubukola Idoko, Samba Sow, Aldiouma Diallo, Beate Kampmann, Ray Borrow, and Caroline Trotter. Antibody kinetics following vaccination with MenAfriVac: an analysis of serological data from randomised trials. *The Lancet Infectious Diseases*, 19(3):327–336, feb 2019.

[107] Clément Lingani, Cassi Bergeron-Caron, James M Stuart, Katya Fernandez, Mamoudou H Djingarey, Olivier Ronveaux, Johannes C Schnitzler, and William A Perea. Meningococcal Meningitis Surveillance in the African Meningitis Belt, 2004-2013. *Clinical Infectious Diseases*, 61 Suppl 5:S410–5, nov 2015.

[108] Cynthia Chen, Francisco Cervero Liceras, Stefan Flasche, Sucitro Sidharta, Joanne Yoong, Neisha Sundaram, and Mark Jit. Effect and cost-effectiveness of pneumococcal conjugate vaccination: a global modelling analysis. *The Lancet Global Health*, 7(1):e58–e67, 2019.

[109] About UNIVAC — Provac Toolkit, 2022.

[110] Stefan Flasche, Olivier Le Polain de Waroux, Katherine L O'Brien, and W John Edmunds. The serotype distribution among healthy carriers before vaccination is essential for predicting the impact of pneumococcal conjugate vaccine on invasive disease. *PLoS computational biology*, 11(4):e1004173, 2015.

[111] A C Linhares, Y B Gabbay, R B Freitas, E S da Rosa, J D Mascarenhas, and E C Loureiro. Longitudinal study of rotavirus infections among children from Belém, Brazil. *Epidemiology and Infection*, 102(1):129–145, feb 1989.

[112] Beryl P Gladstone, Sasirekha Ramani, Indrani Mukhopadhya, Jayaprakash Muliyil, Rajiv Sarkar, Andrea M Rehman, Shabbar Jaffar, Miren Iturriza Gomara, James J Gray, David W G Brown, Ulrich Desselberger, Sue E Crawford, Jacob John, Sudhir Babji, Mary K Estes, and Gagandeep Kang. Protective effect of natural rotavirus infection in an Indian birth cohort. *The New England Journal of Medicine*, 365(4):337–346, jul 2011.

[113] F R Velázquez, D O Matson, J J Calva, L Guerrero, A L Morrow, S Carter-Campbell, R I Glass, M K Estes, L K Pickering, and G M Ruiz-Palacios. Rotavirus infection

in infants as protection against subsequent infections. *The New England Journal of Medicine*, 335(14):1022–1028, oct 1996.

[114] Virginia E Pitzer, Katherine E Atkins, Birgitte Freiesleben de Blasio, Thierry Van Effelterre, Christina J Atchison, John P Harris, Eunha Shim, Alison P Galvani, W John Edmunds, Cécile Viboud, Manish M Patel, Bryan T Grenfell, Umesh D Parashar, and Ben A Lopman. Direct and indirect effects of rotavirus vaccination: comparing predictions from transmission dynamic models. *Plos One*, 7(8):e42320, aug 2012.

[115] Manish Patel, Andi L Shane, Umesh D Parashar, Baoming Jiang, Jon R Gentsch, and Roger I Glass. Oral rotavirus vaccines: how well will they work where they are needed most? *The Journal of Infectious Diseases*, 200 Suppl 1:S39–48, nov 2009.

[116] World Bank. Countries and economies., 2021.

[117] Christopher Troeger, Ibrahim A Khalil, Puja C Rao, Shujin Cao, Brigette F Blacker, Tahmeed Ahmed, George Armah, Julie E Bines, Thomas G Brewer, Danny V Colombara, Gagandeep Kang, Beth D Kirkpatrick, Carl D Kirkwood, Jason M Mwenda, Umesh D Parashar, William A Petri, Mark S Riddle, A Duncan Steele, Robert L Thompson, Judd L Walson, John W Sanders, Ali H Mokdad, Christopher J L Murray, Simon I Hay, and Robert C Reiner. Rotavirus vaccination and the global burden of rotavirus diarrhea among children younger than 5 years. *JAMA pediatrics*, 172(10):958–965, oct 2018.

[118] C J E Metcalf, J Lessler, P Klepac, F Cutts, and B T Grenfell. Impact of birth rate, seasonality and transmission rate on minimum levels of coverage needed for rubella vaccination. *Epidemiology and Infection*, 140(12):2290–2301, dec 2012.

[119] C J E Metcalf, J Lessler, P Klepac, A Morice, B T Grenfell, and O N Bj. Structured models of infectious disease: inference with discrete data. *Theoretical Population Biology*, 82(4):275–282, dec 2012.

[120] year=2020 World Health Organization UNICEF. *Estimates of National Immunization Coverage (WUENIC), estimates for 1980 to 2019.*

[121] C Nicoara, K Zäch, D Trachsel, D Germann, and L Matter. Decay of passively acquired maternal antibodies against measles, mumps, and rubella viruses. *Clinical and Diagnostic Laboratory Immunology*, 6(6):868–871, nov 1999.

[122] N Boulianne, G De Serres, S Ratnam, B J Ward, J R Joly, and B Duval. Measles, mumps, and rubella antibodies in children 5-6 years after immunization: effect of vaccine type and age at vaccination. *Vaccine*, 13(16):1611–1616, nov 1995.

[123] Kiesha Prem, Alex R Cook, and Mark Jit. Projecting social contact matrices in 152 countries using contact surveys and demographic data. *PLoS Computational Biology*, 13(9):e1005697, sep 2017.

[124] C P Farrington. Modelling forces of infection for measles, mumps and rubella. *Statistics in Medicine*, 9(8):953–967, aug 1990.

[125] Joelma Queiroz Andrade, Victor Bunduki, Suely Pires Curti, Cristina Adelaide Figueiredo, Maria Isabel de Oliveira, and Marcelo Zugaib. Rubella in pregnancy: intrauterine transmission and perinatal outcome during a Brazilian epidemic. *Journal of Clinical Virology*, 35(3):285–291, mar 2006.

[126] Susan Hahné, Jeannette Macey, Rob van Binnendijk, Robert Kohl, Sharon Dolman, Ytje van der Veen, Graham Tipples, Helma Ruijs, Tony Mazzulli, Aura Timen, Anton van Loon, and Hester de Melker. Rubella outbreak in the Netherlands, 2004-2005: high burden of congenital infection and spread to Canada. *The Pediatric Infectious Disease Journal*, 28(9):795–800, sep 2009.

[127] L Grillner, M Forsgren, B Barr, M Böttiger, L Danielsson, and C De Verdier. Outcome of rubella during pregnancy with special reference to the 17th-24th weeks of gestation. *Scandinavian Journal of Infectious Diseases*, 15(4):321–325, 1983.

[128] E Miller. Rubella in the united kingdom. *Epidemiology and Infection*, 107(1):31–42, aug 1991.

[129] Mariam M Mirambo, Said Aboud, Mtebe Majigo, Uwe Groβ, and Stephen E Mshana. Adverse pregnancy outcomes among pregnant women with acute Rubella infections in Mwanza city, Tanzania. *International Journal of Infectious Diseases*, 78:72–77, jan 2019.

[130] I Zgórniak-Nowosielska, B Zawilińska, and S Szostek. Rubella infection during pregnancy in the 1985-86 epidemic: follow-up after seven years. *European Journal of Epidemiology*, 12(3):303–308, jun 1996.

[131] M Vejtorp and B Mansa. Rubella IgM antibodies in sera from infants born after maternal rubella later than the 12th week of pregnancy. *Scandinavian Journal of Infectious Diseases*, 12(1):1–5, 1980.

[132] L Z Cooper and S Krugman. Clinical manifestations of postnatal and congenital rubella. *Archives of Ophthalmology*, 77(4):434–439, apr 1967.

[133] E Miller, J E Cradock-Watson, and T M Pollock. Consequences of confirmed maternal rubella at successive stages of pregnancy. *The Lancet*, 2(8302):781–784, oct 1982.

[134] M Siegel, H T Fuerst, and N S Peress. Comparative fetal mortality in maternal virus diseases. A prospective study on rubella, measles, mumps, chicken pox and hepatitis. *The New England Journal of Medicine*, 274(14):768–771, apr 1966.

[135] Morris Siegel, Harold T. Fuerst, and Nancy S. Peress. Fetal mortality in maternal rubella. *American Journal of Obstetrics and Gynecology*, 96(2):247–253, sep 1966.

[136] C Saad de Owens and R Tristan de Espino. Rubella in Panama: still a problem. *The Pediatric Infectious Disease Journal*, 8(2):110–115, feb 1989.

[137] T Panagiotopoulos, I Antoniadou, and E Valassi-Adam. Increase in congenital rubella occurrence after immunisation in Greece: retrospective survey and systematic review. *BMJ (Clinical Research Ed.)*, 319(7223):1462–1467, dec 1999.

[138] Michiko Toizumi, Hideki Motomura, Hien Minh Vo, Kensuke Takahashi, Enga Pham, Hien Anh Thi Nguyen, Tho Huu Le, Masahiro Hashizume, Koya Ariyoshi, Duc Anh Dang, Hiroyuki Moriuchi, and Lay-Myint Yoshida. Mortality associated with pulmonary hypertension in congenital rubella syndrome. *Pediatrics*, 134(2):e519–26, aug 2014.

[139] Emilia Vynnycky, Lay Myint Yoshida, Dang Thi Thanh Huyen, Nguyen Dac Trung, Kohei Toda, Nguyen Van Cuong, Duong Thi Hong, Koya Ariyoshi, Masami Miyakawa, Hiroyuki Moriuchi, Le Huu Tho, Hien Anh Nguyen, Dang Duc Anh, Mark Jit, and Nguyen Tran Hien. Modeling the impact of rubella vaccination in Vietnam. *Human vaccines & immunotherapeutics*, 12(1):150–158, 2016.

[140] Emilia Vynnycky, Elisabeth J Adams, Felicity T Cutts, Susan E Reef, Ann Marie Navar, Emily Simons, Lay-Myint Yoshida, David W J Brown, Charlotte Jackson, Peter M Strebel, and Alya J Dabbagh. Using Seroprevalence and Immunisation Coverage Data to Estimate the Global Burden of Congenital Rubella Syndrome, 1996-2010: A Systematic Review. *Plos One*, 11(3):e0149160, mar 2016.

[141] Emilia Vynnycky, Timoleon Papadopoulos, and Konstantinos Angelis. The impact of Measles-Rubella vaccination on the morbidity and mortality from Congenital Rubella Syndrome in 92 countries. *Human vaccines & immunotherapeutics*, 15(2):309–316, 2019.

[142] D Schenzle. An age-structured model of pre- and post-vaccination measles transmission. *IMA journal of mathematics applied in medicine and biology*, 1(2):169–191, 1984.

[143] UNWPP. The 2017 Revision of the United Nations World Population Prospects, 2017.

[144] E Vynnycky, N J Gay, and F T Cutts. The predicted impact of private sector MMR vaccination on the burden of Congenital Rubella Syndrome. *Vaccine*, 21(21-22):2708–2719, jun 2003.

[145] Emily A. Simons, Susan E. Reef, Louis Z. Cooper, Laura Zimmerman, and Kimberly M. Thompson. Systematic Review of the Manifestations of Congenital Rubella Syndrome in Infants and Characterization of Disability-Adjusted Life Years (DALYs). *Risk Analysis*, 36(7):1332–1356, jul 2016.

[146] World Bank. World Development Indicators., 2017.

[147] T Panagiotopoulos and T Georgakopoulou. Epidemiology of rubella and congenital rubella syndrome in Greece, 1994-2003. *Euro Surveillance*, 9(4):17–19, apr 2004.

[148] Kimberly M Thompson, Emily A Simons, Kamran Badizadegan, Susan E Reef, and Louis Z Cooper. Characterization of the risks of adverse outcomes following rubella infection in pregnancy. *Risk Analysis*, 36(7):1315–1331, 2016.

[149] J. Bilcke, M. Antillon, Z. Pieters, et al. *Cost-effectiveness of routine and campaign use of typhoid Vi-conjugate vaccine in Gavi-eligible countries: a modelling study*. Lancet Infect Dis, 2019.

[150] J. H. Kim, V. Mogasale, J. Im, et al. Updated estimates of typhoid fever burden in sub-Saharan Africa. *Lancet Glob Health*, 5, 2017.

[151] Z. Pieters, N. J. Saad, M. Antillon, V. E. Pitzer, and J. Bilcke. *Case fatality rate of enteric fever in endemic countries: A systematic review and meta-analysis*. Clin Infect Dis, 2018.

[152] J. A. Salomon, T. Vos, D. R. Hogan, et al. Common values in assessing health outcomes from disease and injury: disability weights measurement study for the Global Burden of Disease Study 2010. *Lancet*, 380(9859):2129–43, 2012.

[153] T Garske, MD Van Kerkhove, S Yactayo, O Ronveaux, RF Lewis, JE Staples, W Perea, NM Ferguson, and Yellow Fever Expert Committee. Yellow Fever in Africa: estimating the burden of disease and impact of mass vaccination from outbreak and serological data. *PLoS Medicine*, 11(5):e1001638, may 2014.

[154] Katy A. M. Gaythorpe, Arran T. P. Hamlet, Kevin Jean, Daniel Garkauskas Ramos, Laurence Cibrelus, Tini Garske, and Neil M. Ferguson. The global burden of yellow fever. *medRxiv*, oct 2020.

[155] Michael A Johansson, Pedro F C Vasconcelos, and J Erin Staples. The whole iceberg: estimating the incidence of yellow fever virus infection from the number of severe cases. *Transactions of the Royal Society of Tropical Medicine and Hygiene*, 108(8):482–487, aug 2014.

[156] Joseph L Servadio, Claudia Muñoz-Zanzi, and Matteo Convertino. Estimating case fatality risk of severe Yellow Fever cases: systematic literature review and meta-analysis. *BMC infectious diseases*, 21(1):1–12, 2021.

[157] Gretchen A Stevens, Leontine Alkema, Robert E Black, J Ties Boerma, Gary S Collins, Majid Ezzati, John T Grove, Daniel R Hogan, Margaret C Hogan, Richard Horton, et al. Guidelines for accurate and transparent health estimates reporting: the GATHER statement. *The Lancet*, 388(10062):e19–e23, 2016.

[158] Austin Carter, William Msemburi, So Yoon Sim, Katy AM Gaythorpe, Ann Lindstrand, and Raymond CW Hutubessy. Modeling the Impact of Vaccination for the Immunization Agenda 2030: Deaths Averted Due to Vaccination Against 14 Pathogens in 194 Countries from 2021-2030. 2030, 2021.
